# Regions of common inter-individual DNA methylation differences in human monocytes: genetic basis and potential function

**DOI:** 10.1186/s13072-017-0144-2

**Published:** 2017-07-26

**Authors:** Christopher Schröder, Elsa Leitão, Stefan Wallner, Gerd Schmitz, Ludger Klein-Hitpass, Anupam Sinha, Karl-Heinz Jöckel, Stefanie Heilmann-Heimbach, Per Hoffmann, Markus M. Nöthen, Michael Steffens, Peter Ebert, Sven Rahmann, Bernhard Horsthemke

**Affiliations:** 1 Genome Informatics, Institute of Human Genetics, University of Duisburg-Essen, University Hospital Essen, Essen, Germany; 2 Institute of Human Genetics, University of Duisburg-Essen, University Hospital Essen, Hufelandstraße 55, 45147 Essen, Germany; 3 Institute for Clinical Chemistry and Laboratory Medicine, University Hospital Regensburg, Regensburg, Germany; 4 Institute of Cell Biology, University Hospital Essen, Essen, Germany; 5 Institute of Clinical Molecular Biology, Kiel University, University Hospital, Kiel, Germany; 6 Institute of Medical Informatics, Biometry and Epidemiology, University Hospital Essen, Essen, Germany; 7 Institute of Human Genetics, School of Medicine, University Hospital of Bonn, University of Bonn, Bonn, Germany; 8 Department of Genomics, Life and Brain Center, University of Bonn, Bonn, Germany; 9 Institute of Medical Genetics and Pathology, University Hospital Basel, Basel, Switzerland; 10 Human Genomics Research Group, Department of Biomedicine, University of Basel, Basel, Switzerland; 11 Research Division, Federal Institute for Drugs and Medical Devices (BfArM), Bonn, Germany; 12 Max Planck Institute for Informatics, Saarland Informatics Campus, Saarbrücken, Germany; 13 Saarbrücken Graduate School of Computer Science, Saarland Informatics Campus, Saarbrücken, Germany

**Keywords:** DNA methylation, Haplotype, Genome-wide association study, Differentially methylated regions, Inter-individual variability, Allele-specific methylation, Whole genome bisulfite sequencing, SNP genotyping, Methylation array

## Abstract

**Background:**

There is increasing evidence for inter-individual methylation differences at CpG dinucleotides in the human genome, but the regional extent and function of these differences have not yet been studied in detail. For identifying regions of common methylation differences, we used whole genome bisulfite sequencing data of monocytes from five donors and a novel bioinformatic strategy.

**Results:**

We identified 157 differentially methylated regions (DMRs) with four or more CpGs, almost none of which has been described before. The DMRs fall into different chromatin states, where methylation is inversely correlated with active, but not repressive histone marks. However, methylation is not correlated with the expression of associated genes. High-resolution single nucleotide polymorphism (SNP) genotyping of the five donors revealed evidence for a role of *cis*-acting genetic variation in establishing methylation patterns. To validate this finding in a larger cohort, we performed genome-wide association studies (GWAS) using SNP genotypes and 450k array methylation data from blood samples of 1128 individuals. Only 30/157 (19%) DMRs include at least one 450k CpG, which shows that these arrays miss a large proportion of DNA methylation variation. In most cases, the GWAS peak overlapped the CpG position, and these regions are enriched for CREB group, NF-1, Sp100 and CTCF binding motifs. In two cases, there was tentative evidence for a *trans*-effect by KRAB zinc finger proteins.

**Conclusions:**

Allele-specific DNA methylation occurs in discrete chromosomal regions and is driven by genetic variation in *cis* and *trans*, but in general has little effect on gene expression.

**Electronic supplementary material:**

The online version of this article (doi:10.1186/s13072-017-0144-2) contains supplementary material, which is available to authorized users.

## Background

Allele-specific DNA methylation occurs at distinct regions of the mammalian genome: (1) at imprinted loci as a result of genomic imprinting in the germline, (2) at gene promoters on the silent X chromosome in females as a result of X inactivation during early embryogenesis and (3) at non-imprinted autosomal loci as a consequence of genetic variation in *cis* (haplotype-dependent allele-specific methylation, hap-ASM [1–5]). In contrast to genomic imprinting and X inactivation, which always result in methylation of one allele in each cell of an individual, hap-ASM can be present on both alleles, on just one allele or on none of the alleles, dependent on the individual’s genotype. In practice, however, most often DNA methylation levels other than 100, 50 or 0% are observed. This is because of extensive cell-to-cell heterogeneity (epigenetic mosaicism) as well as tissue heterogeneity. Epigenetic mosaicism means that even in a pure, isogenic cell population, cells differ from each other with respect to DNA methylation at a given locus, probably because the two alleles of a single nucleotide polymorphism (SNP) do not always dictate or prevent methylation of their genomic environment, but only increase or decrease the possibility that methylation occurs. This probability may even vary across tissues and may also be affected by environmental factors. As a consequence of this, a given genotype can be associated with multiple epigenotypes.

It has been suggested that hap-ASM may contribute to phenotypic variation, although there is no direct evidence for this to date. Indirect evidence comes from methylation quantitative trait loci (mQTL) studies, expression quantitative trait loci (eQTL) studies and genome-wide association studies (GWASs) [2, 6, 7]. These investigations have shown correlations between DNA sequence, methylation levels, gene expression levels and phenotypic traits, but it remains to be determined whether hap-ASM mediates the effect of DNA sequence variation on gene expression levels and phenotypic traits (active role), whether it stabilizes gene expression levels that have been brought about by SNP-sensitive transcription factors (passive role), or whether it occurs on certain haplotypes without having a function (no role).

Most mQTL studies were performed with methylation sensitive microarrays such as the Illumina 450k array. Owing to the low probe density of this array (it assays only 450,000 CpGs (1.6%) out of 28,000,000 CpGs), only single CpG sites or a combination of CpGs scattered over regions with poorly defined borders have been studied. Most hap-ASM studies used bisulfite sequencing (Sanger sequencing of subcloned bisulfite PCR products), which provides base-pair resolution, but were targeted at a few candidate regions only. A vast improvement in the field are techniques which enrich for all genomic regions known to impact gene regulation (hybrid capture kits; see for example [2]) or for all highly methylated regions (antibody-based approaches; see for example [8]). An unbiased survey of all differentially methylated regions, however, requires whole genome bisulfite sequencing (WGBS). WGBS has recently become the gold standard of genome-wide methylation analysis, but owing to the high costs involved, most often only a small number of samples are studied with this technique. However, one advantage of using allele-specific analysis compared to the QTL approaches is the smaller sample size requirements [9]. Nevertheless, sophisticated bioinformatic tools are necessary to reliably detect differentially methylated regions (DMRs) in a limited number of datasets, and candidate DMRs have to be validated by array-based techniques and/or targeted approaches.

Hap-ASM can seriously confound comparative methylome analyses in humans, if the samples are from different individuals. We have recently observed that DNA methylation differences between individuals may be larger than between distinct cell types [10], which has prompted us to identify and characterize inter-individual differences in DNA methylation in a more systematic way. For identifying regions showing common allele-specific DNA methylation, we have searched for blocks of co-varying CpGs (COMETs; [11]) that occur in only two states/epialleles (mainly methylated or mainly unmethylated) in each cell. At such a DMR, any individual has one of three epigenotypes: methylated/methylated, methylated/unmethylated, or unmethylated/unmethylated. To reduce the number of possible confounders in the DMR discovery phase, we restricted our analysis to a single cell type (monocytes) and used cells isolated by the same procedure (elutriation) from donors of the same sex (males). Based on epigenomic datasets generated by the same laboratory and bioinformatics pipeline according to standards set by the International Human Epigenetic Consortium (IHEC), this approach has enabled us to identify a significant number of high-confident DMRs and to link them to chromatin states and genetic variation.

## Results

### Identification of differentially methylated regions (DMRs)

**Fig. 1:**
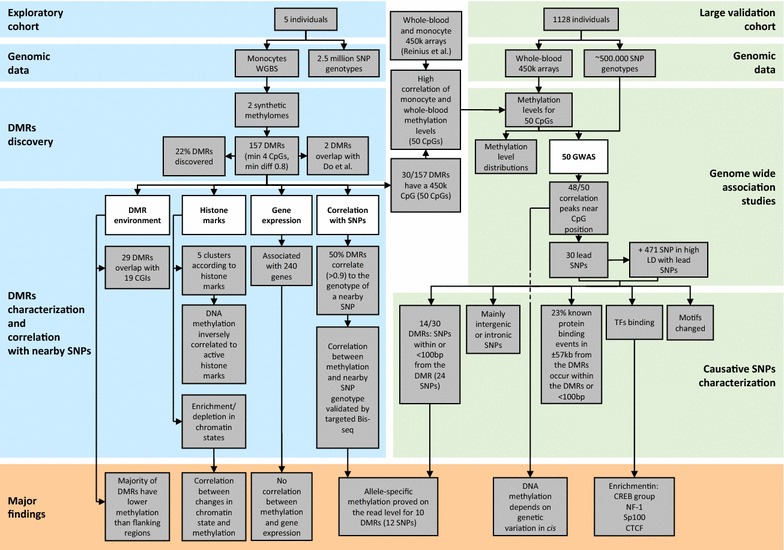
Overview of the study

Our previous DNA methylation analysis [10] had been performed in human monocytes and macrophages from two male donors (Hm03 and Hm05; for a cluster analysis see Additional file 1). For identifying inter-individual DNA methylation differences in human monocytes in a systematic way (see Fig. 1 for an overview of our study), we included three additional WGBS datasets produced by our laboratory (M55900 and Hm01 [12] as well as Hm02 (this study; for quality parameters of the methylomes see Additional file 2). In addition, we downloaded five publicly available IHEC WGBS datasets on human monocytes from other male donors, two from the BLUEPRINT consortium and three from the Canadian Epigenetics, Environment and Health Research Consortium (CEEHRC). A principle component analysis (PCA; Additional file 3), however, revealed that the data are very heterogeneous: While our five methylomes fall right on top of each other, the other methylomes are very different from each other and from our methylomes. The differences are probably due to the use of different cell purification methods, WGBS library preparation protocols, sequencing chemistries and bioinformatics pipelines. Therefore, we proceeded only with our five methylomes.

**Fig. 2:**
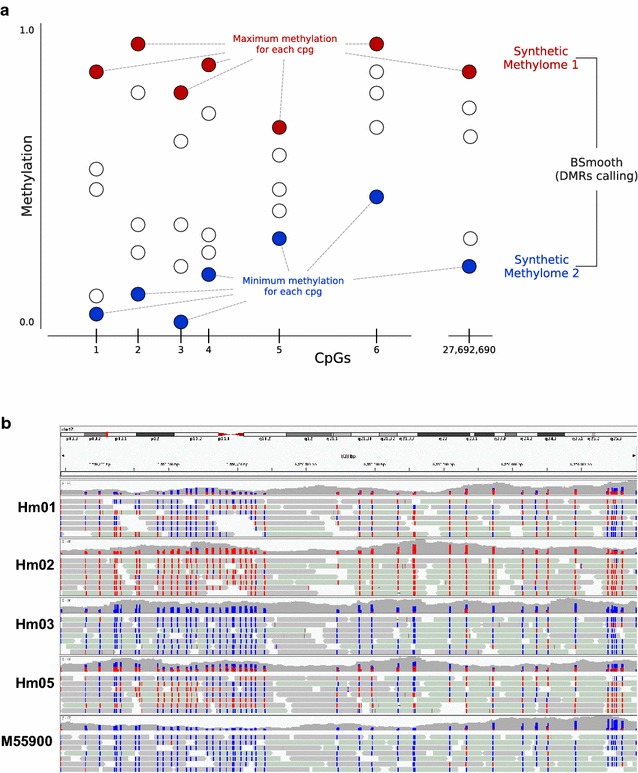
Detection of DMRs. **a** Scheme of the generation of synthetic methylomes. **b** Representative example of an inter-individual DMR (DMR128, chr17:6558143-6558981) visualized in the IGV browser. Only a subset of reads is shown for each individual (Hm01, Hm02, Hm03, Hm05 and M55900). *Red* methylated CpG; *blue* unmethylated CpG

For identifying regions of common inter-individual DNA methylation differences, we devised a novel bioinformatic strategy: We created two synthetic methylomes, one with the highest methylation value of each CpG in the five samples and one with the lowest methylation value (Fig. 2a). We then used a modified version of Bsmooth [13] to detect differentially methylated regions (DMRs) between the two synthetic methylomes (see “Methods” section). Defining a DMR as a region of at least 4 CpGs with a methylation level difference of at least 0.8, we identified 157 DMRs (*p* < 0.001; Additional file 4). The threshold of 0.8 implies that a DMR is homozygously methylated in at least one individual and homozygously unmethylated in at least another individual, i.e., methylation differences in this region are very common.

The DMRs cover 1165 CpGs, have a size range of 9 to 1495 bp and encompass 4 to 44 CpGs (Additional files 4 and 5). The region with the highest number of CpGs is shown in Fig. 2b. Five DMRs have previously been reported by others: Two regions (DMR87 and DMR134) overlap previously designated hap-ASM DMRs [2], two DMRs contain a previously reported SNP mQTL (DMR25—rs6760544 [5], and DMR104—rs11158727 [6]), and one DMR contains a previously reported ASM–SNP (DMR24—rs1530562 [14]). The majority of the DMRs are either intergenic (79/157) or intronic (57/157), while 13/157 span over an exon–intron boundary and 7/157 are located within an exon (Additional file 4). In comparison with randomly chosen regions, intergenic DMRs are highly overrepresented, whereas intragenic and exonic DMRs are highly underrepresented (Additional file 6).

Since non-imprinted autosomal CpG islands (CGIs) are typically unmethylated, we find it surprising that 29/157 DMRs overlap a CpG island (CGI), although in general CGI-DMRs are also underrepresented (Additional file 6). Most of these CGI-DMRs (19/29) are intragenic (either intron (*n* = 8), exon (*n* = 5) or intron–exon boundary (*n* = 6)). In 24 of these CGI-DMRs, all CpGs are within a CGI, in 4 cases there is a partial overlap with at least 50% of the CpGs belonging to a CGI, and in one case, the CGI is within the DMR. In some cases, closely linked DMRs affect the same CGI, probably because the DMR detection algorithm separated a large DMR into two or more DMRs. In total, 19 CGIs overlap a DMR, 11 of which are intragenic. Some of the CGIs are orphan CGIs, i.e., they are not associated with an annotated transcription start site [15].

We analyzed five samples, and our stringent settings allowed us to detect regions with common methylation differences. We expect that increasing the sample number could lead to the discovery of additional DMRs with rarer epialleles. Thus, we asked how many DMRs with a minor epigenetic allele frequency >0.05 might be present in the human population. Assuming that DNA methylation is allele-specific in these regions (for validation see below) and that the Hardy–Weinberg equilibrium applies in this situation, we estimate that there are 692 such DMRs. Of these, we have detected 23%.

### Genomic environment of the DMRs

**Fig. 3:**
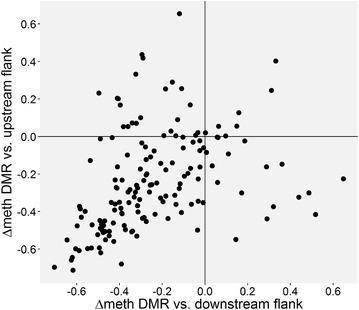
Differences in DNA methylation of the DMRs and the upstream and downstream flanking regions. Most of the DMRs are flanked by regions with higher methylation levels (quadrant at *lower left*)

Since WGBS provides DNA methylation levels of all CpGs, we could investigate whether certain haplotypes (see below) act to decrease DNA methylation in a highly methylated domain or act to increase DNA methylation in a lowly methylated domain. For this, we compared the mean methylation level of the DMRs in the five donors to that of their flanking regions. In order to avoid border effects, we ignored the next three CpGs on each side of the DMRs and analyzed the following 10 CpGs. We observed that the mean methylation level of the DMRs is 0.49, which is close to the expected methylation level if on average methylated and unmethylated alleles occur at a similar frequency in the five donors. In contrast, both upstream and downstream flanking regions have a much higher level of methylation (average 0.72), which is highly significant (*p* = 4.86 × 10^−20^ and *p* = 1.71 × 10^−21^, respectively, Wilcoxon rank-sum test) (Additional file 4). Indeed, the vast majority of DMRs (107) have mean methylation levels lower than the two adjacent regions, while only 7 have higher methylation than both surrounding sequences (Fig. 3). In 40 DMRs, the methylation is intermediate from that of both flanking regions. For the remaining 3 DMRs, data are lacking for one of the flanks.

### Chromatin states of the DMRs

**Fig. 4:**
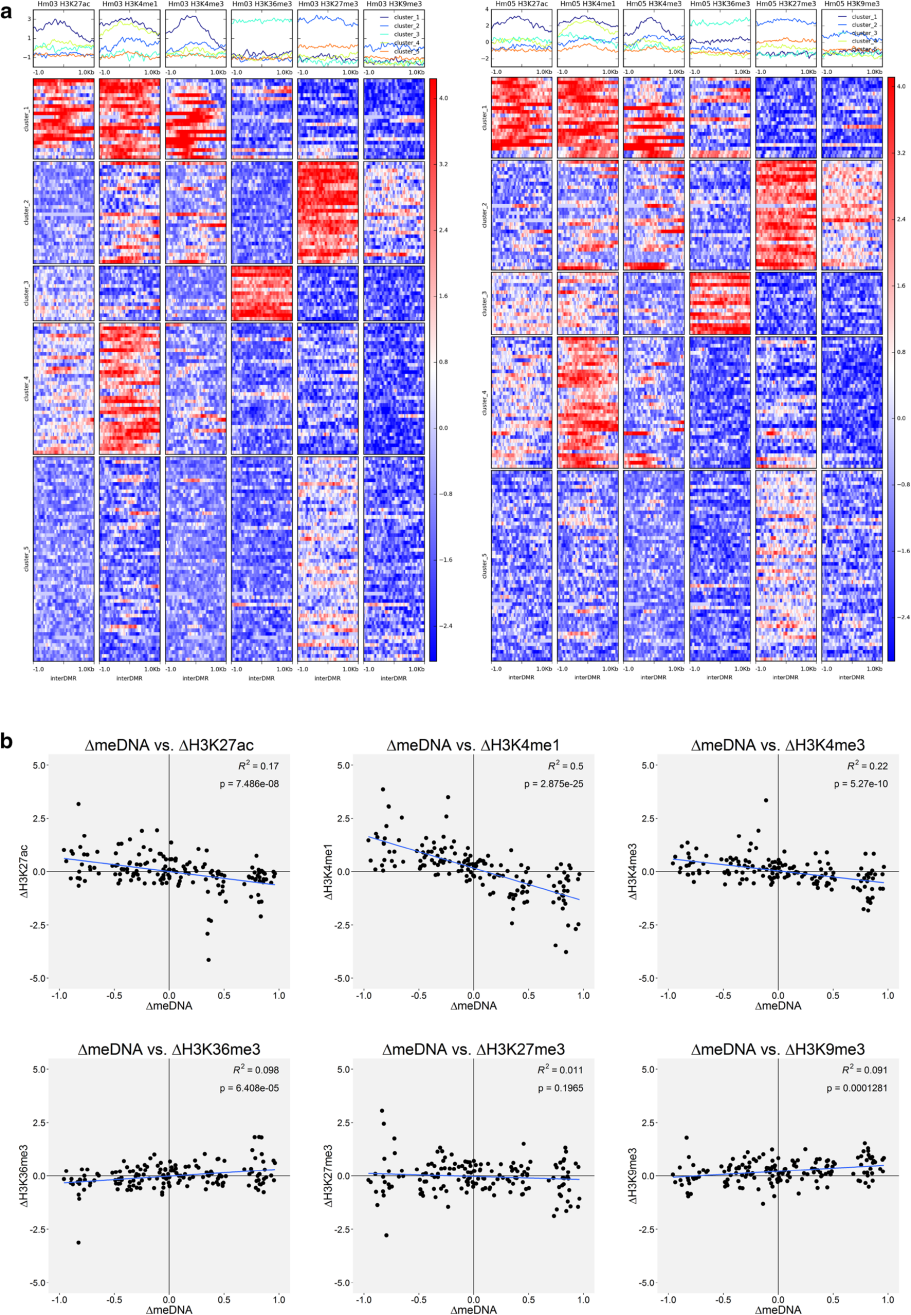
Histone modifications of 2 kb regions centered on the 157 inter-individual DMRs. **a** Heatmaps of histone modification signals for Hm03 (*left*) and Hm05 (*right*). Heatmaps show log2 ratio ChIP signal over input for six different histone modifications. **b** Scatter plots showing difference in histone modification signals between Hm05 and Hm03 as a function of methylation differences between the two donors. Active histone marks are inversely correlated with DNA methylation (linear regression)

For investigating the functional significance of the DMRs, we looked at six histone modifications (H3K4me1, H3K4me3, H3K27ac, H3K36me3, H3K27me3 and H3K9me3), which had been determined in the same monocyte samples from donors Hm03 and Hm05 [10]. Using the *k*-means algorithm (with *k* = 5 classes) to cluster 2 kb sequences centered on the DMRs according to the ChIP signal across all six histone marks, we found that the DMRs have different histone modifications patterns (Fig. 4a). Clusters 1 and 4 are enriched for H3K27ac (albeit weakly in cluster 4) and H3K4me1, cluster 1 also for H3K4me3. These marks are indicative of active enhancers and promoters. Cluster 2 is strongly enriched for the repressive mark H3K27me3 and weakly enriched for the repressive mark H3K9me3. Cluster 3 is weakly enriched for H3K27ac and strongly enriched for H3K36me3, suggesting that these DMRs are transcribed elements. Cluster 5 is weakly enriched for the repressive mark H3K27me3. In summary, approximately 50% of the DMRs (84/157 in Hm03 and 82/157 in Hm05) carry strong or weakly repressive histone marks (clusters 2 and 5). The same is true for the subset of the 29 CGI-DMRs: 11/29 CGI-DMRs belong to cluster 2 in both donors, and 5/29 CGI-DMRs belong to cluster 2 in one of the two donors (Additional file 4). Most of the DMRs belonging to histone cluster 1 are intragenic (14/22 Hm03, 14/19 Hm05). On the other hand, most of the DMRs belonging to histone cluster 5 are intergenic (35/56 Hm03, 37/52 Hm05).

Independent clustering was performed for Hm03 and Hm05, since the two donors differ in the DNA methylation values of the DMRs. When we looked at the correlation between differences in DNA methylation levels and differences in histone modification levels between the two donors, we found that DNA methylation was inversely correlated with the active histone marks (linear regression), although the differences in histone modifications were small, but it was not correlated with the repressive histone marks (Fig. 4b and Additional file 7). In summary, this analysis suggests that the DMRs have different chromatin states and are more correlated to active than to repressive histone marks.

Based on the combination of the different histone marks in Hm03 and Hm05 monocytes, we segmented the genome into 18 chromatin states with the help of ChromHMM [16] and investigated whether certain chromatin states are over- or underrepresented (Additional file 8). We found that in both datasets 1_TssA, 5_Tx and 17_ReprPCWk were underrepresented and that 16_ReprPC and 2_TssFlnk or 4_TssFlnkD were overrepresented. Next, we investigated whether DMRs having (1) the same chromatin states in Hm03 and Hm05, or (2) different states in both donors, have similar distributions of absolute methylation differences between the two donors. In fact, the distribution is significantly different: In the DMRs that have different chromatin states in the two donors, methylation differences are higher compared to the others (*p* = 2.33 × 10^−5^, Wilcoxon rank-sum test). As shown by the violin plots in Additional file 9, there are many DMRs with the same chromatin state and the same level of DNA methylation in the two donors (methylation difference <0.1), but there are very few DMRs with different chromatin states and the same DNA methylation. The relative abundance of DMRs with different chromatin states and methylation differences around 0.4 may be explained by homozygosity for a state in one donor and heterozygosity in the other donor, while differences around 0.8 may occur in DMRs where the two donors are homozygous for opposite states. In summary, these findings show that there is a correlation between DNA methylation and active chromatin states.

### Location of the DMRs and putative target genes

**Fig. 5:**
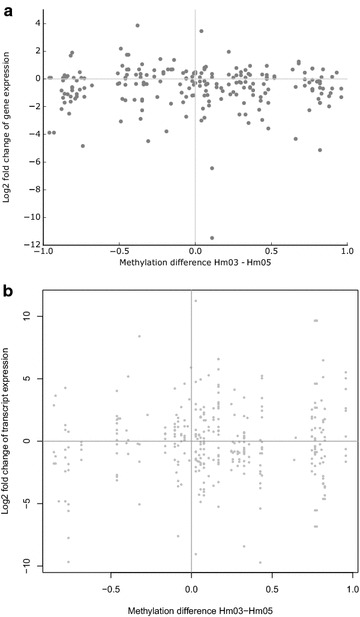
Correlation between DNA methylation and gene expression. **a** Scatter plot of the differences in gene expression levels of the putative target genes identified by GREAT and the differences in DMR methylation in donors Hm03 and Hm05. **b** Scatter plot of the differences in transcript isoform levels of genes harboring a DMR and the differences in methylation of the 77 intragenic DMRs

The analysis of the 157 DMRs with Genomic Regions Enrichment of Annotations Tool (GREAT), which identifies *cis*-regulatory elements and their target genes, showed that 155/157 DMRs are associated with at least one gene and that in the majority of cases these are far away (see Additional files 4 and 10). In total, 240 different genes were identified. There was no significant enrichment of GO terms. The expression levels of these genes in donors Hm03 and Hm05 were not different from those genes that are not associated with a DMR (17,544; *p* = 0.45, Wilcoxon rank-sum test) (Additional file 11). As shown in Fig. 5a, there is no correlation between the differences in gene expression levels and the differences in methylation levels in these donors. The same is also true for the subset of genes that are associated with a DMR belonging to the histone modification clusters 1, 3 or 4 (active and transcribed DMRs), the subset of genes that are associated with a DMR which has a different chromatin state in donors Hm03 and Hm05, or the subset of genes that harbor a DMR (data not shown).

Since DNA methylation might affect alternative transcript initiation or splicing without changing total mRNA levels [17], we further investigated the genes harboring a DMR. As shown in Fig. 5b, there was no significant correlation between differences in methylation levels of the 77 intragenic DMRs and differences in transcript isoform expression of the host genes (*r* ^2^ = 0.006, *p* = 0.085).

Due to the fact that the gene list used by GREAT does not include all long non-coding RNA genes, we queried the database for annotated human lncRNAs (LNCipedia) to identify lncRNA genes overlapping the DMRs. We found nine such genes (Additional file 4), and the two genes that are expressed in monocytes have equal RNA levels in both donors.

### Correlation of DMR methylation levels with nearby SNPs

Next, we asked whether DNA polymorphisms within the DMRs or close by could be responsible for the inter-individual differences in DNA methylation. We genotyped the five donors for 2.5 million SNPs and found that 82/157 (52%) DMRs have a methylation level that is highly correlated (score > 0.9; see Methods) with the genotype of at least one nearby SNP (±6 kb from the center of the DMR; Additional file 12; for details see Methods). In 21/157 DMRs, that SNP is located within the corresponding DMR, and in 18/157 it is located <200 bp from the corresponding DMR border.

### Validation of selected DMRs

**Fig. 6:**
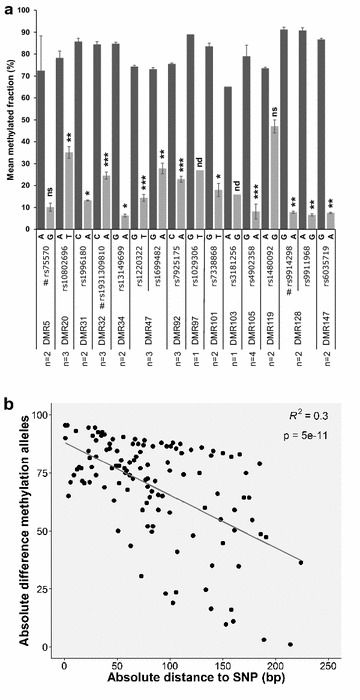
Allele-specific DNA methylation. **a** Allele-specific DNA methylation for 14 DMRs (16 SNPs) performed by targeted deep bisulfite sequencing and sorting of reads by SNP allele. Average CpG methylated fractions in reads containing one or the other allele. Results are mean ± SD from 1 to 4 independent donor samples heterozygous for the correlating SNPs. *p value < 0.05; **p value < 0.01; ***p value < 0.001; ns, not significant (two-tailed paired Student’s *t* test); nd, not done; #, SNPs discovered after GWAS. **b** Scatter plot showing the absolute differences in methylation between reads containing one or the other SNP allele as a function of the absolute distance to the SNP. *Each dot* represents one CpG

We selected seven DMRs for validation that matched each of the following criteria: (1) SNP correlation score >0.9, (2) at least one CpG present on 450k arrays and (3) a methylation level of 33–67% in at least one of the five donors. Validation was performed by targeted deep bisulfite sequencing of four monocyte samples used for WGBS (Hm01, Hm02, Hm03 and Hm05) as well as two additional samples (Hm06 and Hm10), whom we genotyped for the 13 SNPs highly correlated with those DMRs (Additional file 12). For 6/7 DMRs, we observed a correlation between the DMR methylation levels and the genotype of at least one of the correlating SNPs (Additional file 13). In these cases, the homozygotes showed either the highest or lowest DMR methylation level, depending on the SNP allele, while the heterozygotes presented intermediate levels of methylation. For DMR12, which was no longer correlated with SNP rs692963 when two additional individuals where analyzed, it is possible that a correlating SNP lies >6 kb from the center of the DMR (as shown below, it is indeed). Regarding DMR128, in which the correlating SNP (rs9911968) is located within the DMR, we further analyzed heterozygotes for this SNP and calculated the methylation levels for reads containing the A or the G allele. We observed a significant difference in the methylation levels depending on the SNP allele present in the read, with the vast majority of the A allele containing reads being methylated, while the reads containing the G allele were unmethylated (Fig. 6a and Additional file 14). This demonstrates that DMR128 is subject to allele-specific methylation.

To verify that the same is true for other DMRs, we selected 11 regions that matched the criteria (1) and (3) above and the criterion that the correlating SNP locates within the DMR or in close vicinity (<200 bp from the corresponding DMR border). We performed targeted deep bisulfite sequencing of samples heterozygous for the corresponding correlating SNPs. For 8/11 DMRs, there are statistically significant differences between the two alleles (*p* < 0.05, two-tailed paired Student’s t test), proving on the read level that allele-specific methylation occurs in these DMRs (Fig. 6a and Additional file 14), and validating the methodology we used to discover the DMRs. For 2/3 DMRs that fail to reach statistical significance (DMR97 and DMR103), we had only one sample. We also observed that the difference in methylation between the methylated and the unmethylated allele diminishes with the distance to the correlated SNP (Fig. 6b), reassuring the relevance of these SNPs genotype on the methylation levels.

### Genome-wide association studies (GWAS)

**Fig. 7:**
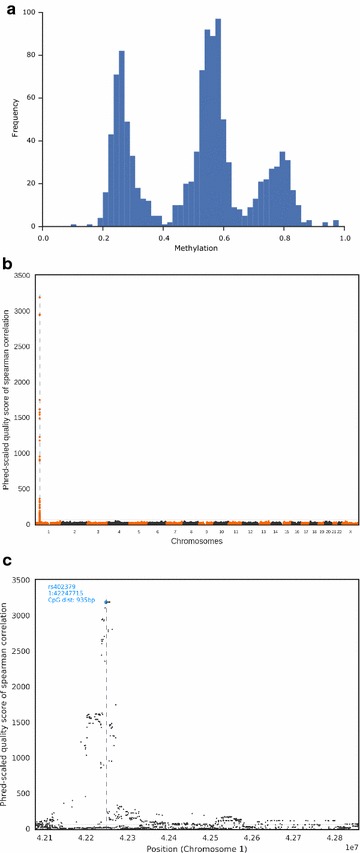
Representative results of the GWASs. One example is shown for the DMR5 CpG included in the Illumina 450k array (position 1:42248998). **a** Histogram of methylation level distribution. **b** Manhattan plot. *Dashed vertical line* DMR position. *Horizontal line* GWAS significance threshold. **c** Zoom with imputed SNPs. *Blue* lead-SNP. *Dashed vertical line* DMR position. *Horizontal line* GWAS significance threshold

To validate the association between SNP genotypes and DNA methylation states in an independent and larger cohort, we investigated SNP and DNA methylation data of 1128 probands from the Heinz-Nixdorf Recall Study [18, 19]. In this cohort, DNA methylation levels had been determined in blood DNA with the help of Illumina 450k microarrays. Only 30/157 (19%), DMRs include one or more Illumina 450k CpGs (total: 50 CpGs), which shows that these arrays miss a large proportion of DNA methylation variation. In at least 29/50 cases, the distribution of the methylation levels showed three distinct peaks, suggesting that there are two epialleles (high and low methylation) (Fig. 7a and Additional file 15).

First, we checked whether monocyte and whole blood methylation levels were correlated. For this, we analyzed previously published Illumina 450k microarray data generated for whole blood and CD14^+^ monocytes samples from six healthy male donors [20]. The comparison of monocyte and whole blood methylation levels for the 50 CpGs revealed a high correlation (>0.92) in all individuals (Additional file 16).

For each of the 50 CpGs, we performed a GWAS with ~500,000 SNP, in which CpG methylation was treated as a quantitative trait. In 47/50 cases, there was a single correlation peak, which overlapped the CpG position (Fig. 7b, c; Additional files 17 and 18). For the CpG in DMR94 on chromosome 12, there was a correlation peak at the CpG position (*p* = 1.59 × 10^−15^) and at a locus on chromosome 19 (*p* = 1.40 × 10^−41^). For the CpG in DMR53 on chromosome 6, there was a single correlation peak on chromosome 7 (*p* = 6.01 × 10^−11^). We did not find any evidence for misannotation or cross-hybridisation of the array probes in these cases, but noted that the two GWAS peaks located on different chromosomes than the DMRs overlapped with genes coding for KRAB zinc finger transcription factors (*ZNF573* and *ZNF92*, for the DMR94 and DMR53 GWAS peaks, respectively). Since a *p* value threshold of 5 × 10^−8^ has become a standard for genome-wide significance in GWAS, the extremely low *p* values at the *ZNF573* and *ZNF92* loci point to a *trans*-acting effect.

For each GWAS, the SNP at the CpG locus with lowest *p* value (in most cases *p* < 10^−2000^) was designated as lead-SNP (total *n* = 30). Using this genome-wide approach, we were able to confidently detect correlating SNPs outside the 12-kb window, e.g., in DMR12, for which the putatively correlated SNP detected in a 12-kb window failed validation in six individuals, the lead-SNP is actually located 40 kb from the DMR (Additional file 18). We used HaploReg [21] to retrieve SNPs in high linkage disequilibrium (LD, *r* ^2^ > 0.8) to the lead-SNPs (total *n* = 471), which are located within the corresponding DMR or up to ~116 kb from it, and mainly in intronic or intergenic regions (Additional file 19). For three of the DMRs validated previously, we confirmed the occurrence of ASM at the read level at SNPs in high LD with the lead-SNPs (Fig. 6a). These findings validate and extend the exploratory study described above.

### Analysis of transcription factor-binding sites in and around the DMRs

Next, we analyzed whether the lead-SNPs and the highly correlated SNPs (total *n* = 501) might affect transcription factor-binding sites. Exploration of SNP annotation data from HaploReg database revealed that 23% of known protein binding events (Encode ChIPseq data) occur within the DMRs or <100 bp away (Additional file 20). The remaining events occur over a region up to 57 kb away from the DMR. The top five proteins found to bind within the DMRs or in close vicinity (<100 bp) are CTCF, CMYC, CEBPB, RAD21 and SMC3 (Additional file 19).

TRANSFAC analysis showed that the SNP regions are enriched for CREB group, NF-1, Sp100 and CTCF binding motifs (Additional file 21), and further analysis of their HaploReg annotations revealed that most of the SNPs are likely to alter regulatory motifs (Additional file 19).

## Discussion

The use of whole genome bisulfite sequencing in human monocytes as part of full IHEC epigenomes and a novel bioinformatic approach has enabled us to identify and characterize regions of common inter-individual differences in DNA methylation at base-pair resolution, where allele-specific methylation is mainly caused by *cis*-acting genetic variation in transcription factor-binding sites. In two cases, we have obtained tentative evidence for *trans*-acting genetic variation in KRAB zinc finger genes. High-resolution WGBS has also allowed us to determine the methylation level of the genomic environment of these regions: Most of them are flanked by highly methylated DNA, which shows that certain haplotypes act to decrease DNA methylation in a highly methylated domain. Almost none of the DMRs described here has been identified before, and unexpectedly some overlap with a CpG island (CGI). Overall, however, gene promoters are underrepresented among the DMRs. Differences in DNA methylation are correlated with differences in active histone modifications and chromatin states, but in general not with differences in expression levels of the putative target genes, suggesting that other regulatory mechanisms are preponderant over DNA methylation in maintaining expression levels.

It is not possible to determine the exact number of DMRs, because this number obviously depends on the criteria used for DMR detection. Using WGBS data from five individuals and stringent thresholds, we have detected 157 inter-individual DMRs in monocytes and estimate that there are 692 regions with a minor epiallele frequency >0.05 in the human population. Interestingly, Do et al. arrive at similar figure (*n* = 792), but there is hardly any overlap between their DMRs and ours (only two). Most probably, the DMRs identified by us are not targeted by the Agilent SureSelect Methyl-seq capture kit used by Do et al. [2], which queries only 3.7/28 million CpGs (13.2%). Since this kit focuses on regions where methylation is known to impact gene regulation, our DMRs appear to lie in regions of unknown function (see also below). On the other hand, we may have missed the DMRs identified by Do et al., because they did not pass our stringent criteria for significance and/or different tissues were used.

By defining a DMR as a region with a methylation difference between the two synthetic methylomes of at least 0.8, we have identified regions of common methylation differences which are characterized by allele-specific DNA methylation in the majority of cells. Regions which show allele-specific DNA methylation in only a fraction of cells would not have passed the 0.8 threshold. This is one reason why the number of hap-ASM regions identified in this way is much smaller than the number of loci typically identified in mQTL studies (see for example [7]). In mQTL studies, where the genotype of each tested SNP is correlated with the methylation level of each tested CpG, CpGs often have normalized methylation values on a continuous scale between 0.0 and 1.0. At the level of individual cells, however, DNA methylation (unlike mRNA levels, for example) cannot be a quantitative trait, because a given CpG in a DNA molecule can only be methylated or unmethylated, allowing for three discrete epigenotypes per cell only. Therefore, mQTLs studies primarily measure the proportion of cells with one or two methylated alleles or—in other words—the probability that a CpG becomes methylated, rather than allelic methylation differences *per se*. Mean methylation values across a region with several CpGs could be a quantitative trait, if the methylation of the individual CpGs were poorly correlated. In our validation studies based on deep bisulfite sequencing, we have not observed such heterogeneous patterns.

We note, similar to Do et al. [2], that most of the hap-ASM regions are not covered by the Illumina 450k array. In our case, the array missed 80% of the DMRs. For almost all of the tested DMRs that did not have a CpG on the 450k array, allele-specific methylation was proven to occur on the read level at SNPs within or in the close vicinity to the DMR. Thirty of the 157 DMRs could be studied in a large cohort on 450k methylation arrays. In many of these cases, we saw a trimodal distribution of the methylation values, reflecting the three possible epigenotypes and indicating low epigenetic mosaicism. In the other cases, the CpG tested may not be representative for the DMR, because it is close to the border of the DMR, for example.

In our GWAS studies, we found that the methylation levels were significantly correlated with the genotype of nearby SNPs, often with *p* values <10^−2000^, which also validates these DMRs. In two cases (DMR53 and DMR94), we obtained tentative evidence for the existence of *trans*-acting loci. Interestingly, in both cases the GWAS peak was over KRAB zinc finger transcription factors genes, namely *ZNF92* and *ZNF573*. Unfortunately, the two proteins are poorly characterized, but KRAB zinc fingers are known to interact—among other proteins—with TRIM28, which plays a role in maintaining DNA methylation [22–24]. This finding certainly requires validation in another cohort as well as more detailed molecular studies.

For investigating the possible role of the DMRs, we made use of the histone modification and gene expression data that we have on the Hm03 and Hm05 monocytes as part of the full IHEC epigenomes. We find that the DMRs lie in regions with different chromatin states including active and repressive chromatin and that they are enriched for regions flanking transcription start sites (TssFlnk), but depleted for strong transcription (Tx), active transcription start sites (TssA) and weakly repressed sites (ReprPCwk). With regard to active chromatin, we note that McClay et al., Do et al. and Cheung et al. also observed that mQTLs are enriched for TssFlnk regions [1, 2, 7]. The correlated SNP regions are bound by CTCF, CMYC, CEBPB, RAD21, SMC3 and other transcription factors. Enrichment of CTCF- and RAD21-binding sites in TssFlnkD regions has also been observed by the Epigenome Roadmap Consortium [25]. CTCF, RAD21 and SMC3 play an important role in chromatin architecture [26]. The relevance of CTCF binding for hap-ASM in other DMRs has previously been reported [2, 14, 27]. Together, these data suggest that SNPs cause hap-ASM through affecting the binding of transcription factors to the DNA, most likely mediated through chromatin looping in the case of SNPs located far away from the DMR. However, which SNP and which transcription factor affect DNA methylation is difficult to pinpoint, since genetic variants are often in linkage disequilibrium and may have either a direct influence on transcription factor binding by disrupting the recognition motif, or indirect by affecting cooperative and collaborative transcription factor binding, or altering the chromatin state or conformation affecting the stability of interactions between transcription factors and with DNA [28].

There is a significant inverse correlation between DNA methylation and active histone marks, although the differences in histone modifications are small, but no correlation with repressive histones marks. It is not possible to decide whether the SNPs cause differences in certain histone modifications that favor or hinder DNA methylation, or whether the SNPs cause differences in DNA methylation that affect the recruitment of histone modifying enzymes. Based on what is known about the interplay between DNA and histone modifications [29, 30], we tend to believe that the first scenario is true.

Surprisingly, differences in DNA methylation and histone modifications do not appear to affect gene expression levels. It could be argued that these differences poise the genes for expression in response to external stimuli, which we did not test, but then we would expect that the genes were related to monocyte and macrophage function, which we did not find in our gene ontology analysis. Another possibility is that the methylation differences affect other gene features such as alternative transcript initiation or splicing, which may be true for the subset of intragenic DMRs. Overall, however, there was no correlation between the levels of transcript isoforms and DNA methylation, although it remains possible that in a few exceptional cases the methylation level does affects a transcript isoform. This needs to be investigated further by a series of detailed molecular studies. Still another possibility is that GREAT did not identify the real target genes, which may encode long non-coding RNAs expressed at low levels, especially in large introns and intergenic regions. However, only very few DMRs overlap a lncRNA gene, and most of these are not expressed in monocytes. In summary, we conclude that the majority of the DMRs do not seem to have a strong gene regulatory function under the tested conditions. While this hypothesis may not be welcomed by everybody in the epigenetic field, it is in line with other observations. Gibbs et al. found that only 4.8% of significant mQTLs were also an eQTL [6], and McClay et al. suggested that many mQTLs, especially those located in repressive chromatin, lack functional consequence [7]. Do et al. have also recently hypothesized that only a minority of hap-ASM DMRs are likely to have important effects on gene expression by being located in crucial regulatory regions [9]. However, although some of our DMRs are located in regions with signatures of active enhancers and promoters, they do not seem to affect the expression of target genes. Overall, these results question a major role of hap-ASM in phenotypic variation.

Unexpectedly, a significant fraction of the DMRs overlapping a CGI (see below) carry the repressive histone mark H3K27me3, irrespective of whether they are methylated or not, and there is no correlation with the expression levels of the putative target genes. In imprinted DNA methylation, silent X-associated DNA methylation and cell-type-specific DNA methylation (for the latter see for example [10]), specific DNA sequences are subject to stable transcriptional silencing even in the presence of all of the factors required for their expression [31]. In contrast, a significant proportion of hap-ASM appears to occur in regions where certain haplotypes fail to keep them methylation-free in the presence of the DNA methylation machinery, without affecting gene expression levels.

Since CGIs are almost exclusively unmethylated in all tissue types, regardless of state of expression [31], the observation that 29/157 (~20%) of our DMRs overlap a CGI was unexpected. Assuming that there are 692 such DMRs (see “Results” section) and that a similar fraction of the undetected DMRs overlaps with a CGI, we estimate that ~100/~30,000 CGIs might be affected by hap-ASM in human monocytes. Since hap-ASM shows considerable tissue heterogeneity [2], which substantiates the notion that transcription factors are instrumental in setting up hap-ASM patterns, more than 100 CGIs may be affected. Although the total number of such CGIs is probably small, we find it surprising that hap-ASM affects CGIs at all. It remains to be determined what makes certain CGIs susceptible to hap-ASM. It is probably a combination of transcription factor-binding sites (or a lack thereof) that—on certain haplotypes—fails to protect a CGI against the invasion of methylation from the surrounding region. This is probably true also for other hap-ASM regions. The finding that most of these regions are flanked by highly methylated DNA on both sides suggests that in general DNA-binding factors prevent DNA methylation. In only few cases, the flanking DNA is lowly methylated, and here DNA-binding factors may attract DNA methylation.

## Conclusions

We have identified novel regions of common inter-individual DNA methylation differences in human monocytes. Our study supports and extends the observation that allelic DNA methylation differences can be caused by genetic variation *in cis*. Interestingly, DNA methylation at some loci may also be affected by genetic variation *in trans*, namely at KRAB zinc finger genes. In general, hap-ASM, especially hap-ASM in repressive chromatin domains, appears to have little functional consequences.

## Methods

### Monocytes isolation

Primary human monocytes were isolated from healthy normolipidemic volunteers (Hm02, Hm06 and Hm10) by leukapheresis and counterflow elutriation as described previously [32].

### DNA extraction from monocytes and tissues

DNA was isolated from monocytes using QIAamp columns (Qiagen, Germany) and quantified with a Nanodrop 100 spectrophotometer (Peqlab, Germany).

### Whole genome bisulfite sequencing and analysis

Generation of whole genome bisulfite sequencing data from monocytes obtained from donor Hm02 was performed as described previously [10, 12].

### Detecting DMRs

We used the WGBS datasets from Hm02 and four additional donors (Hm01, Hm03, Hm05 and M55900; see Data retrieval and deposition) to generate two synthetic methylomes, one with the highest methylation level of each CpG in the five samples and one with the lowest methylation level. We modified BSmooth [13] to identify differentially methylated regions with a minimum difference of 0.8 between the two synthetic methylomes. BSmooth is designed to compare a group of multiple cases against a group of multiple controls. Because we have no class labels, our data consist of two single synthetic methylomes (min, max) and therefore of case and control groups of one sample each. The main formula of the BSmooth algorithm

$$t\left( c \right) = \frac{\Delta \left( c \right)}{{\left[ {\sigma \left( c \right)\sqrt {\frac{1}{{n_{2} }} + \frac{1}{{n_{2} }}} } \right]}}$$

calculates a signal-to-noise statistic t(*c*) for each CpG *c* with Δ(*c*) referring to the mean methylation differences of both groups.

In our case, we reduced this formula to *t*(*c*) := (max(*c*) − min(*c*)) for each CpG *c*. The terms max(*c*) and min(*c*) simulate the process of creating synthetic methylomes by selecting the maximum and minimum methylation level of a CpG over all samples. DMRs are formed by consecutive groups of CpGs with *t*(*c*) > *v* or *t*(*c*) < −*v* with a threshold *v* > 0. We use *v* = 0.5 as parameter for the DMR calling.

A DMR’s border may differ in shape, and DMR calling algorithms often cannot identify them exactly. In contrast to BSmooth, we calculate a DMR’s methylation level by building a weighted average methylation level

$$\mu_{i} \left( d \right) = \frac{1}{{\left| {C\left( d \right)} \right|}}\mathop \sum \limits_{c \in C\left( d \right)} \sigma \left( c \right)m_{i} \left( c \right)$$

for DMR *d*, its set of CpGs *C*(d), the methylation levels *m*_*i*_(*c*) and standard deviation over all samples *σ*(*c*) of CpG *c* ∈ C(*d*) in sample *i*. We call *μ*_*i*_(*d*) the core methylation of *d* in sample *i*. The core methylation is less influenced by the inaccurate DMRs borders. We only keep DMRs with high (core) mean methylation differences ≥0.8 and sufficiently long DMRs consisting of 4 CpGs or more.

To test the DMRs for statistical significance, we calculated an empirical *p* value by simulating 1000 sets of five samples according to the null model that there is no methylation difference as follows:

Let *n*_*s,c*_ be the coverage and *m*_*s,c*_ the methylation count for observed sample *s* at CpG *c*. First, we calculate the average methylation

$$p_{c} = \frac{{\sum m_{s,c} }}{{\sum n_{s,c} }}$$

value for each CpG *c.*

For each of the five observed samples *s*, we simulate a corresponding null sample *o*.

We set the coverage of CpG *c* in sample *o* to *n*_*o,c*_ = *n*_*s,c*_.

The methylation count *M*_o*,c*_ for each *c* is randomly chosen with binomial probability

$$P\left( {M_{o,c} = m} \right) = \left( {\begin{array}{*{20}c} {n_{o,c} } \\ m \\ \end{array} } \right) \cdot p_{c}^{m} \cdot \left( {1 - p_{c} } \right)^{{n_{o,c} - m}}$$

Therefore, the coverage of each CpG in the null sample is equal to the coverage in the corresponding observed sample, while differences in methylation are only caused by finite sampling size. Per definition, there exist no DMRs for the null samples, and every detected DMR is a false positive. We applied our algorithm for DMR detection to the null samples. We repeated the process 1000 times.

The algorithm did not detect any DMRs. This leads to an empirical *p* value <0.001 for each DMR.

### Calculation of the DMR detection rate

We assume that a single SNP is responsible for the methylation of a DMR and that the probability of being a causative SNP is independent of its allele frequency. We further assume that the epigenotypes follow the Hardy–Weinberg equilibrium with *P*(AA) = *p* ^2^, *P*(aa) = *q* ^2^ and *P*(Aa or aA) = 2*pq* with some frequencies *p* and *q* such that *p* + *q* = 1. Our approach is only able to detect DMRs where at least one out of *n* samples is fully methylated and at least one sample is unmethylated.

The probability of obtaining such an event can be derived from an urn model with three different types of balls. Two types with probabilities *p* ^2^ and *q* ^2^ = (1 − *p*)^2^, respectively, represent the two different homozygous SNP states, and the third type with probability 2*pq* represents the heterozygous SNP state. For *n* samples, the probability to draw at least one ball of each of the first two types is *P*(*p, q*) = 1 − [(1 − *p* ^2^)^*n*^ + (1 − *q* ^2^)^*n*^ − (2*pq*)^*n*^], where we have applied the inclusion–exclusion principle to the complementary event.

We used the known allele frequencies of all SNPs with a minor allele frequency >0.05 contained in dbSNP [33] to estimate the fraction of detectable DMRs. For *n* = 5, we estimate that we can detect 23% of DMRs with a minor epigenetic allele frequency >0.05.

### SNP genotyping

For donors Hm01, Hm02, Hm03, Hm05 and M55900, 2.5 million SNPs were genotyped using Illumina’s Omni2.5Exome Bead Array. For donors Hm06 and Hm10, SNP genotypes were inferred from the targeted bisulfite sequencing data (see below), or by Sanger sequencing regions amplified with primers listed in Additional file 22.

### DMR SNP correlation score calculation

The mean methylation level of a sample in a region with allele-specific methylation is expected to be either close to 0.0, close to 1.0 or about 0.5. Due to inaccurate DMR borders, finite sequencing coverage and noise, measured values may differ from this expectation. We assume three possible classes “full-methylated,” “half-methylated” and “unmethylated” for this epigenotype.

In order to compare these epigenotypes with SNP genotypes, we have to classify the methylation level of each sample for each DMR. To avoid fixed thresholds for class assignment, we calculate the posterior probabilities of mean DMR methylation level to fall into each of the classes. We consider the empirical distribution (histogram) of 157 × 5 = 768 core methylation levels *μ*_*i*_(*d*) of each sample *i* and DMR *d*, which contains data from all three classes. This empirical distribution can be decomposed into a three-component mixture of beta distributions. A beta distribution is a continuous probability distribution on the unit interval [0, 1] that is frequently used to model data that naturally takes values between 0 and 1 [34] such as methylation levels. Each component beta distributions have two parameters *α* and *β* that determine the shape of the beta distribution. We used the betamix software [35] to robustly fit a three-component beta mixture model to the observed histogram.

Let *α*_*i*_ and *β*_*i*_ be the beta distribution parameters and *π*_*k*_ *t*he mixture coefficient of component *k* € {0, 1, 2} after fitting. For a single sample, a DMR with a core methylation level *μ* and one SNP genotype *g* € {0, 1, 2}, the posterior probability of *g* given *μ* is given by

$$L\left( {g,{{\mu }}} \right) = \frac{{\pi_{g} b_{{\alpha_{g} ,\beta_{g} }} \left( {{\mu }} \right)}}{{\mathop \sum \nolimits_{k} \pi_{k} b_{{\alpha_{k} ,\beta_{k} }} \left( {{\mu }} \right)}}.$$

To calculate a score based on multiple samples, we extend the formula. Let *g*_*i*_(*s*) € {0, 1, 2} be the genotype and *μ*_*i*_(*d*) be the core methylation level (see “Detecting DMRs” section) for DMR *d*, sample *i* and SNP *s*. The joint posterior probability

$${\text{score}}\left( {s,d} \right) = \mathop \prod \limits_{i} L\left( {g_{i} \left( s \right),\mu_{i} \left( d \right)} \right)$$

is given by the product of the single posterior probabilities over all samples. We use this posterior probability as a score to assess whether DMR *d* and SNP *s* are co-varying. The scores are separately calculated for each DMR and each SNP within a range of ±6 kb of the DMR’s location. For *n* = 5 samples, we used 0.9 as a threshold to call an SNP correlated with a DMR.

### GWAS analysis

The SNP array data were produced with three different SNP array types: Omni1_Quad_v1 (334 probands), OmniExpress_12v1.0 (627 probands) and OmniExpress_12v1.1 (170 probands). The data were normalized and CpG methylation levels extracted using RnBeads v1.2.2 [36].

We filtered each array separately by removing SNPs that failed the Hardy–Weinberg test at a significance threshold of 0.001, having a minor allele frequency less than 0.01 or a missing rate greater than 0.1 using plink v1.07 [37]. The arrays were merged by plink and the data again filtered by plink using the previously described parameters. This merged data served as genotypes for the GWASs.

The Spearman correlations and *p* value calculation between methylation levels and SNP genotypes were performed using NumPy v1.11.0 and SciPy v0.14.0 [38].

For the imputation, a region was chosen that includes all SNPs with a *p* value <5 × 10^−8^ but to a maximum of ±1 Mb of the CpGs position. The arrays were then converted to ped-format using gtool v0.7.5 [39] and separately imputed using impute2 [40] for the determined regions with the phase 3 data of the 1000 genomes project [41]. The imputed data were reconverted to bed-format again using gtool and merged under the previously given filter parameters by plink.

### DMR validation by targeted deep bisulfite sequencing

Bisulfite-converted DNA was obtained using 500 ng of monocytes DNA (Hm01, Hm02, Hm03, Hm05, Hm06 and Hm10) and the EZ DNA Methylation-Gold Kit (Zymo Research) according to the manufacturer’s instructions. Locus-specific bisulfite amplicon libraries were amplified by PCR employing bisulfite tagged primers (Additional file 22) designed using the MethPrimer [42] and BiSearch [43, 44] tools and HotStarTaq Master Mix (Qiagen). Sample-specific barcode sequences (MID, multiplex identifiers) and universal linker tags (454 adaptor sequences) were added by performing a second PCR. Samples were prepared and sequenced on a Roche/454 GS Junior system (Roche Diagnostics) with special filter settings applied to increase the yield of reads [45]. Automated CpG methylation analysis was performed using the Amplikyzer software [46] with minimum bisulfite conversion rate set to 95%, leading to an average of 2450 reads per sample (minimum 187).

### Histone modification ChIPseq heatmaps

Heatmaps visualizing the ChIP log2-ratio between signal and input across six histone modifications in two biological replicates were generated using deepTools [47] as previously described [10], except that we plotted data from 2-kb regions centered on the middle of 157 DMRs and clustered them using *k* = 5 clusters in the *k*-means algorithm. Independent clustering was performed for Hm03 and Hm05, since the two donors differ in the DMRs DNA methylation values.

### Chromatin segmentation by chromatin states

Chromatin segmentation of samples Hm03 and Hm05 was performed with ChromHMM [16]. We estimated the *p* value for over- and underrepresented chromatin states by simulating 1 million datasets, consisting of 151 regions each and equal size distribution compared to our DMRs. Each of the simulated regions was selected from non-repetitive regions covering at least 4 CpGs. For each of our 157 DMRs and each region of the simulated datasets, the overlap between its coordinates and the chromatin states of each sample (Hm03, Hm05) was calculated. We then compared the count of overlapping chromatin states of a sample for our DMRs and each random set. The empirical *p* value for overrepresentation for state x is the fraction of random sets that have a higher count for x than the DMRs. The empirical *p* value for underrepresentation for state x is the fraction of sets that have a lower count for x than the DMRs. We partitioned the absolute methylation differences between Hm03 and Hm05 into (1) a set for the DMRs with different chromatin states in the two donors and (2) another set for the DMRs with the same states. We consider a state as different in the two donors, if the intersection of the DMR overlapping chromatin states is empty. The two sets of methylation differences were then compared by applying the Wilcoxon rank-sum test to determine if the methylation differences are independent from a chromatin state difference.

### GREAT analysis

The bioinformatic tool GREAT was used to predict DMR functions by analyzing the annotations of nearby genes [48], under species assembly GRCh37 with whole genome background and choosing the “Basal plus extension” association rule setting with default parameters of 5.0 kb upstream, 1.0 kb downstream and up to 1000.0 kb distal.

### Distribution of gene expression levels

To obtain the gene expression rates for each gene, we summed the transcript per million (tpm) values as calculated by kallisto with default parameters [49]. Since GREAT uses only the extremely high-confidence genes prediction subset of the UCSC Known Genes, we reduced the kallisto gene list to this subset (*n* = 17,784). We then partitioned the mean expression rates of Hm03 and Hm05 for each gene into two groups: genes that are associated with a DMR as identified by GREAT (*n* = 240) and genes that are not associated (17,544). We compared the expression rates of these two groups by applying a Wilcoxon rank-sum test to test for differences.

### Identification of transcription factor-binding motifs

We used the TRANSFAC database (professional version, release 2015.3, [50]) to determine, if certain motifs were enriched in the SNP regions (501 regions: SNP ± 100 bp). Example regions as provided by TRANSFAC served as background, and the parameters were set to default.

### Differential transcript expression

We ran Tophat 2.0.11 [51], with Bowtie 2.2.1 [52] and NCBI build 37.1 using the following parameters: –library-type fr-firststrand and –b2-very-sensitive setting, to generate the mapping files from total-RNA of Hm03 and Hm05 samples. Subsequently, StringTie [53] with NCBI build 37.1 was run in -e -b -G mode to generate files for analysis with Ballgown [54]. Differential transcript expression analysis was performed using Ballgown, and an FDR cutoff of 0.05 was chosen to extract the differentially expressed transcripts.

### Long non-coding RNAs

LNCipedia 4.0 [55, 56] (GRCh37/hg19) was used to extract high-confidence lncRNA regions. Bedtools was subsequently run in “intersect” mode to get the overlap of the differentially methylated regions with the lncRNA regions.

### Data retrieval

The full epigenome data from Hm03 and Hm05 monocytes (Study Accession ID: EGAS00001001595, Dataset Accession ID: EGAD00001002201) as well as the methylome data from M55900 (ENA PRJEB5800) and Hm01 (EGAS00001000719) have previously been produced by our group [10, 12]. The BLUEPRINT and CEEHRC WGBS datasets on human monocytes from other male donors were retrieved from the IHEC Data Portal (http://epigenomesportal.ca/ihec/grid.html; [57]). In addition, 450k array data of monocytes and whole blood DNA obtained from six individuals were downloaded from the gene expression omnibus (GSE35069) [20].

## Additional files

**Additional file 1.** Cluster analysis of Hm03 and Hm05 monocytes and macrophages of the 1000 most variable CpGs. CpG SNPs were excluded from the analysis. The difference between donors is greater than between cell types.

**Additional file 2.** Quality parameters of WGBS datasets.

**Additional file 3.** Principal component analysis (PCA) of ten monocyte methylomes from males generated by three IHEC consortia: DEEP (red, our datasets), BLUEPRINT (green) and CEEHRC (blue).

**Additional file 4.** Annotated list of DMRs including environment and GREAT target genes.

**Additional file 5.** Histogram of DMR sizes.

**Additional file 6.** Enrichment and depletion of DMRs for gene features.

**Additional file 7.** Correlation between differences in DNA methylation and histone modifications. Scatter plots showing, for each of the six histone marks, the difference in histone signals at the DMRs between Hm05 and Hm03 as a function of methylation differences between the two donors.

**Additional file 8.** Under- and overrepresentation of chromatin states.

**Additional file 9.** Methylation differences vs. changes in chromatin state. Distribution of DNA methylation differences between donors Hm03 and Hm05 in DMRs that have the same (left) or a different (right) chromatin state in both donors as determined by ChromHMM.

**Additional file 10.** DMRs target genes identified by GREAT. Number of DMR target genes (a) and their distance from the DMR (b).

**Additional file 11.** Expression levels of DMR related genes (240) vs. genes not associated with a DMR (17,544). *tpm* transcripts per million.

**Additional file 12.** List of DMRs with correlated SNPs in 12 kb window. SNPs within the same haplotype block are separated by a comma. Different haplotype blocks are separated by a slash.

**Additional file 13.** Validation of SNP correlations in seven DMRs using monocytes from six independent donor samples. Graphs showing relationship between the methylation levels as quantified by targeted deep bisulfite sequencing and the genotype of nearby SNPs. Hm01, Hm02, Hm03, Hm05, Hm06 and Hm10: donors.

**Additional file 14.** Amplikyzer comparative methylation plots. Plots show CpG methylation averages for 14 DMRs after sorting reads by allele of the correlated SNPs (16 SNPs). Each plot shows data from 1 to 4 independent donor samples heterozygous for the correlating SNPs. The two alternative alleles are defined with respect to the forward strand. SNPs rs1996180, rs13130981 and rs7925175 are A/C SNPs, but the C is converted to a T after bisulfite conversion. Asterisks mark CpGs that are outside the DMR borders.

**Additional file 15.** Histograms of 450k methylation levels in the 1128 probands.

**Additional file 16.** Scatter plots of monocyte vs. whole blood correlation of DNA methylation. Plots show the correlation between monocyte and whole blood methylation levels in six healthy male individuals for the 50 CpGs that are included in the Illumina 450k array. Analysis performed with Illumina 450k array data previously published [20].

**Additional file 17.** Manhattan plots of GWASs. *Dashed vertical line* DMR position. *Horizontal line* GWAS significance threshold.

**Additional file 18.** Zoom-ins with imputed SNPs. *Blue* lead-SNP. *Dashed vertical line* DMR position. *Horizontal line* GWAS significance threshold.

**Additional file 19.** HaploReg annotations of the 30 lead-SNP and SNPs in the corresponding haplotype blocks. Haplotype block: SNPs in high linkage disequilibrium, *r* ^2^ > 0.8. SNP positions were converted to hg19 coordinates.

**Additional file 20.** Distance of SNPs with known binding proteins to the corresponding DMR border. Number of known proteins binding to lead-SNPs or to SNPs in high LD with the lead-SNPs vs. their distance to the corresponding DMR border. Data from Encode ChIPseq obtained via the HaploReg database.

**Additional file 21.** TRANSFAC motif enrichment in 501 SNP regions (SNP ± 100 bp). Yes and No denote the relative number of sites for the selected matrix in the DMRs as compared to the background dataset.

**Additional file 22.** Primer sequences and PCR conditions for targeted bisulfite sequencing and genotyping 13 SNPs.

## Abbreviations

ASM: allele-specific methylation
CGI: CpG island
ChIP: chromatin immunoprecipitation
DMR: differentially methylated region
eQTL: expression quantitative trait loci
GO: gene ontology
GREAT: Genomic Regions Enrichment of Annotations Tool
GWAS: genome-wide association study
H3K27ac: histone H3 lysine 27 acetylation
H3K27me3: histone H3 lysine 27 tri-methylation
H3K36me3: histone H3 lysine 36 tri-methylation
H3K4me1: histone H3 lysine 4 mono-methylation
H3K4me3: histone H3 lysine 4 tri-methylation
H3K9me3: histone H3 lysine 9 tri-methylation
IHEC: International Human Epigenome Consortium
LD: linkage disequilibrium
mQTL: methylation quantitative trait loci
SNP: single nucleotide polymorphism
WGBS: whole genome bisulfite sequencing
