# Supplementary figures and images for: Regions of common inter-individual DNA methylation differences in human monocytes: genetic basis and potential function
Source: Epigenetics Chromatin. 2017 Jul 26;10:37. doi: 10.1186/s13072-017-0144-2 (PMC5530492; doi:10.1186/s13072-017-0144-2)

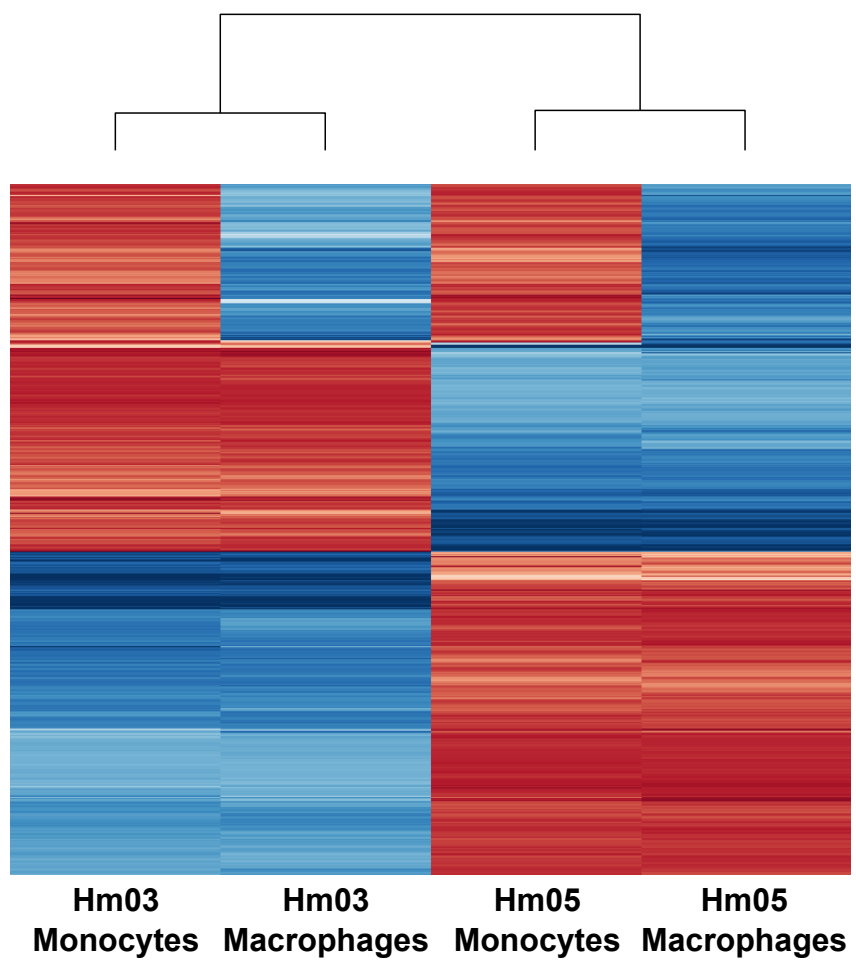

Supplement: Supplementary file 1 — Additional file 1. Cluster analysis of Hm03 and Hm05 monocytes and macrophages of the 1000 most variable CpGs. CpG SNPs were excluded from the analysis. The difference between donors is greater than between cell types. [file 13072_2017_144_MOESM1_ESM.pdf]

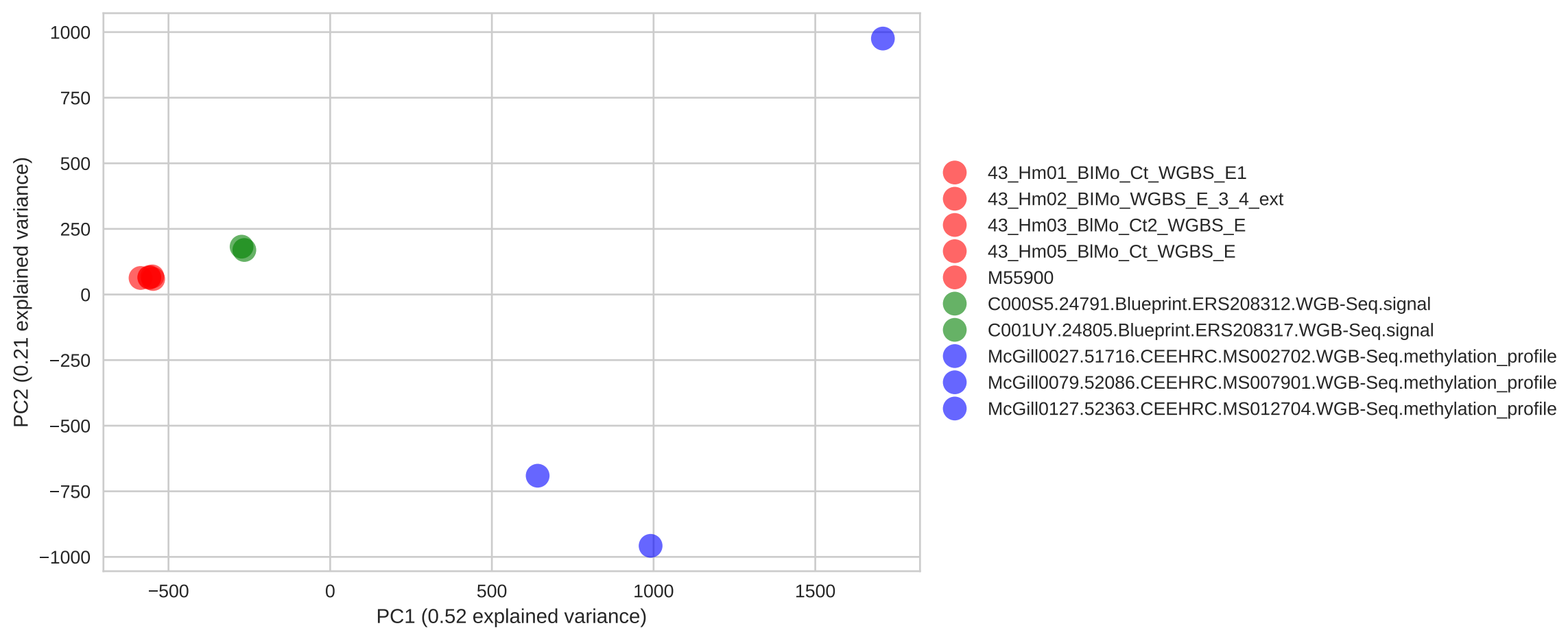

Supplement: Supplementary file 3 — Additional file 3. Principal component analysis (PCA) of ten monocyte methylomes from males generated by three IHEC consortia: DEEP (red, our datasets), BLUEPRINT (green) and CEEHRC (blue). [file 13072_2017_144_MOESM3_ESM.pdf]

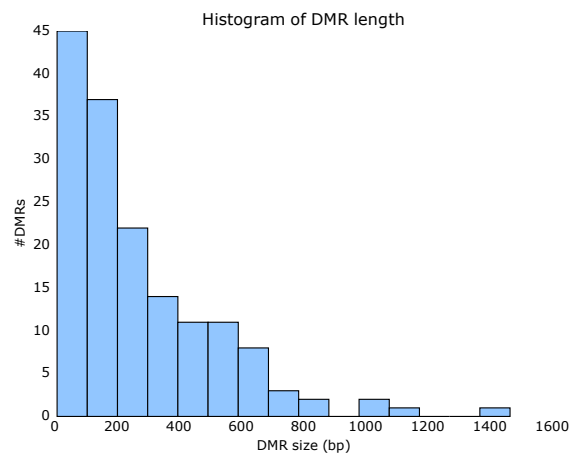

Supplement: Supplementary file 5 — Additional file 5. Histogram of DMR sizes. [file 13072_2017_144_MOESM5_ESM.pdf]

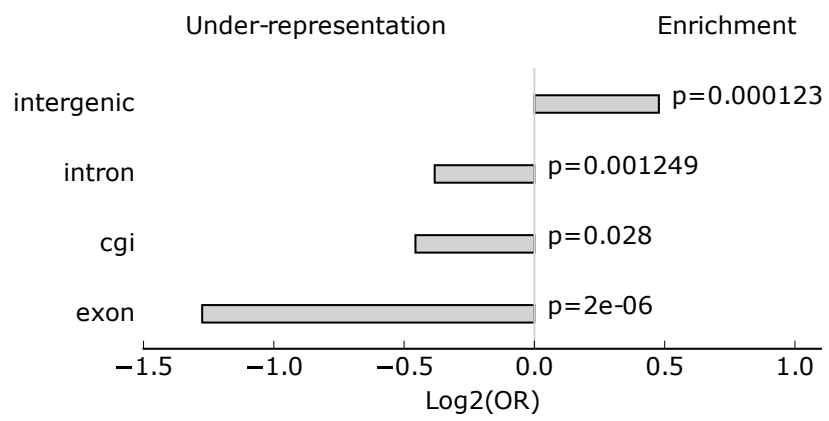

Supplement: Supplementary file 6 — Additional file 6. Enrichment and depletion of DMRs for gene features. [file 13072_2017_144_MOESM6_ESM.pdf]

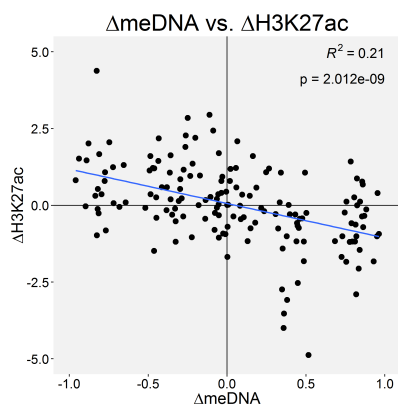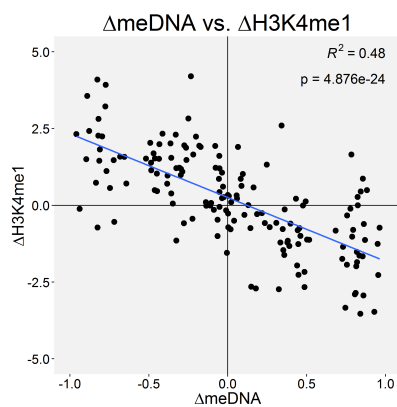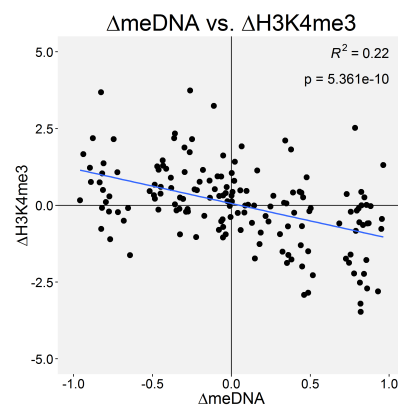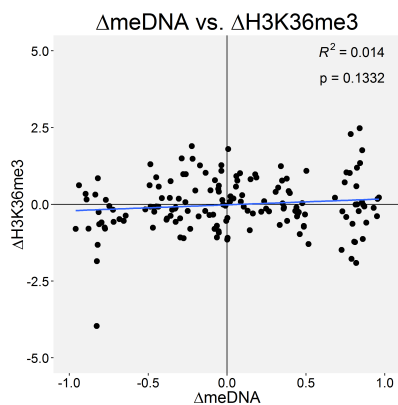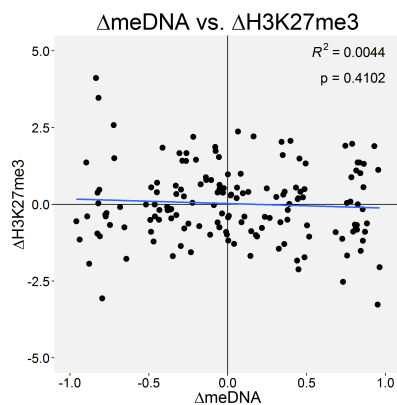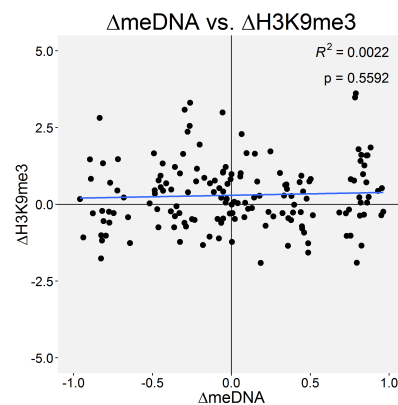

Supplement: Supplementary file 7 — Additional file 7. Correlation between differences in DNA methylation and histone modifications. Scatter plots showing, for each of the six histone marks, the difference in histone signals at the DMRs between Hm05 and Hm03 as a function of methylation differences between the two donors. [file 13072_2017_144_MOESM7_ESM.pdf]

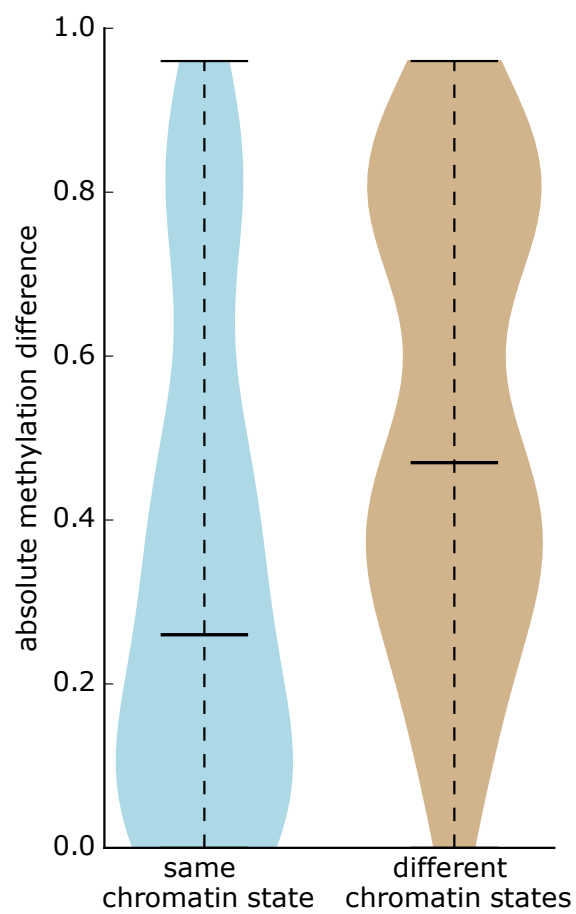

Supplement: Supplementary file 9 — Additional file 9. Methylation differences vs. changes in chromatin state. Distribution of DNA methylation differences between donors Hm03 and Hm05 in DMRs that have the same (left) or a different (right) chromatin state in both donors as determined by ChromHMM. [file 13072_2017_144_MOESM9_ESM.pdf]

**a**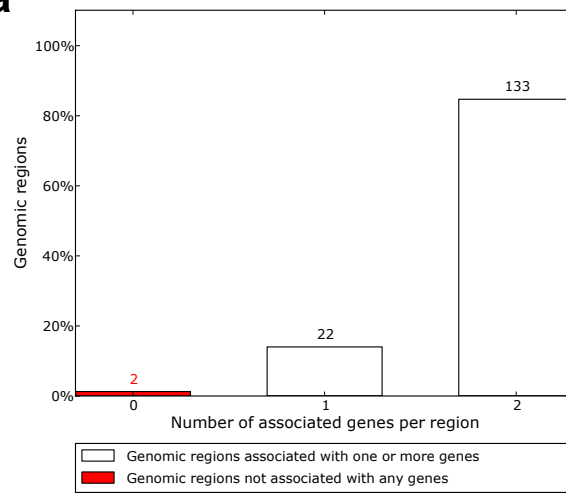**b**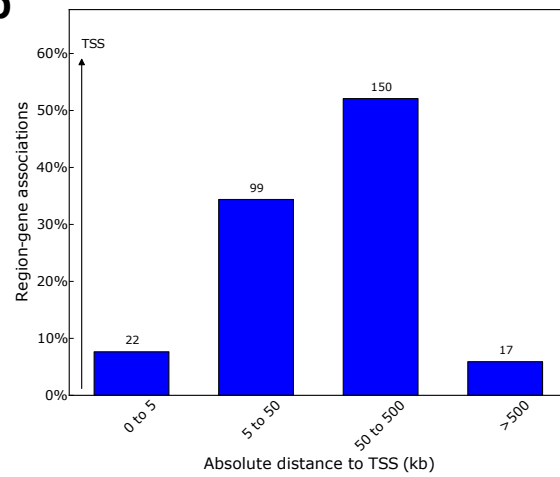

Supplement: Supplementary file 10 — Additional file 10. DMRs target genes identified by GREAT. Number of DMR target genes (a) and their distance from the DMR (b). [file 13072_2017_144_MOESM10_ESM.pdf]

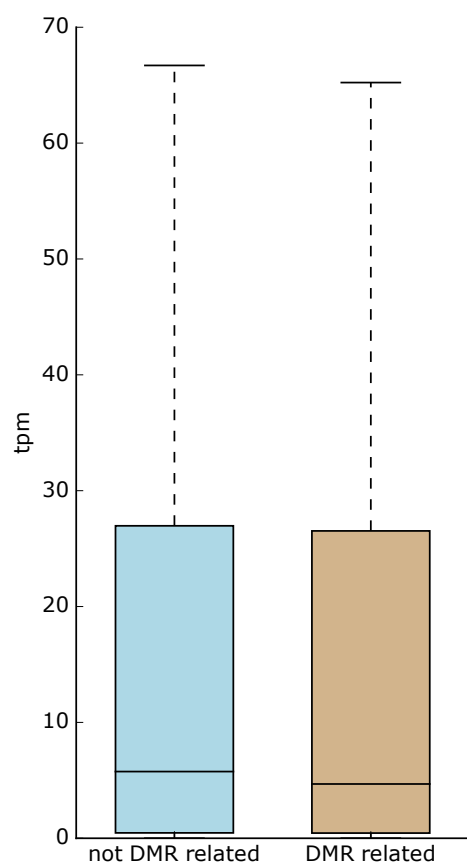

Supplement: Supplementary file 11 — Additional file 11. Expression levels of DMR related genes (240) vs. genes not associated with a DMR (17,544). tpm transcripts per million. [file 13072_2017_144_MOESM11_ESM.pdf]

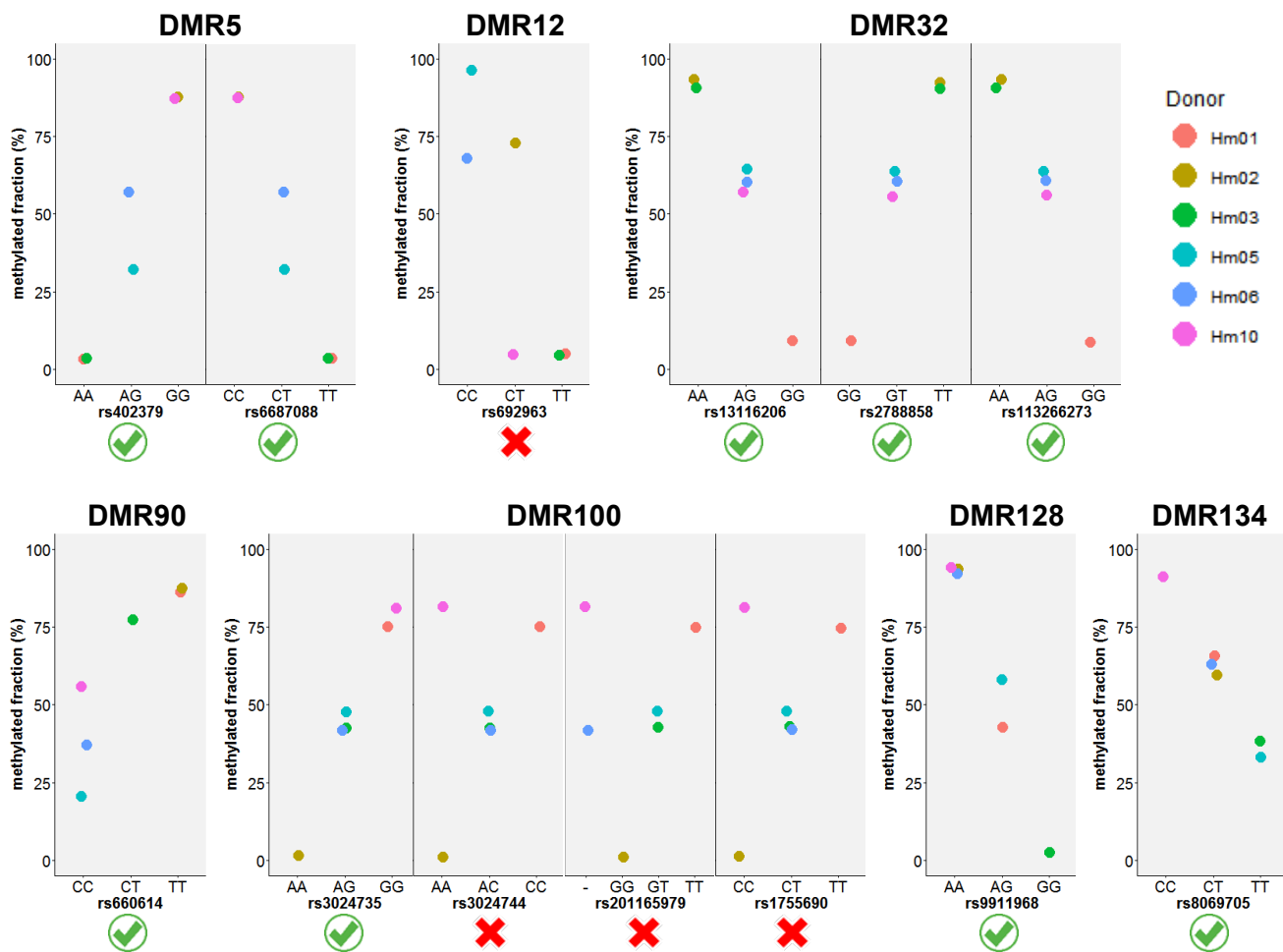

Supplement: Supplementary file 13 — Additional file 13. Validation of SNP correlations in seven DMRs using monocytes from six independent donor samples. Graphs showing relationship between the methylation levels as quantified by targeted deep bisulfite sequencing and the genotype of nearby SNPs. Hm01, Hm02, Hm03, Hm05, Hm06 and Hm10: donors. [file 13072_2017_144_MOESM13_ESM.pdf]

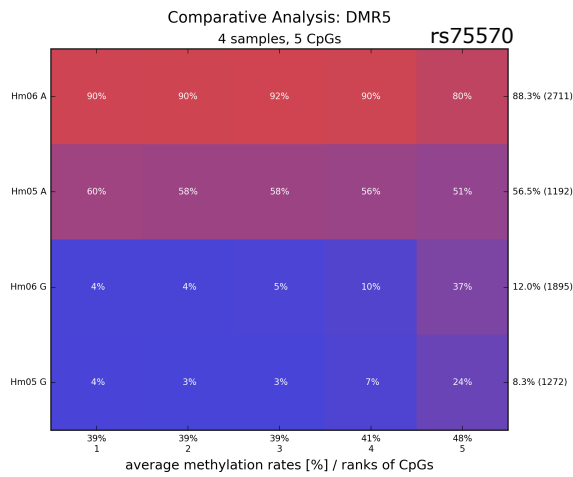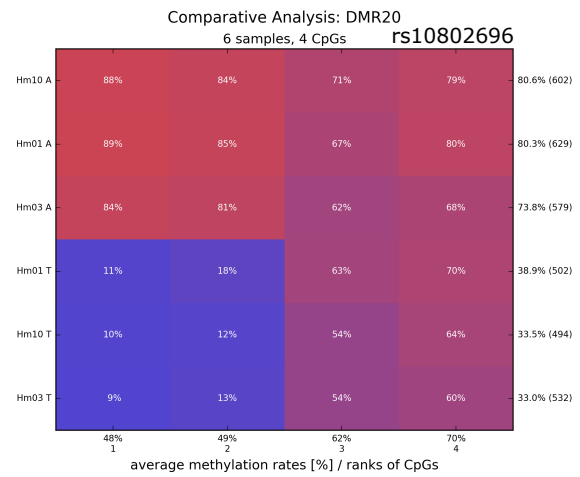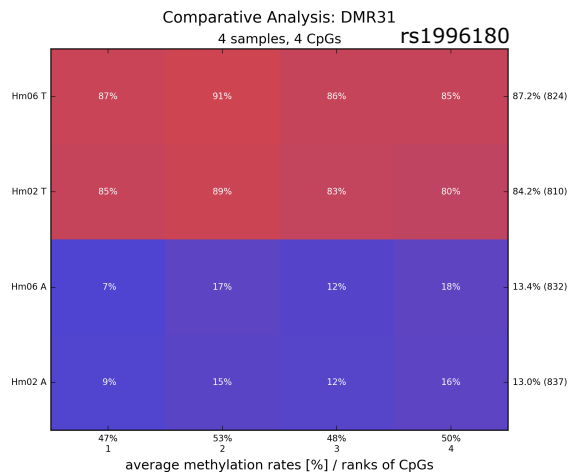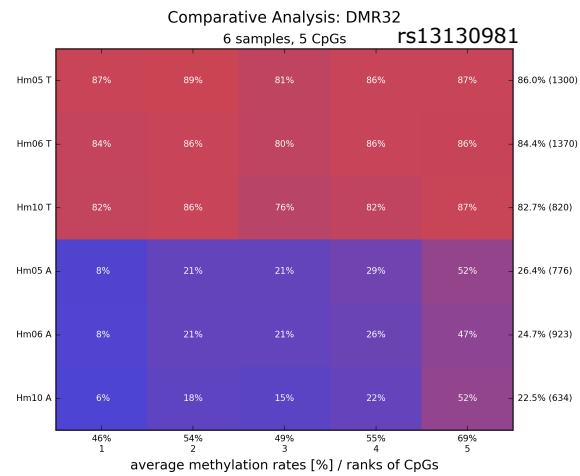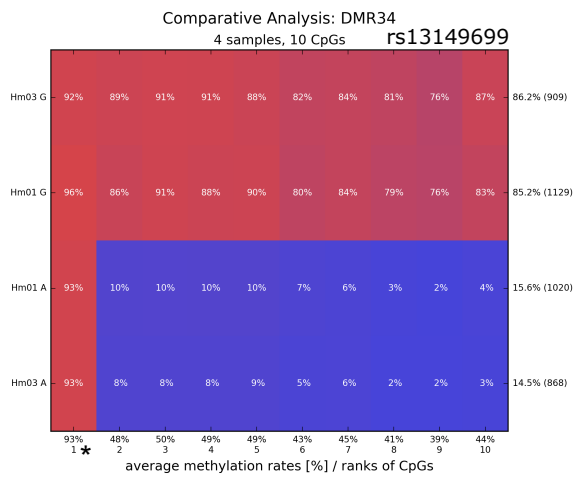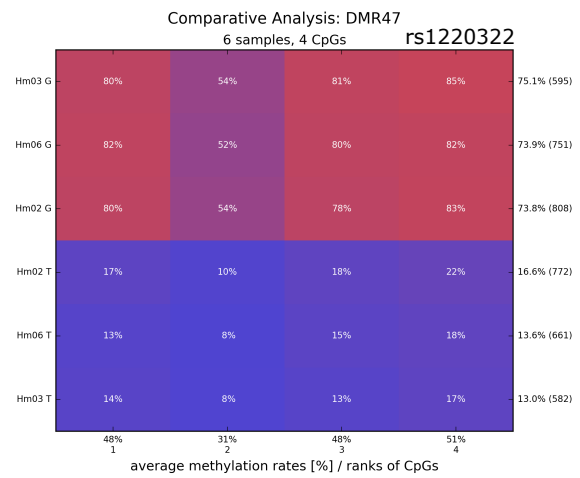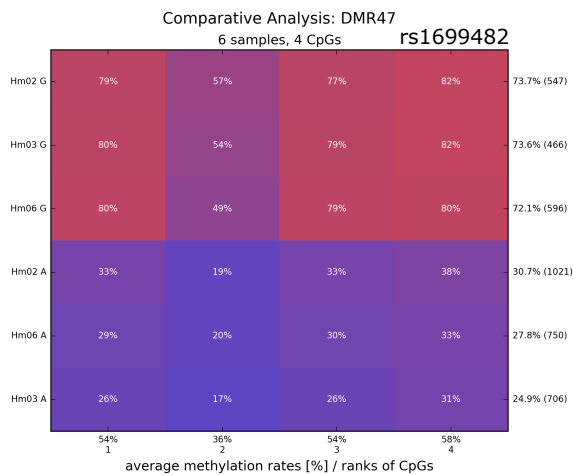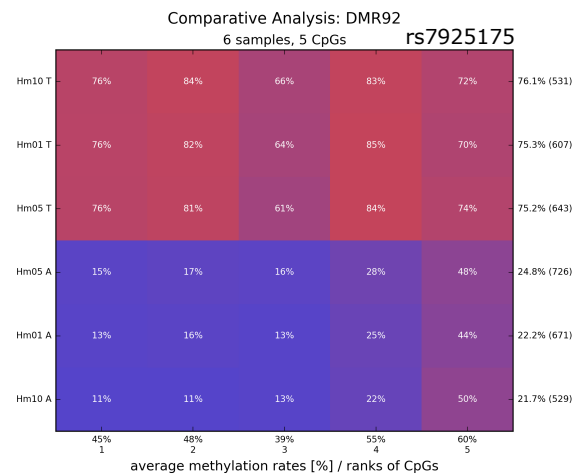

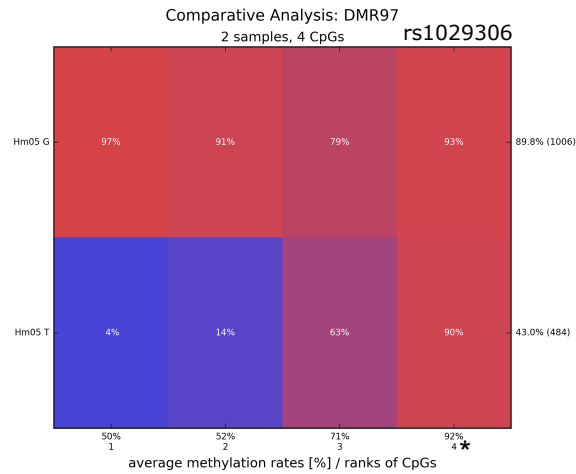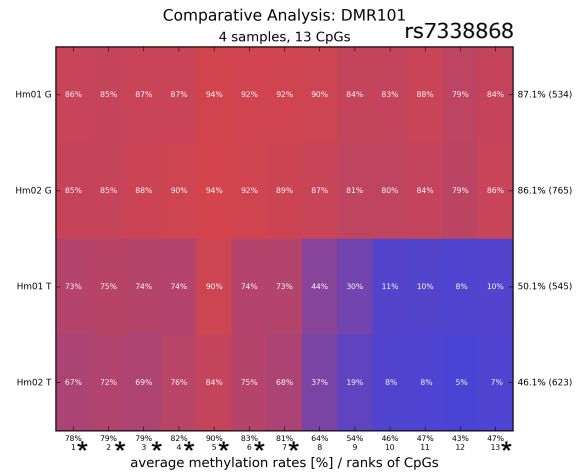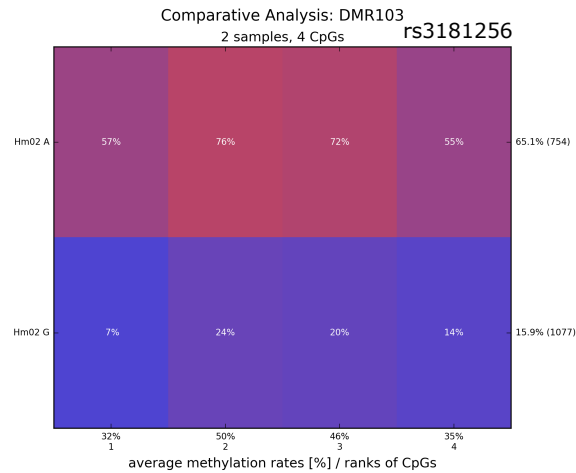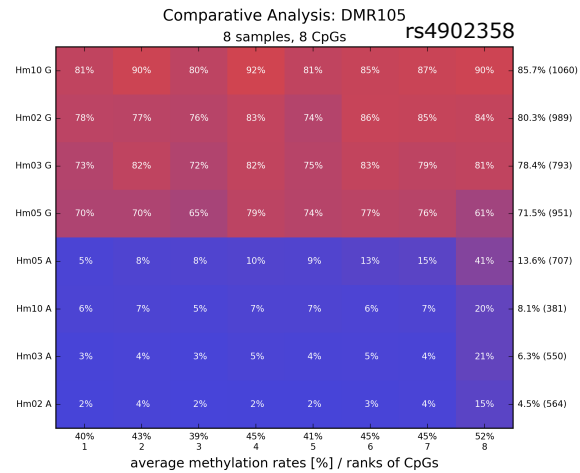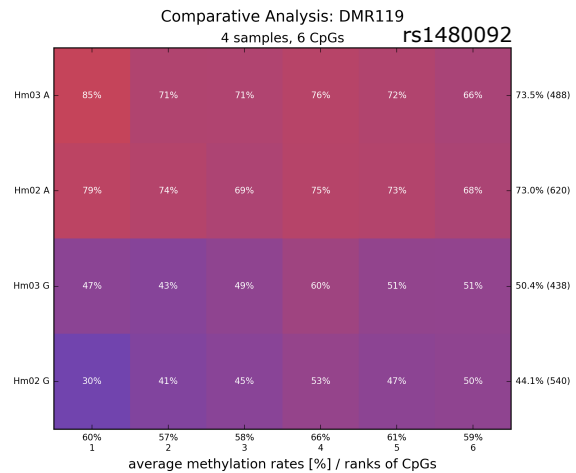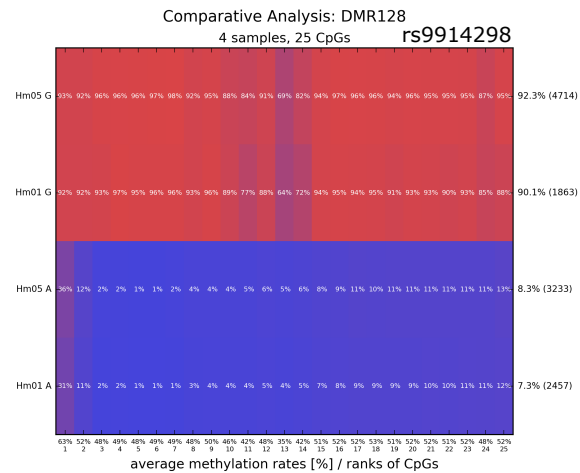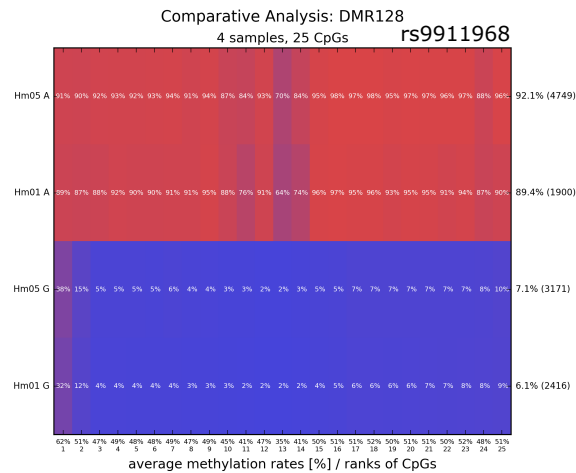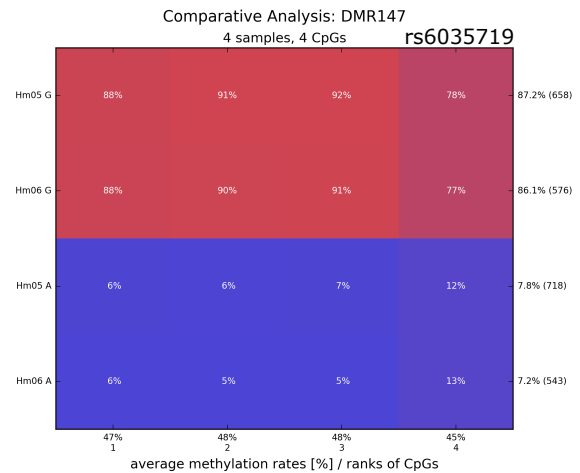

Supplement: Supplementary file 14 — Additional file 14. Amplikyzer comparative methylation plots. Plots show CpG methylation averages for 14 DMRs after sorting reads by allele of the correlated SNPs (16 SNPs). Each plot shows data from 1 to 4 independent donor samples heterozygous for the correlating SNPs. The two alternative alleles are defined with respect to the forward strand. SNPs rs1996180, rs13130981 and rs7925175 are A/C SNPs, but the C is converted to a T after bisulfite conversion. Asterisks mark CpGs that are outside the DMR borders. [file 13072_2017_144_MOESM14_ESM.pdf]

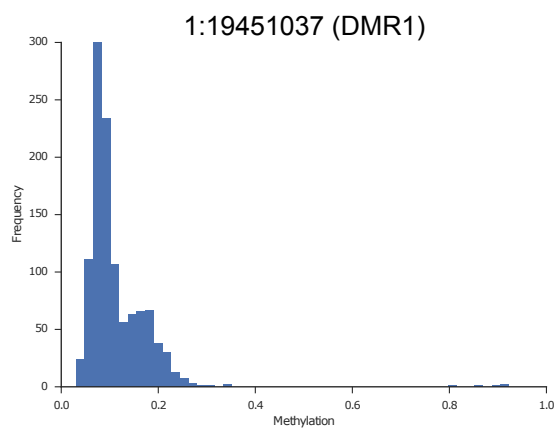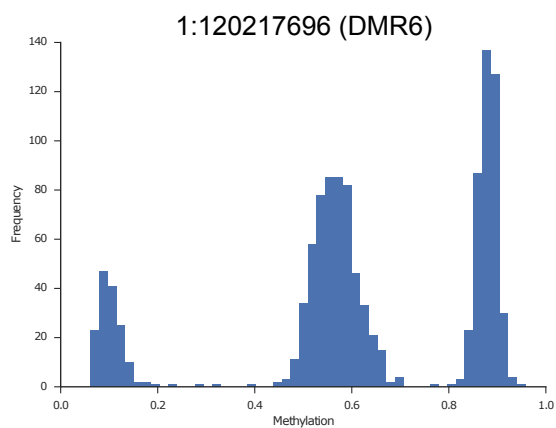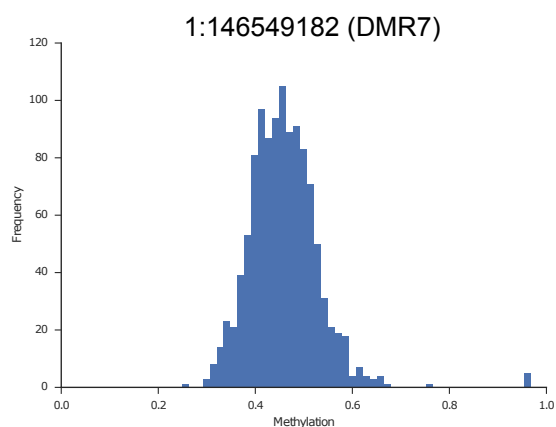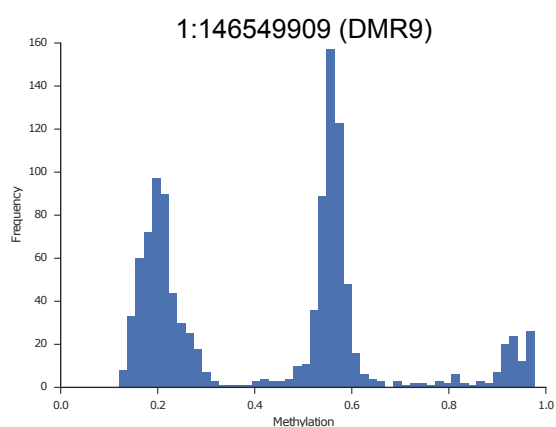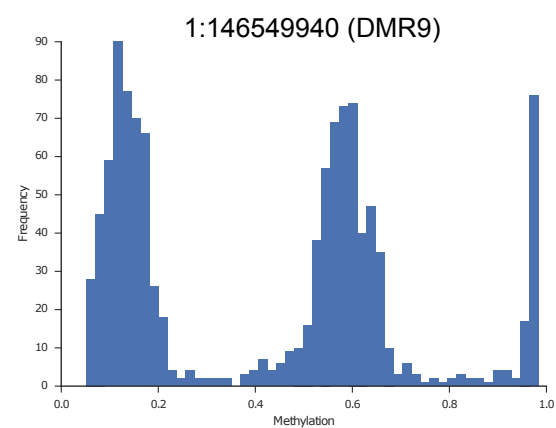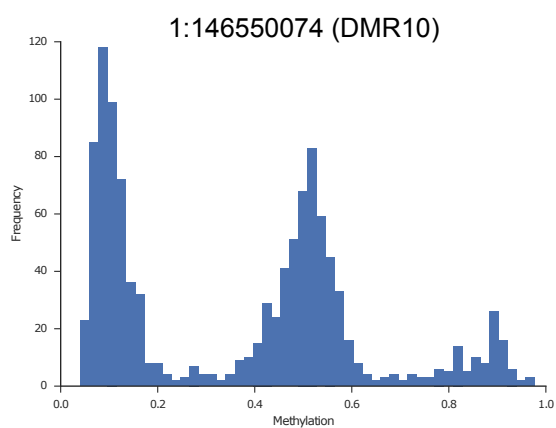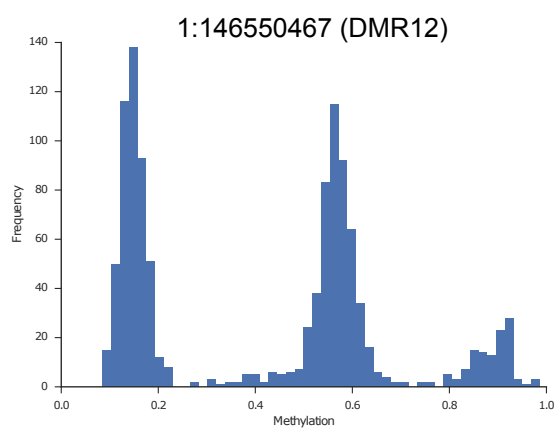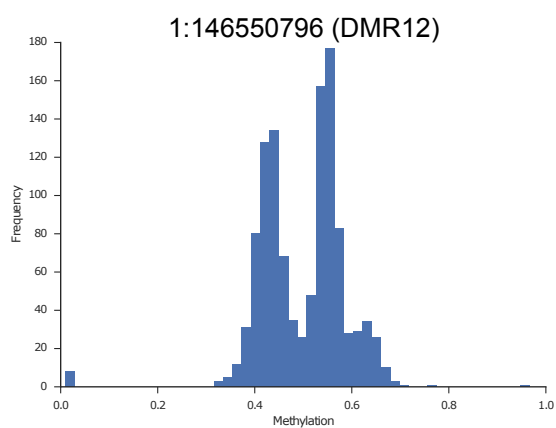

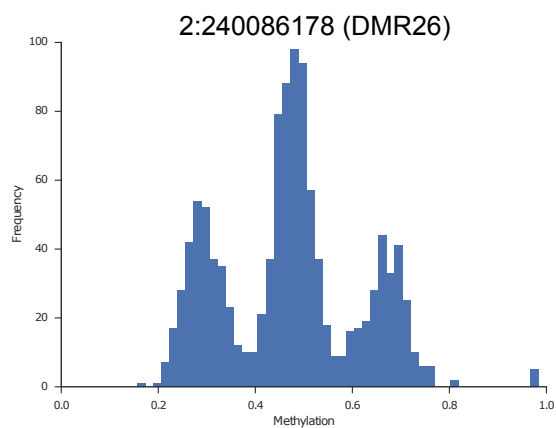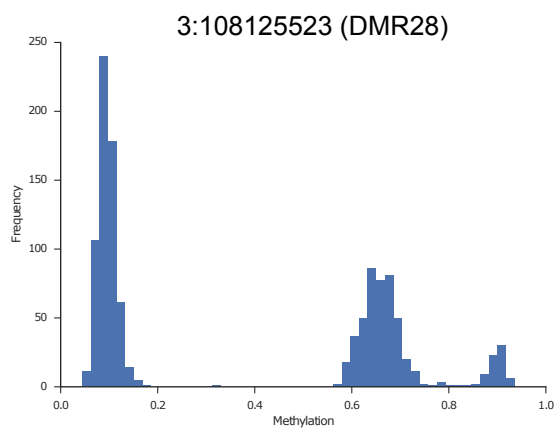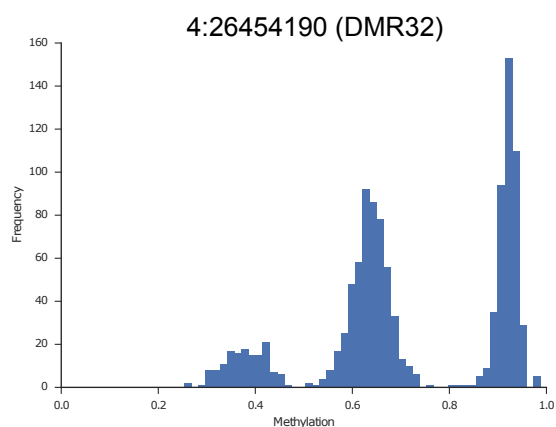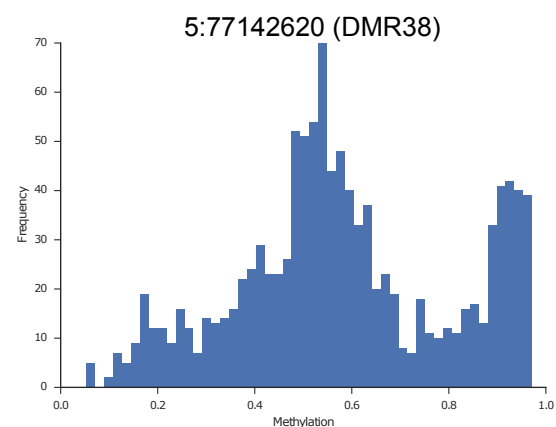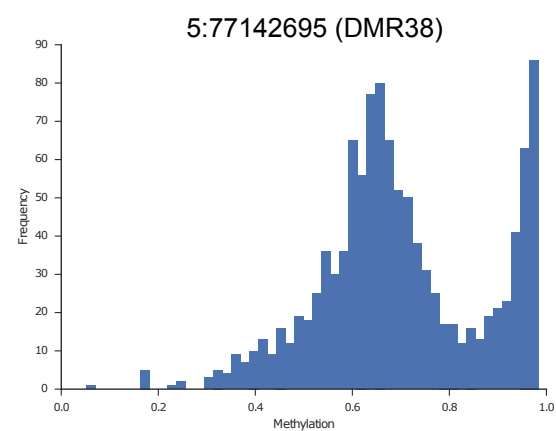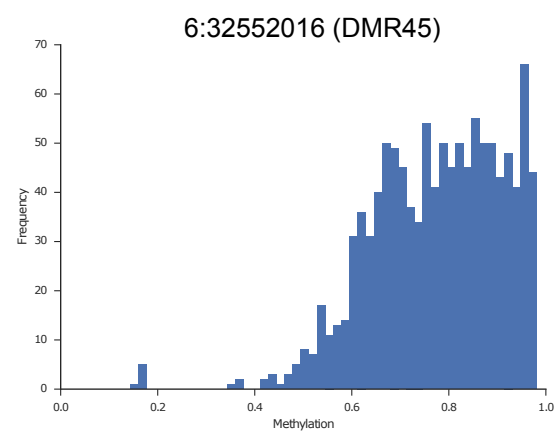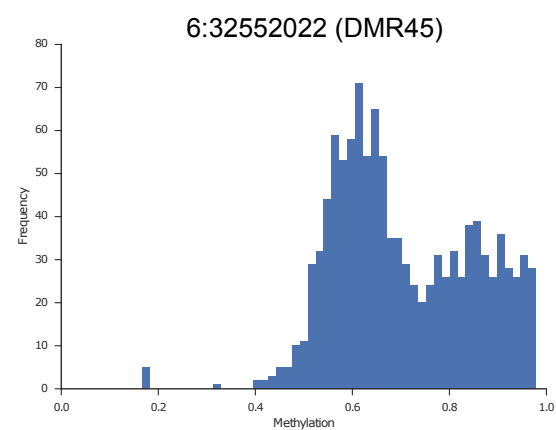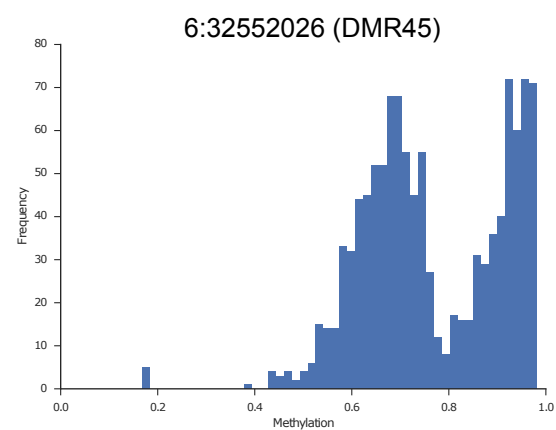

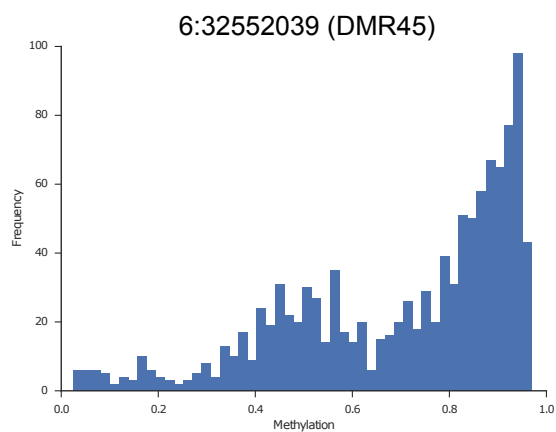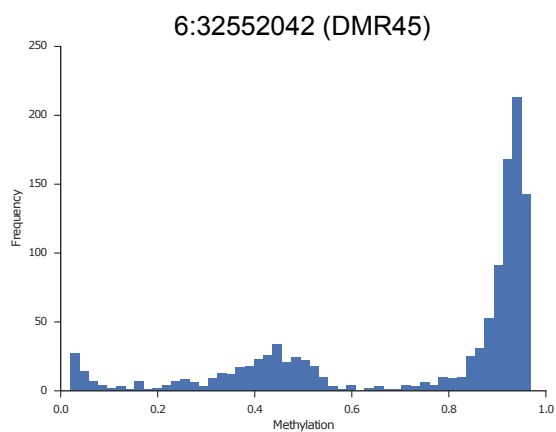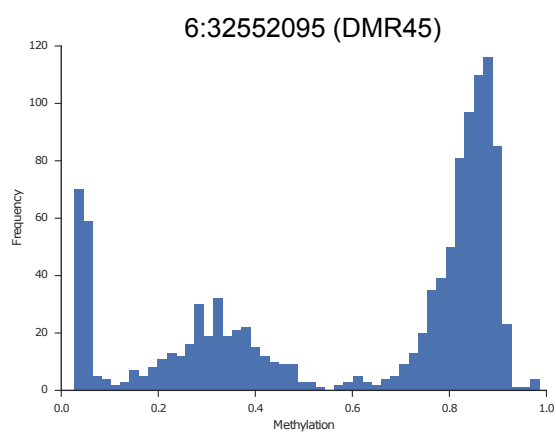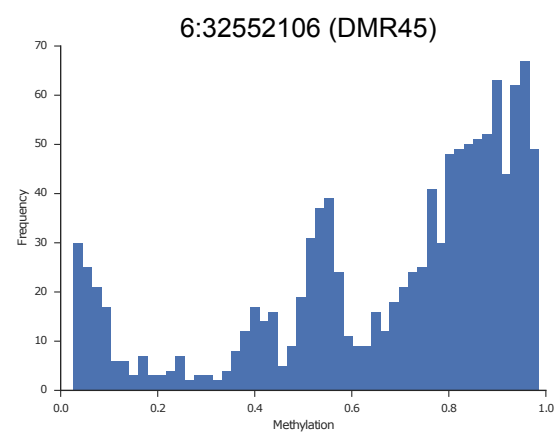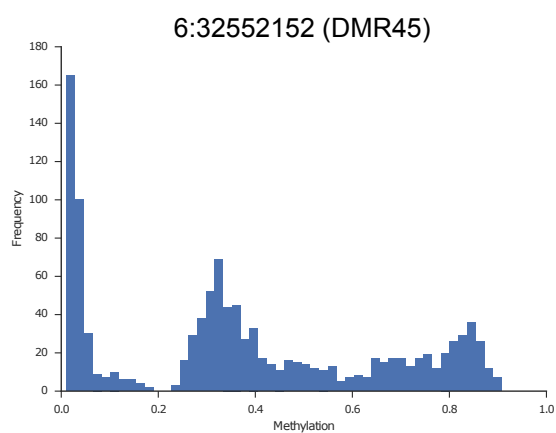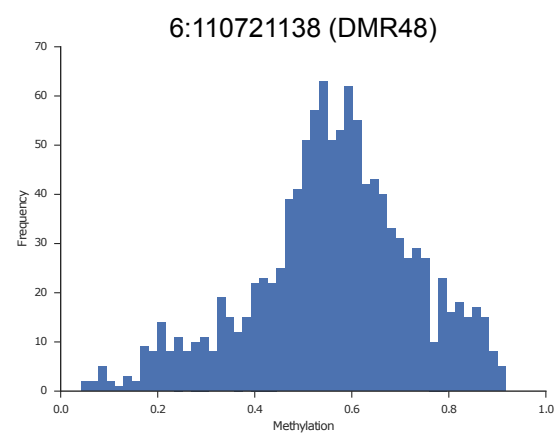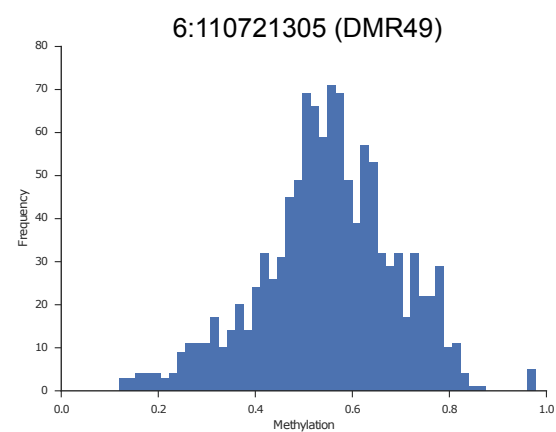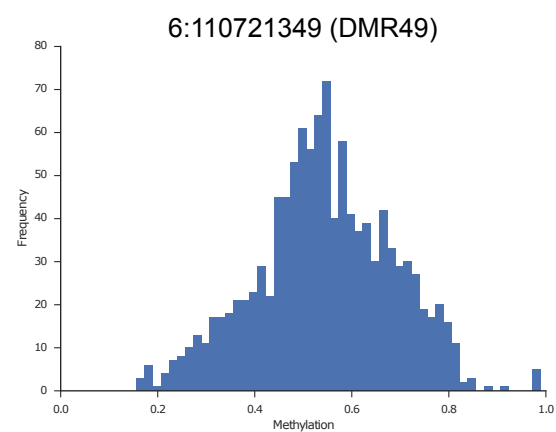

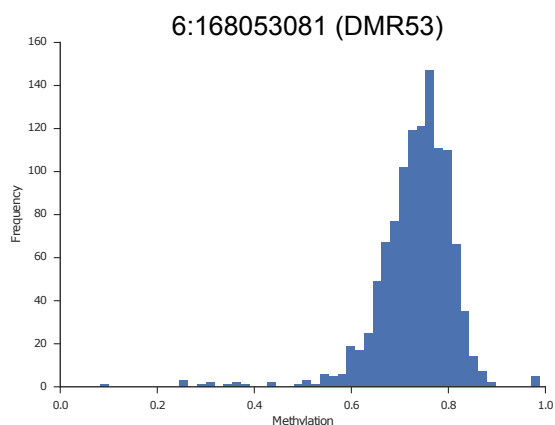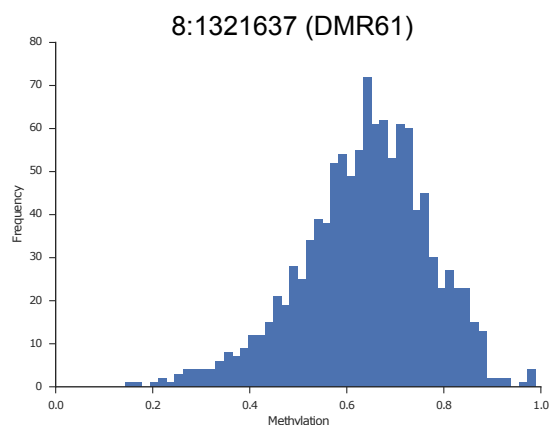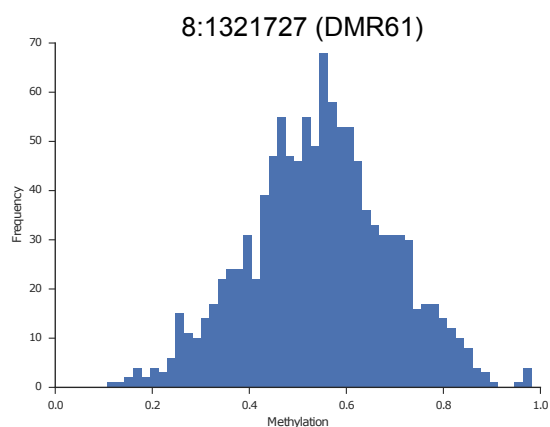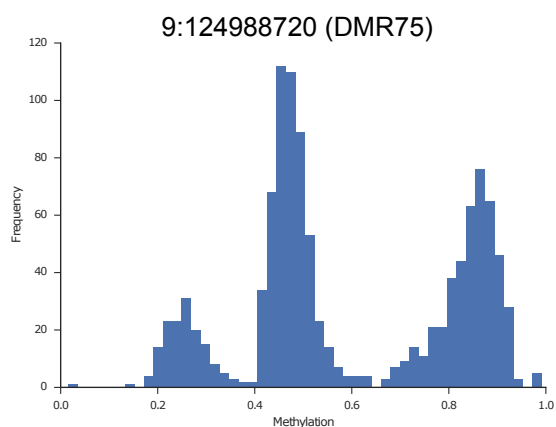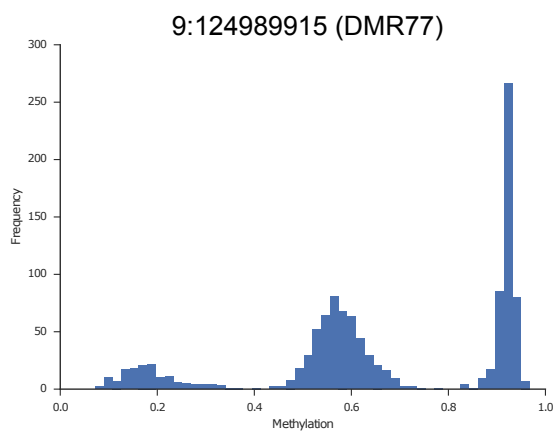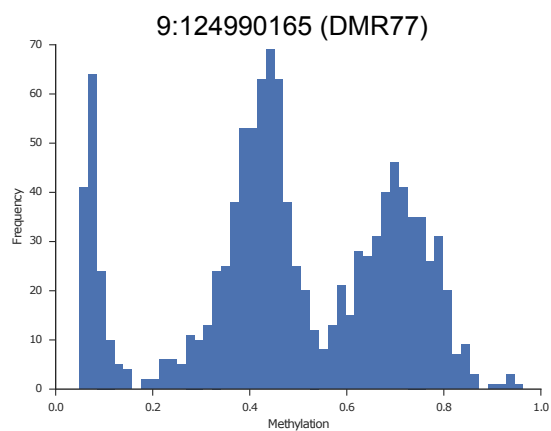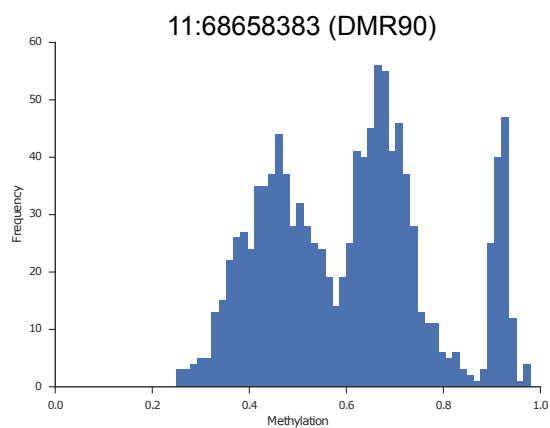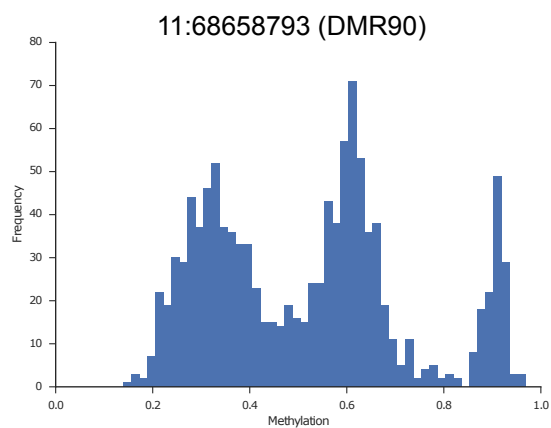

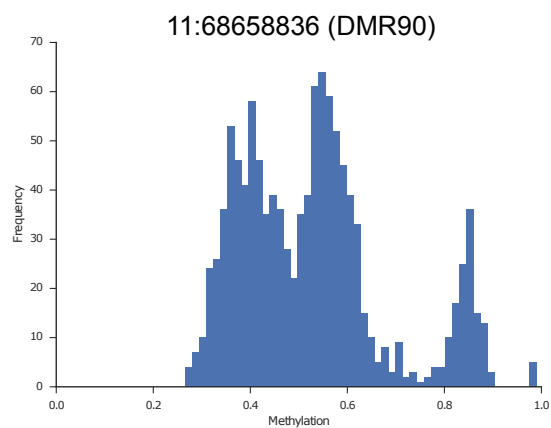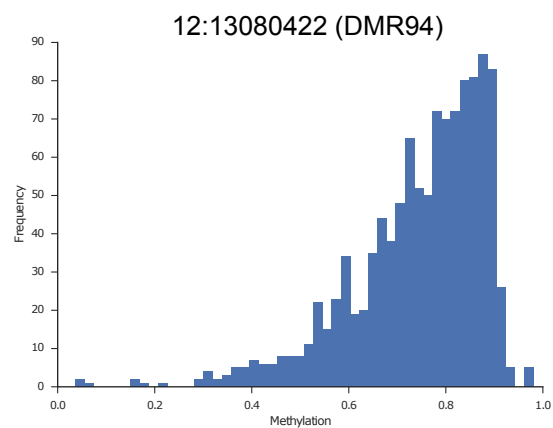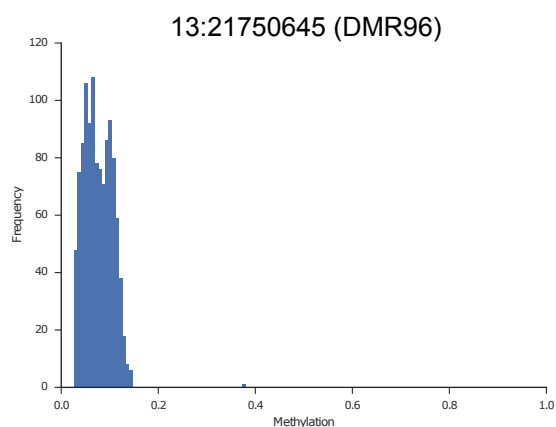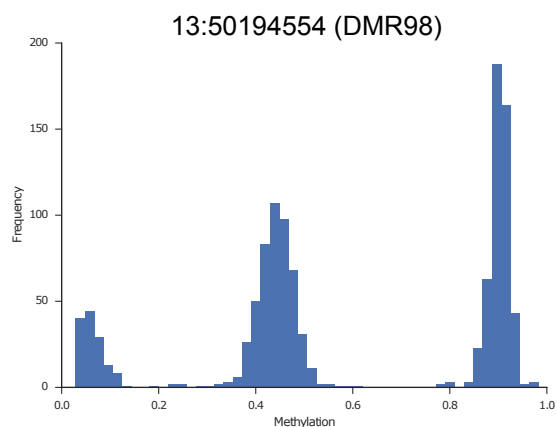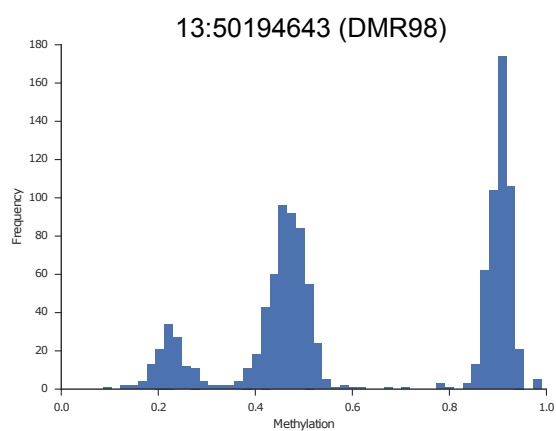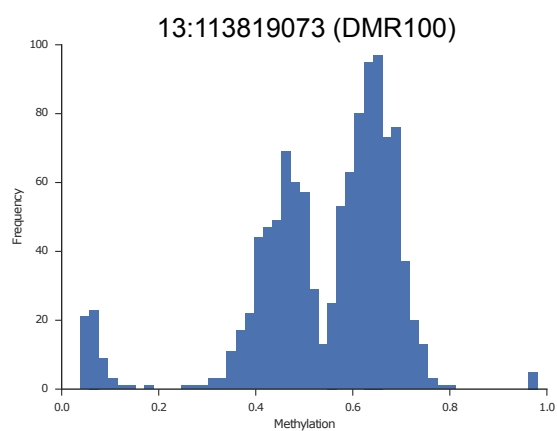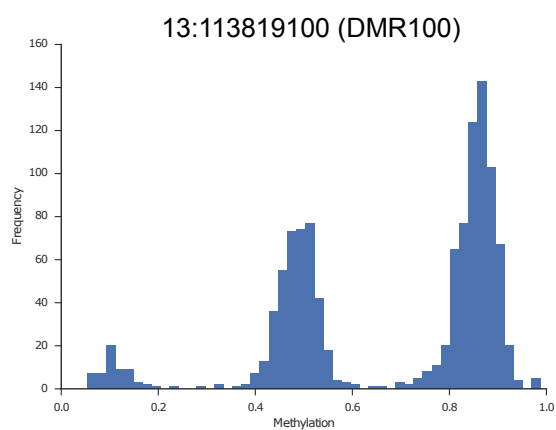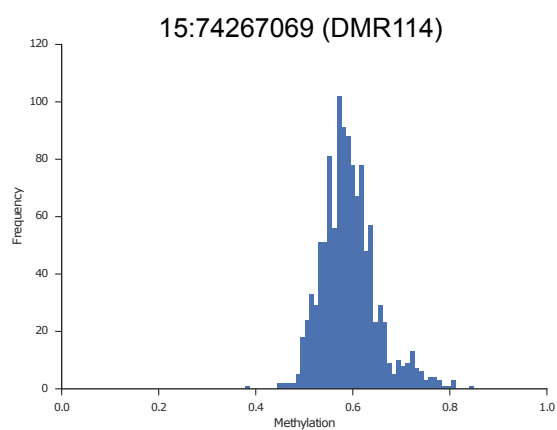

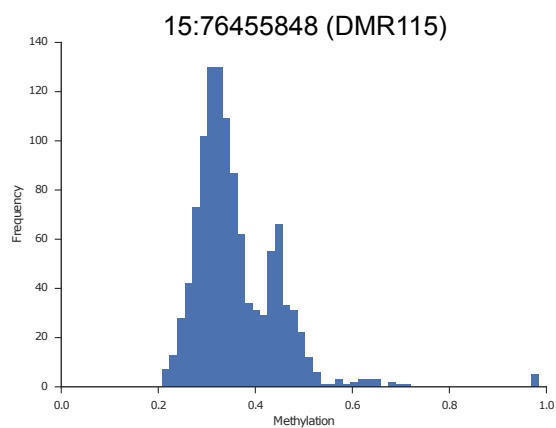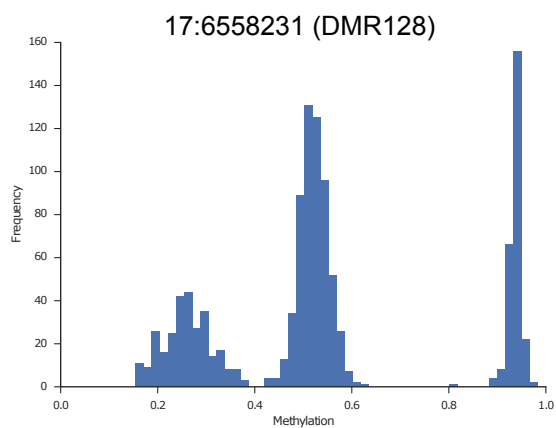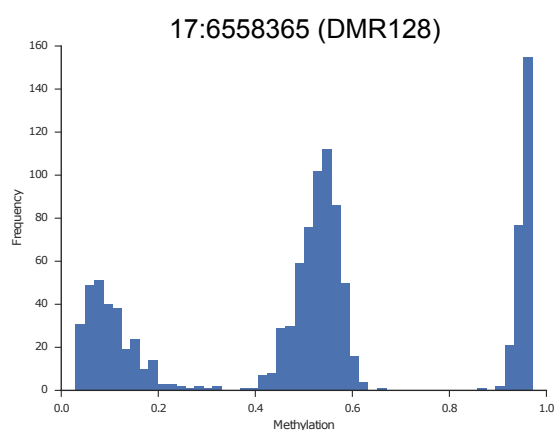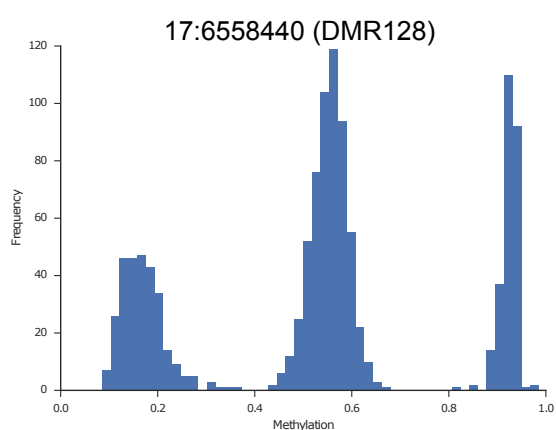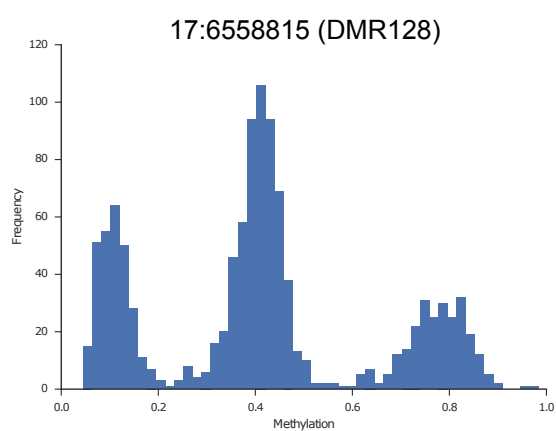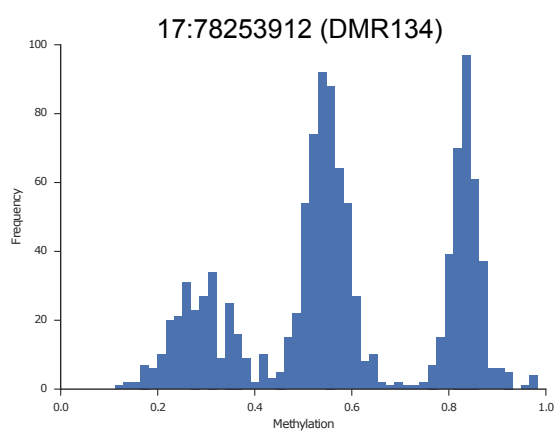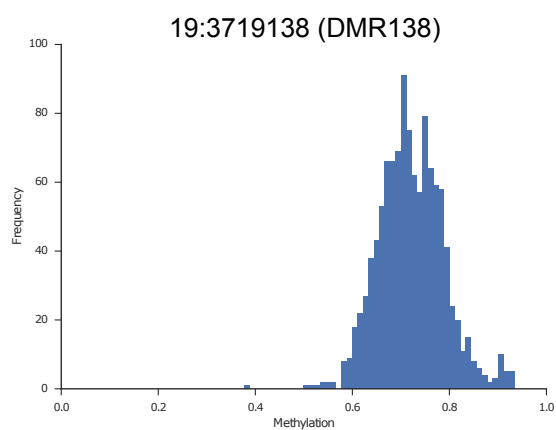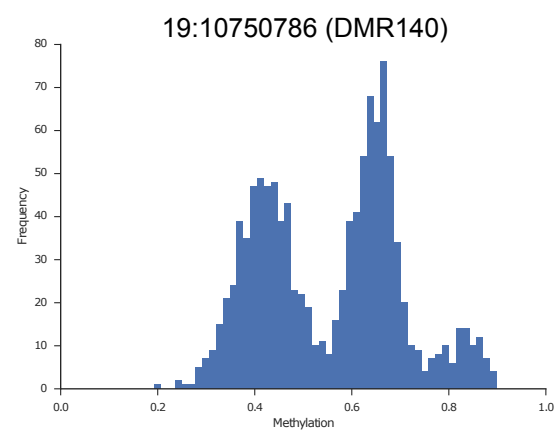

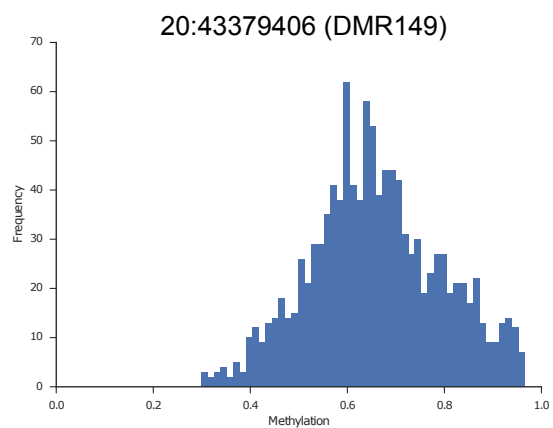

Supplement: Supplementary file 15 — Additional file 15. Histograms of 450k methylation levels in the 1128 probands. [file 13072_2017_144_MOESM15_ESM.pdf]

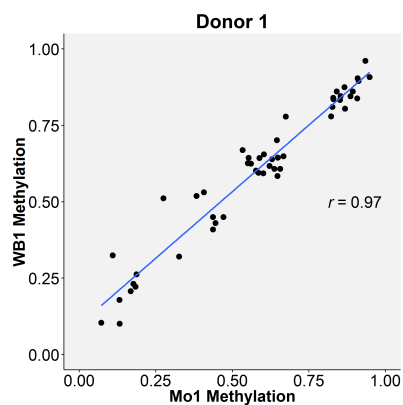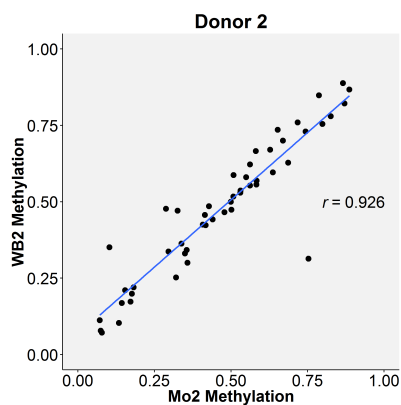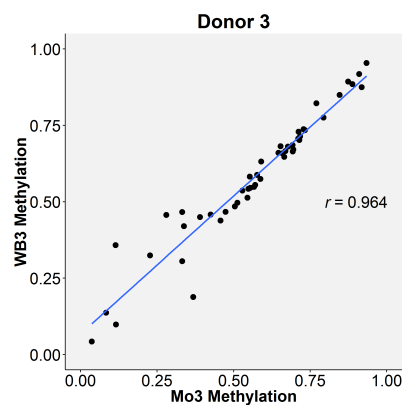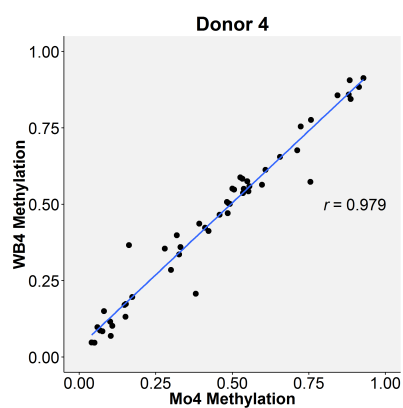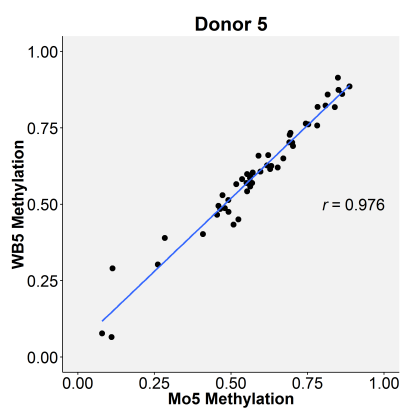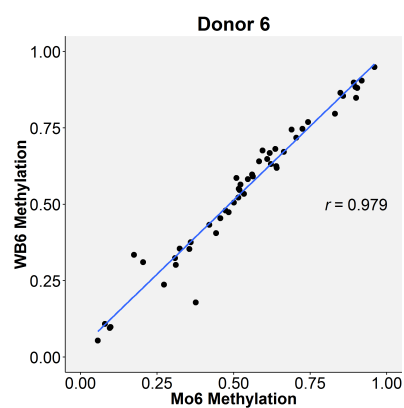

Supplement: Supplementary file 16 — Additional file 16. Scatter plots of monocyte vs. whole blood correlation of DNA methylation. Plots show the correlation between monocyte and whole blood methylation levels in six healthy male individuals for the 50 CpGs that are included in the Illumina 450k array. Analysis performed with Illumina 450k array data previously published [20]. [file 13072_2017_144_MOESM16_ESM.pdf]

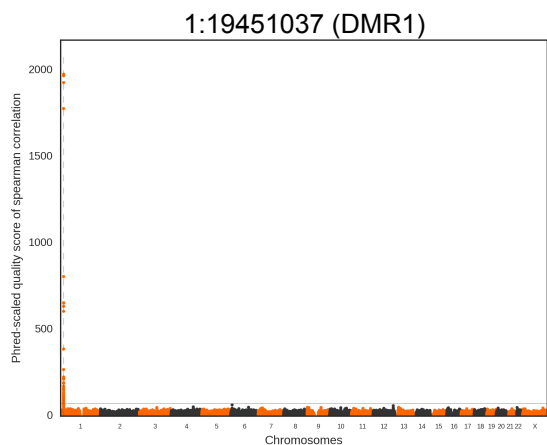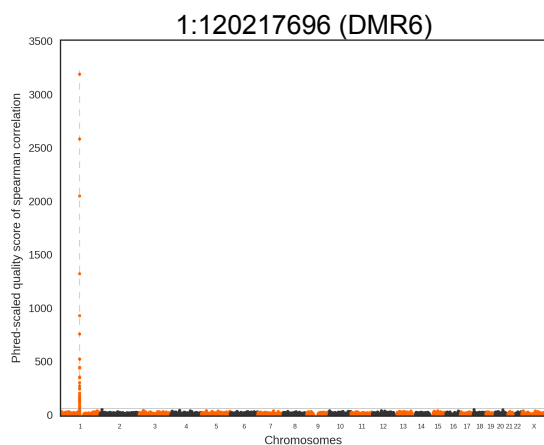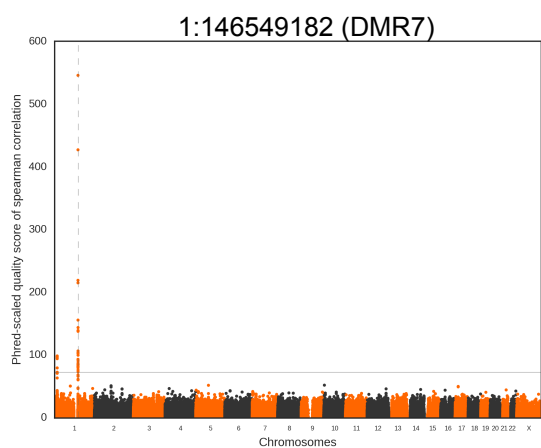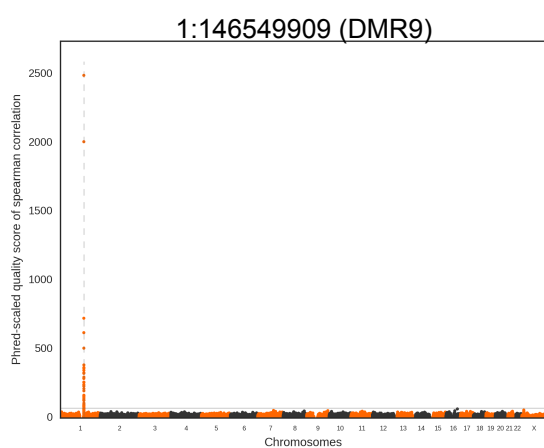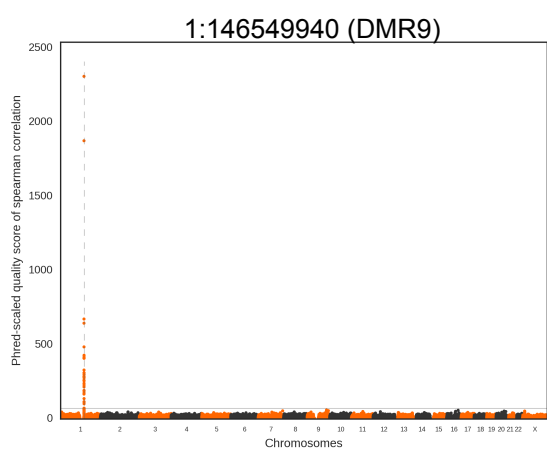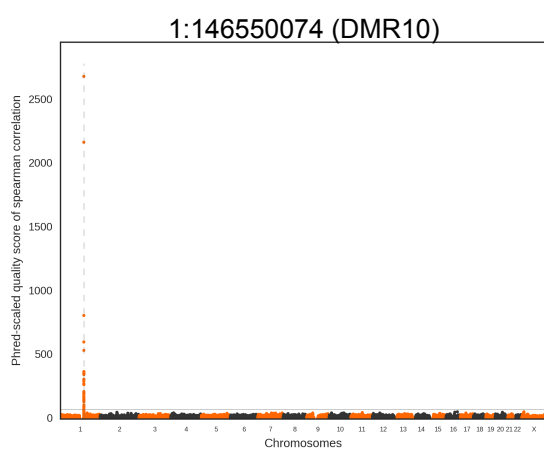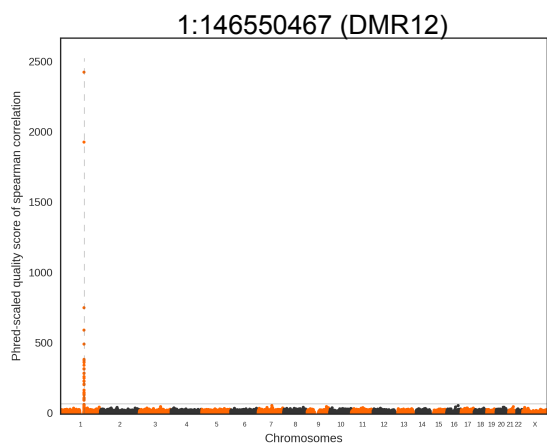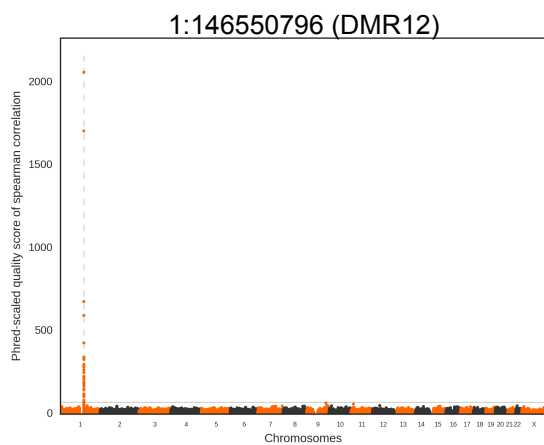

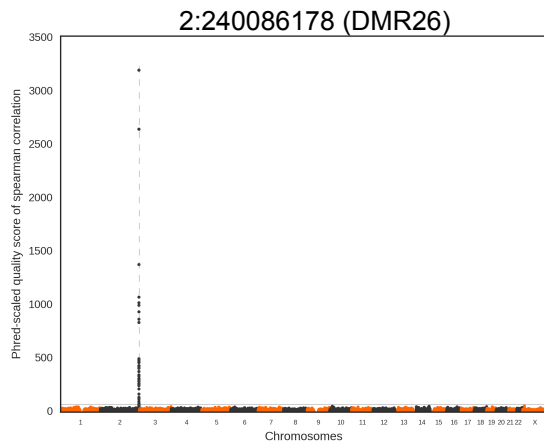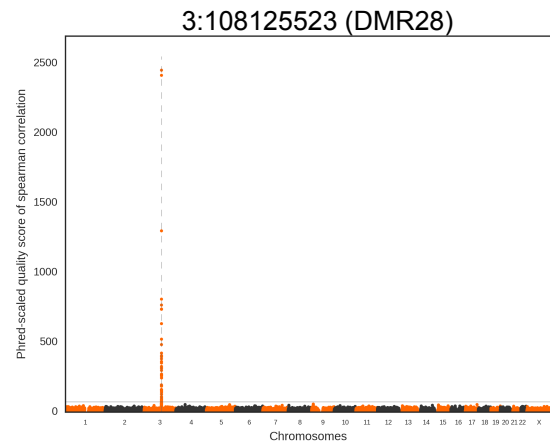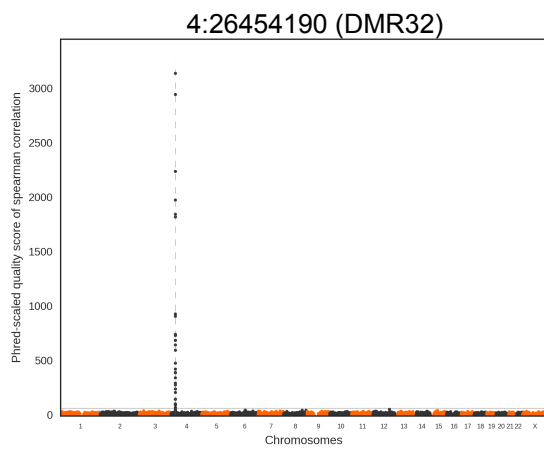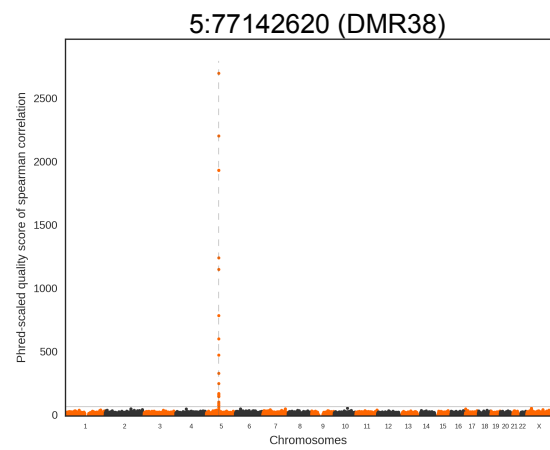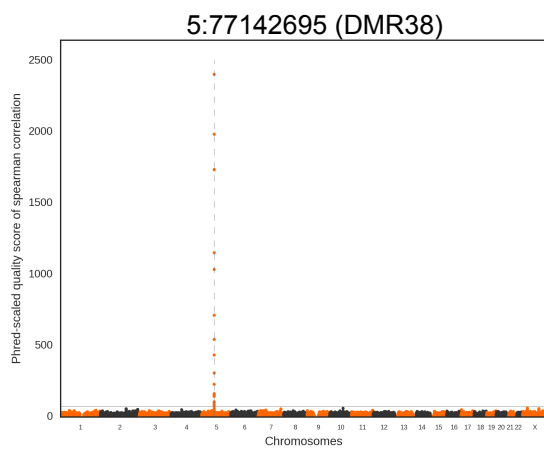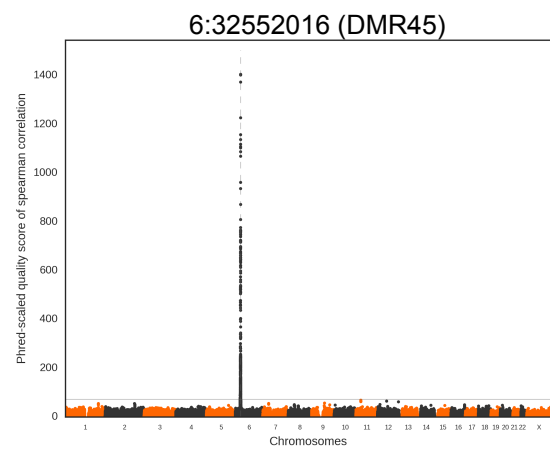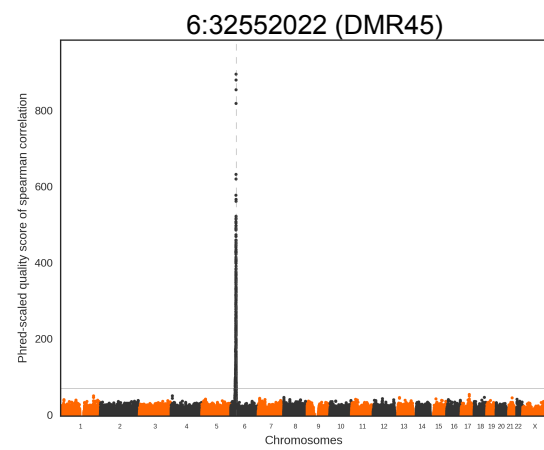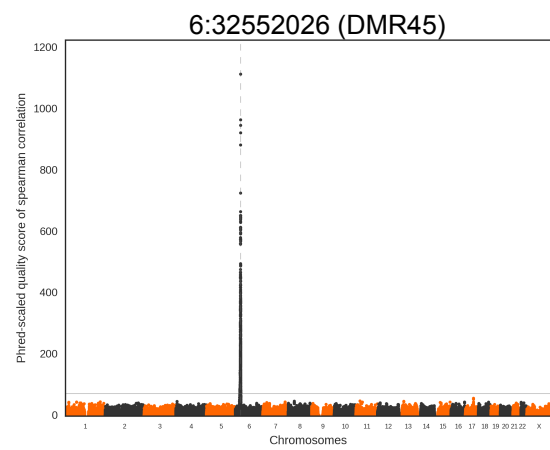

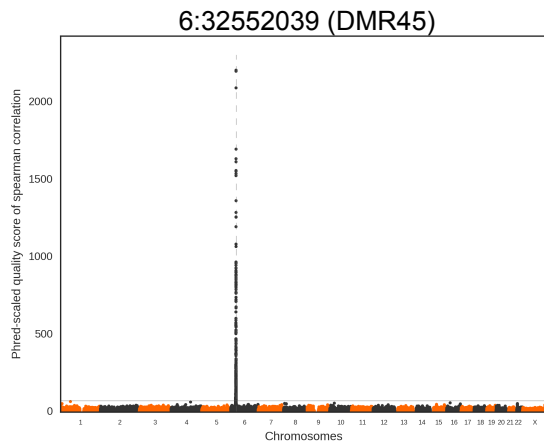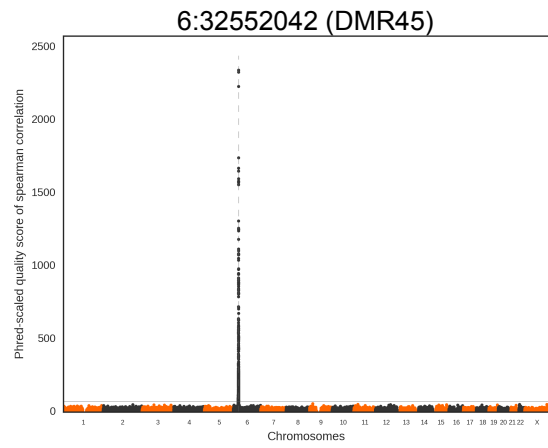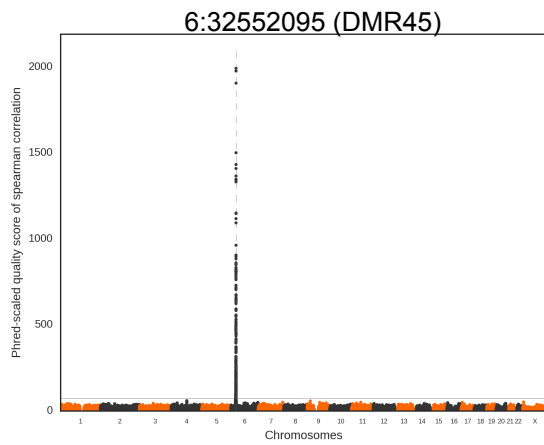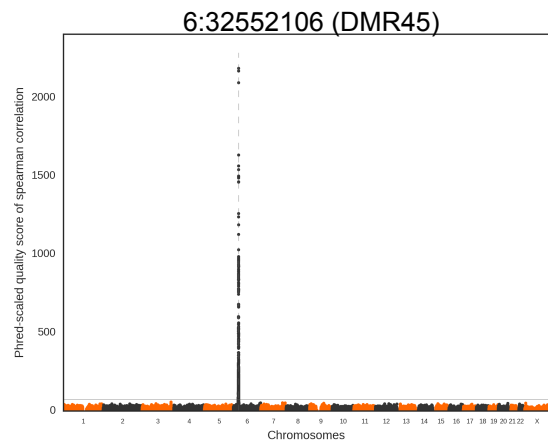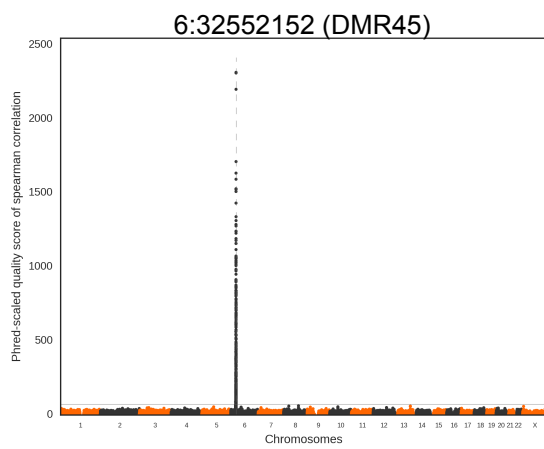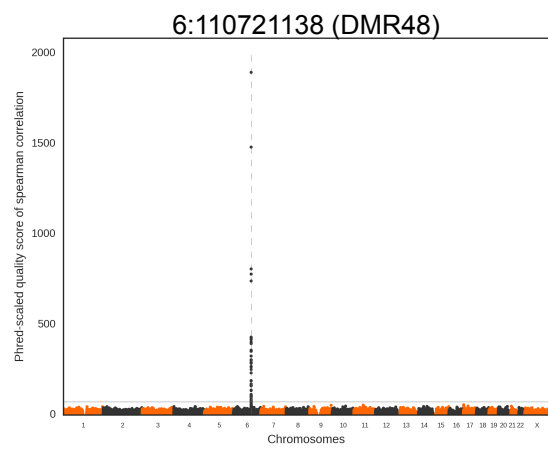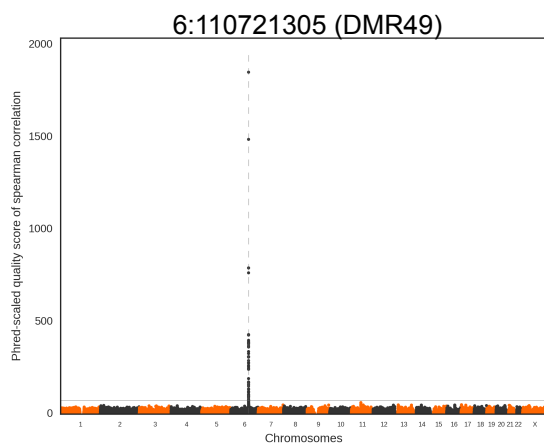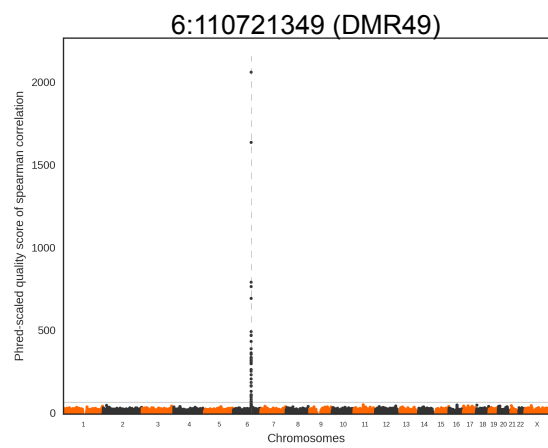

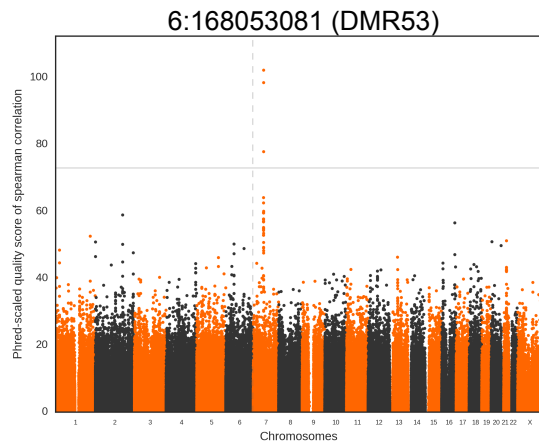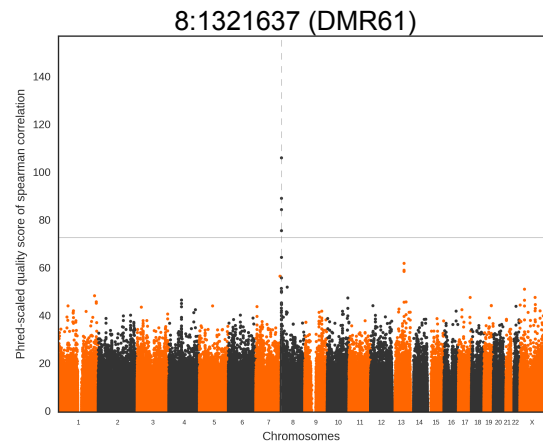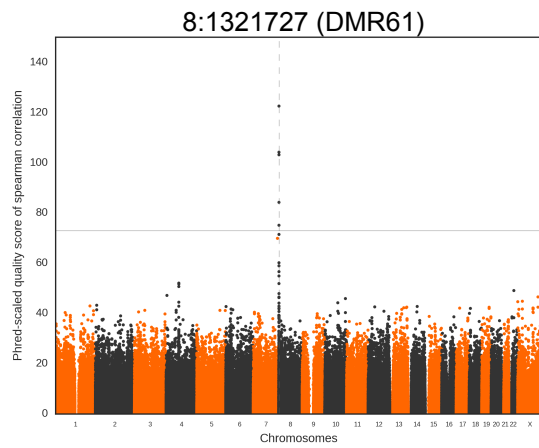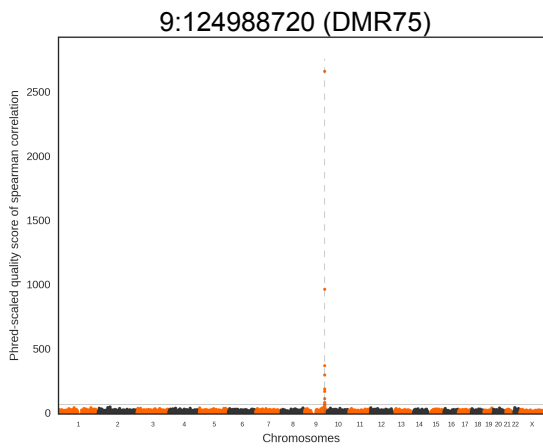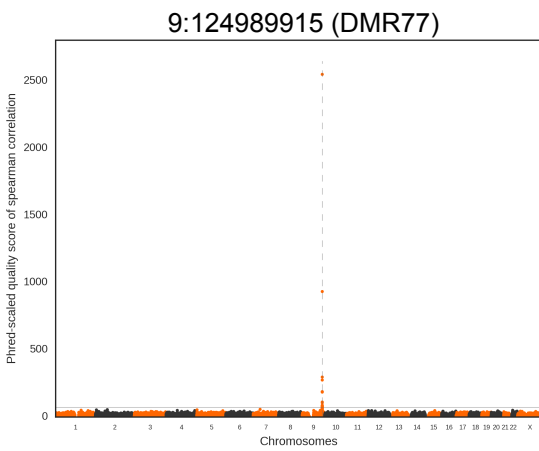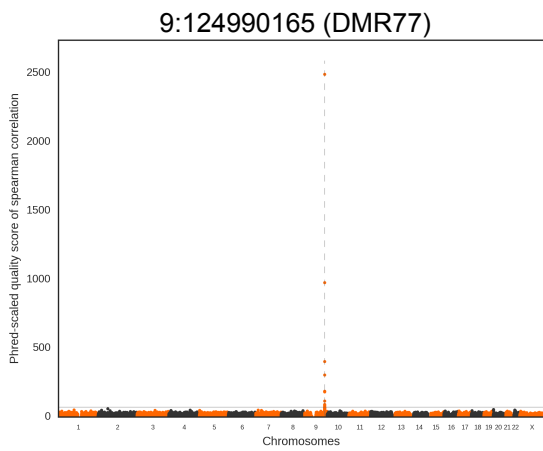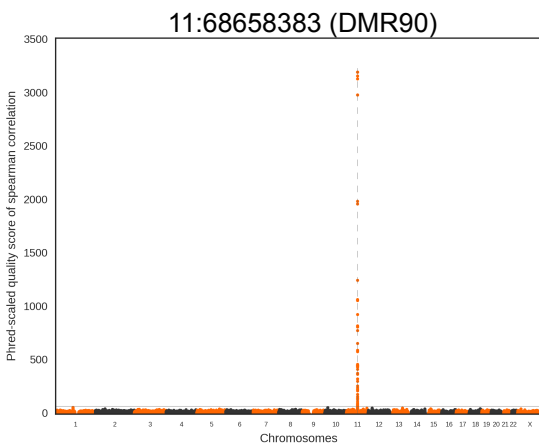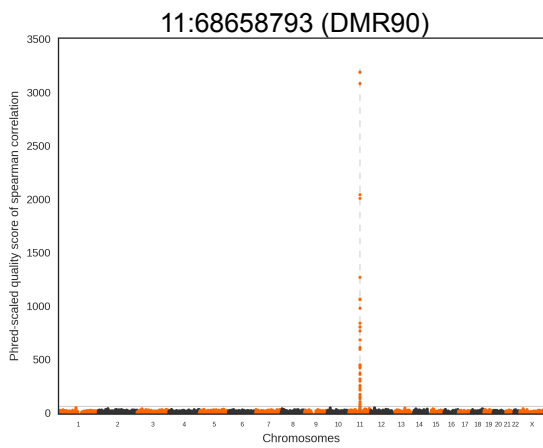

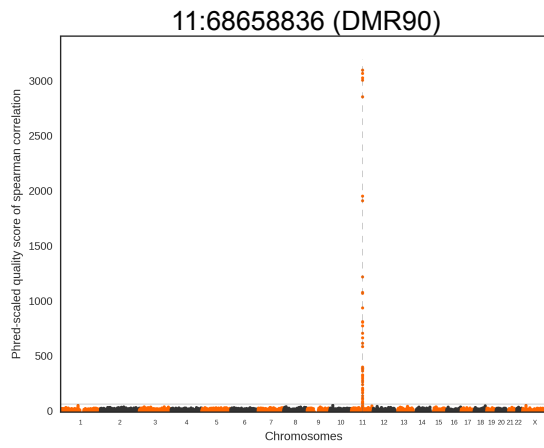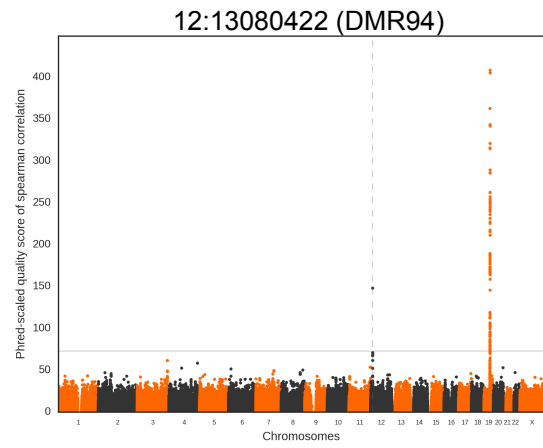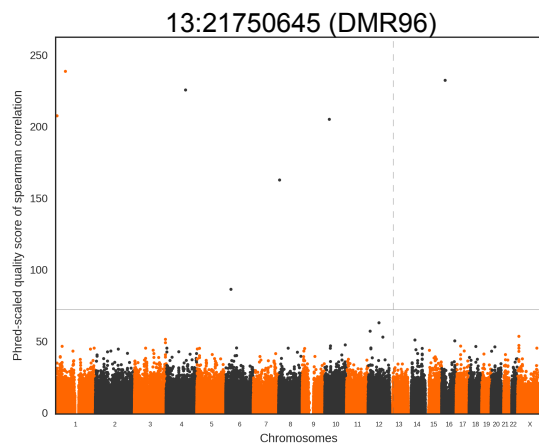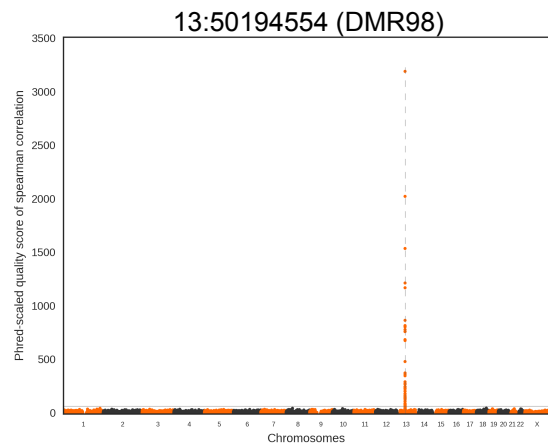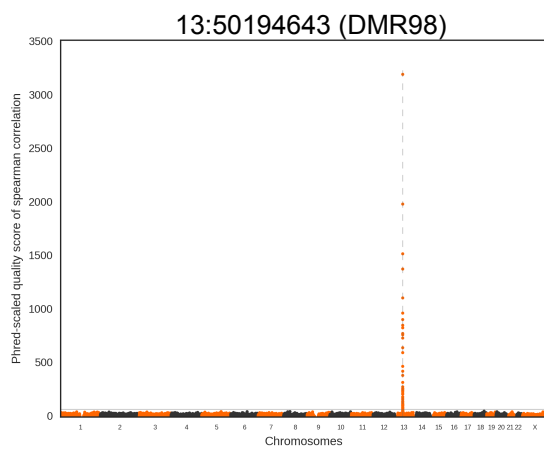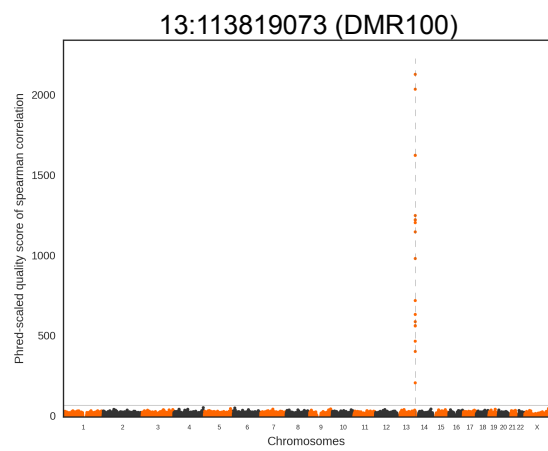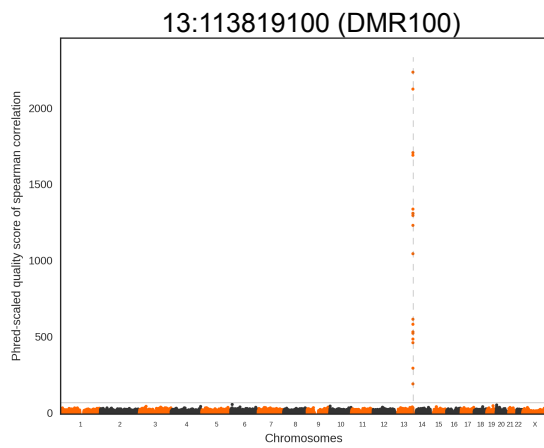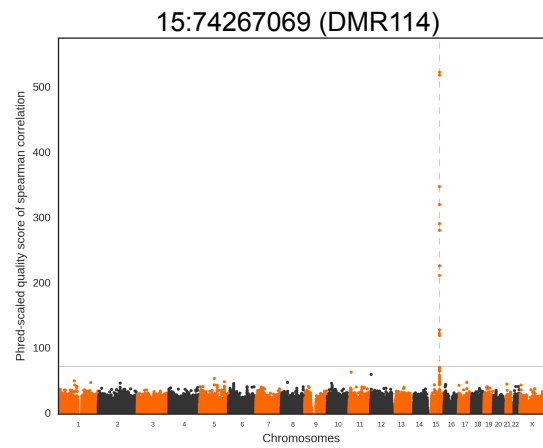

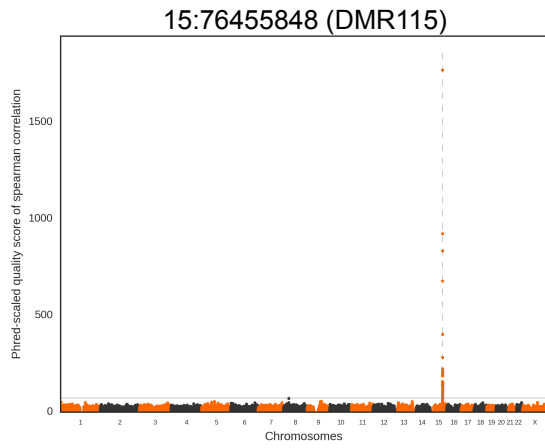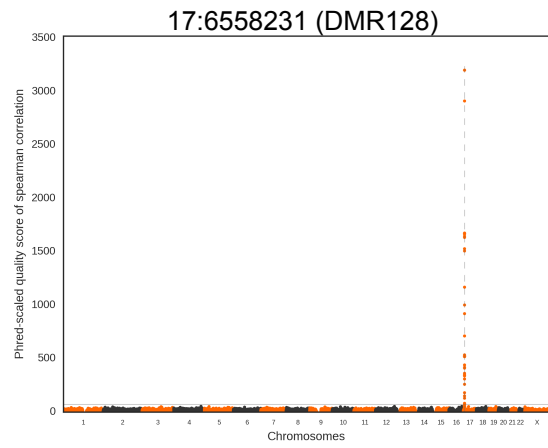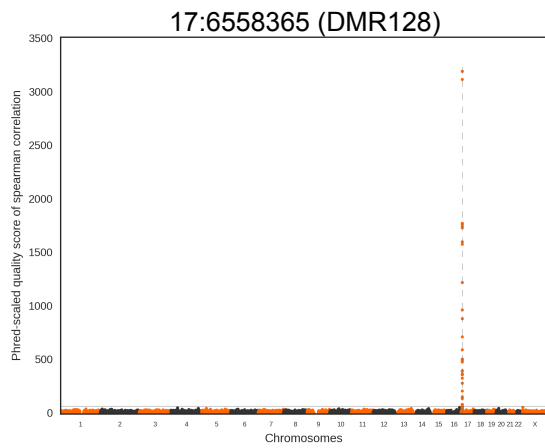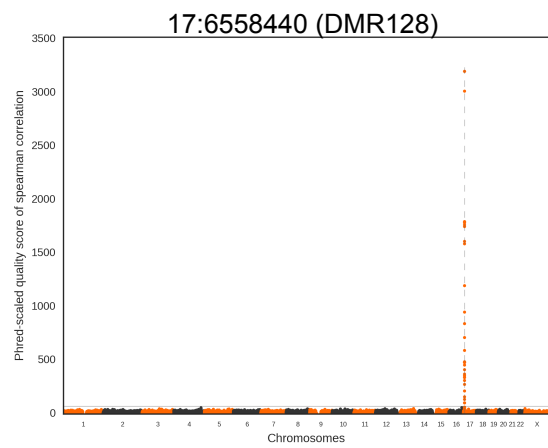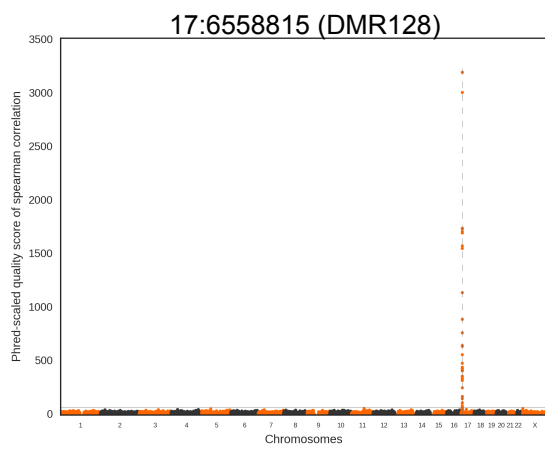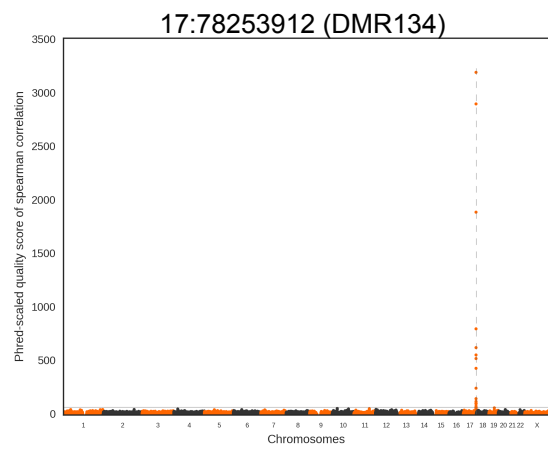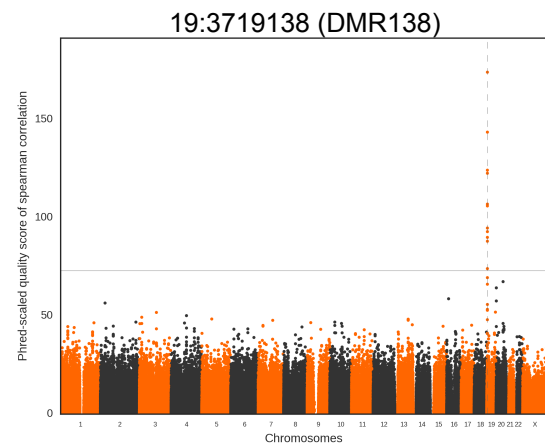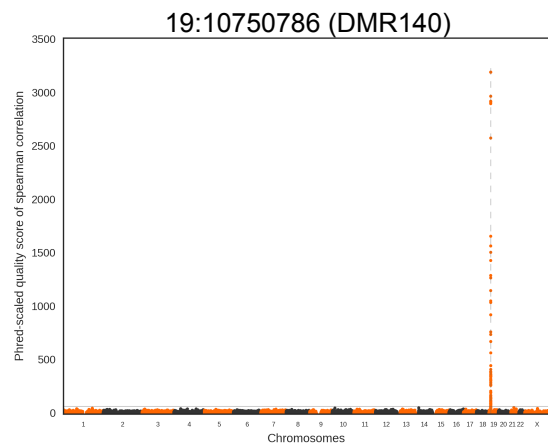

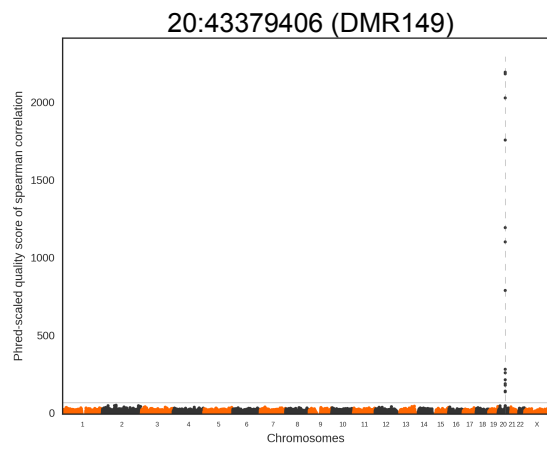

Supplement: Supplementary file 17 — Additional file 17. Manhattan plots of GWASs. Dashed vertical line DMR position. Horizontal line GWAS significance threshold. [file 13072_2017_144_MOESM17_ESM.pdf]

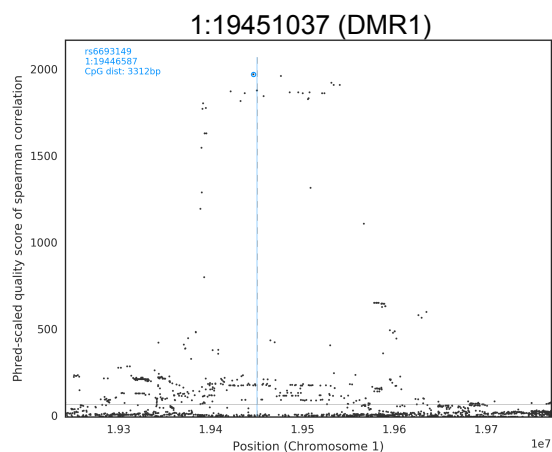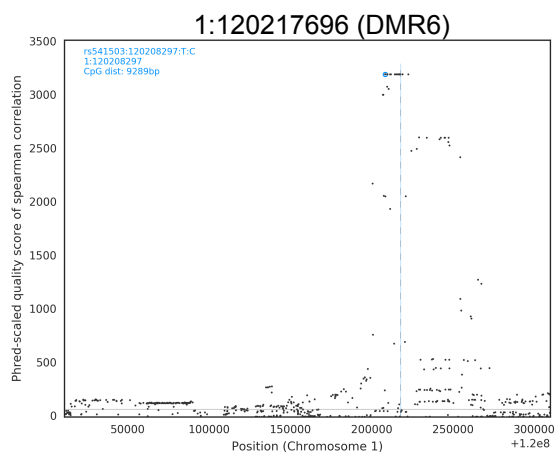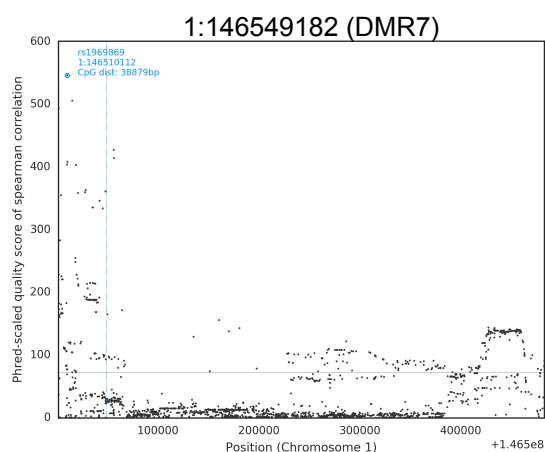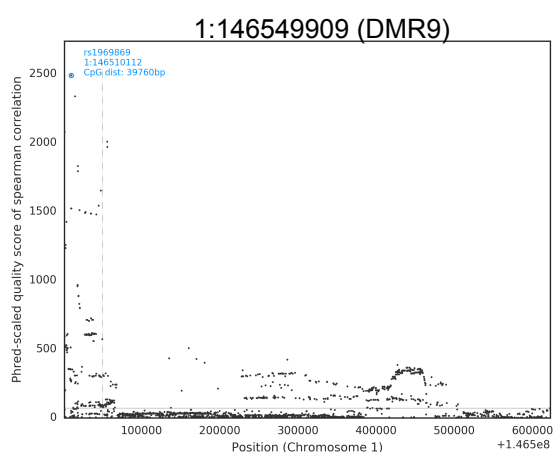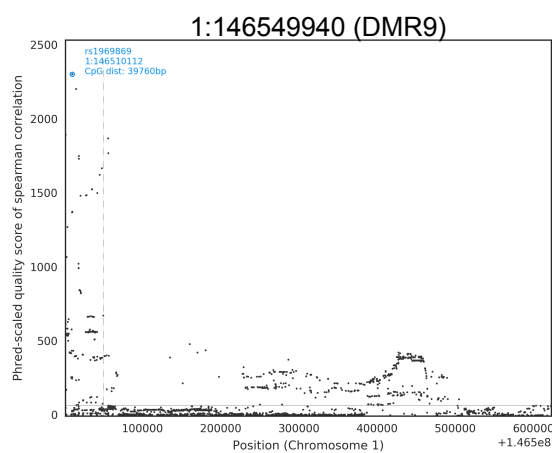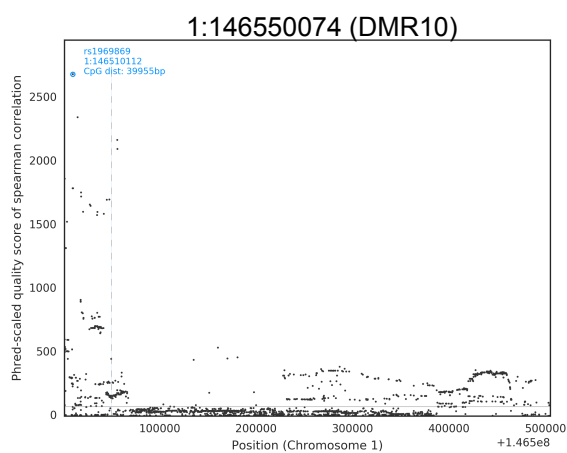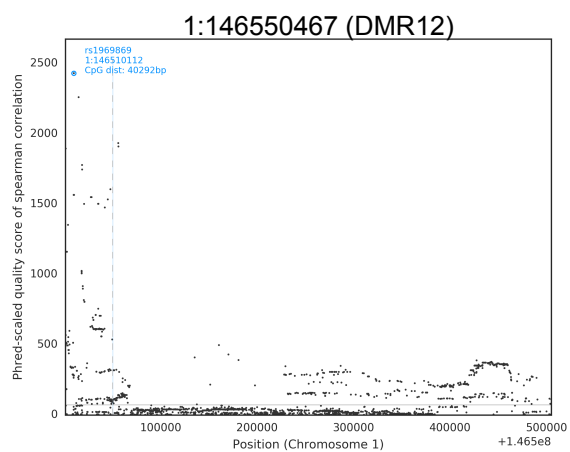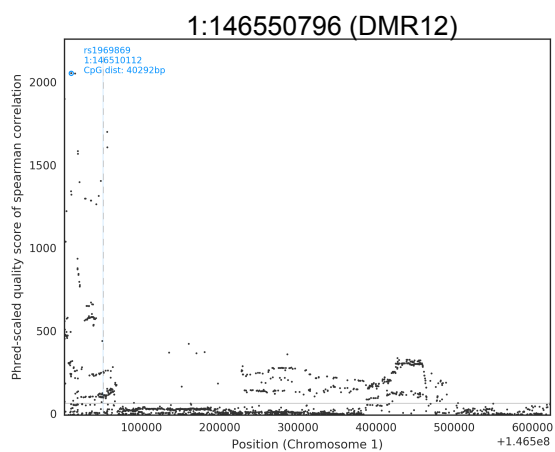

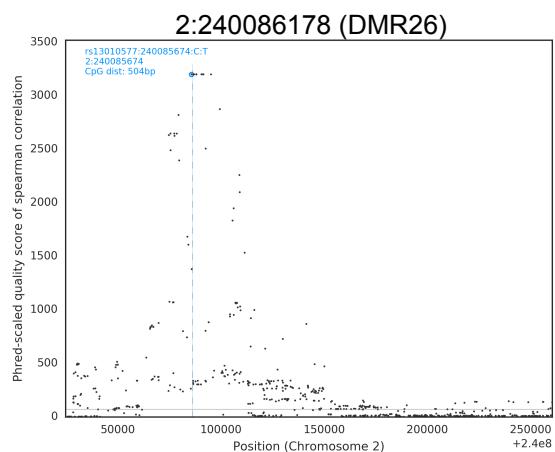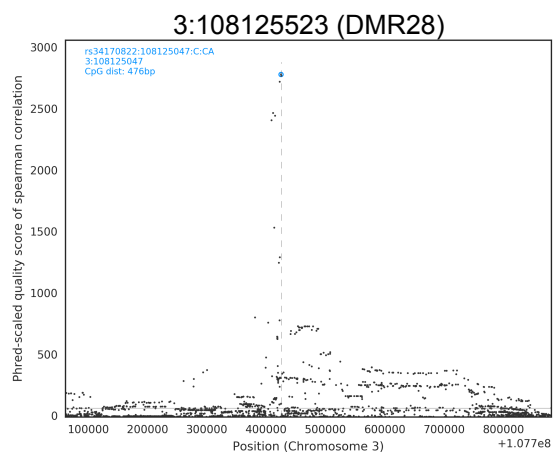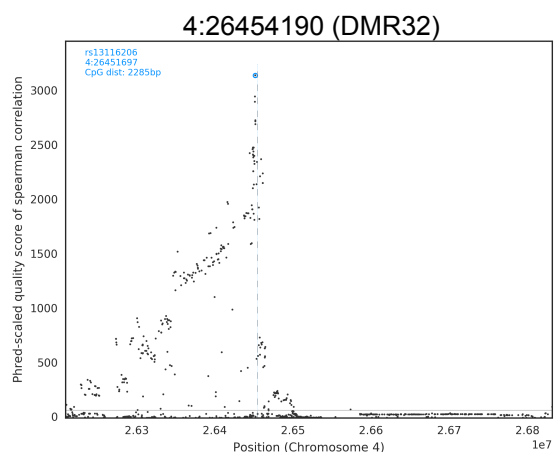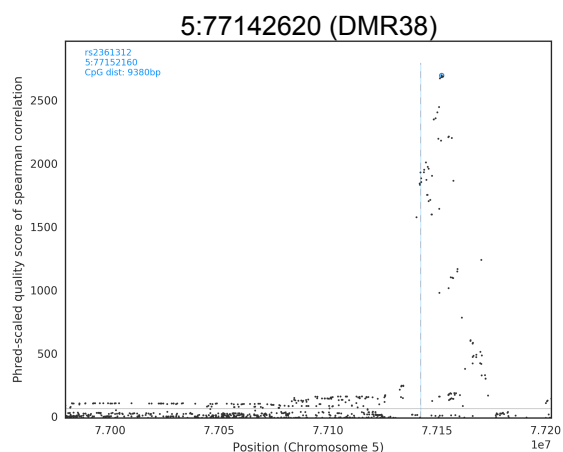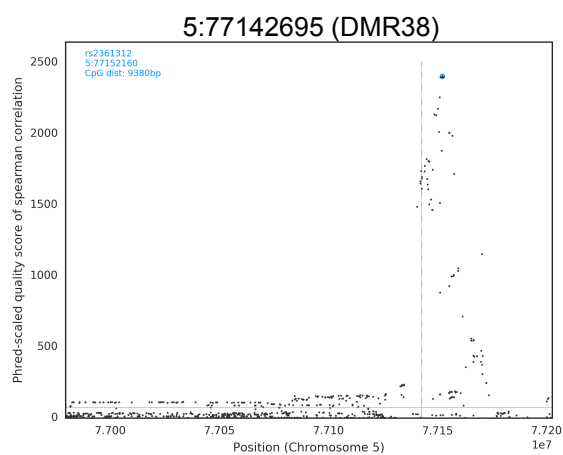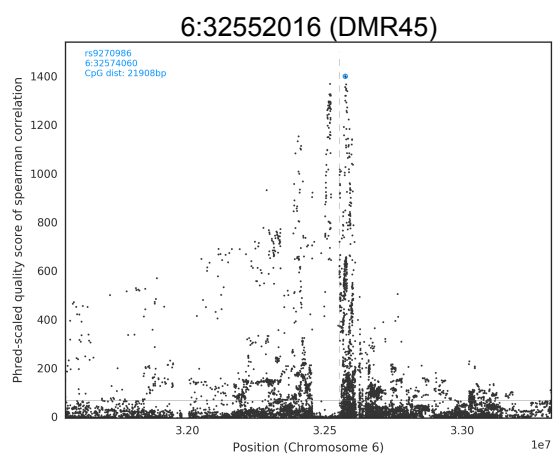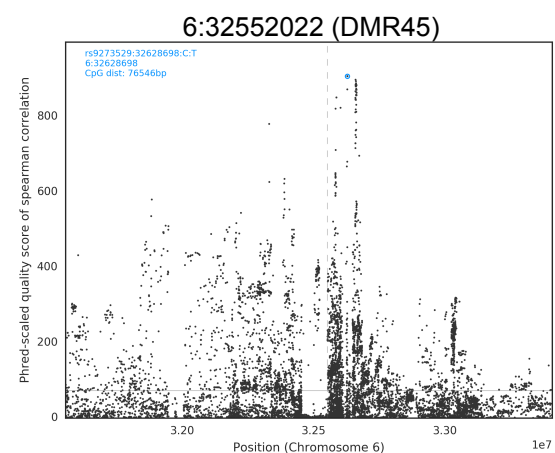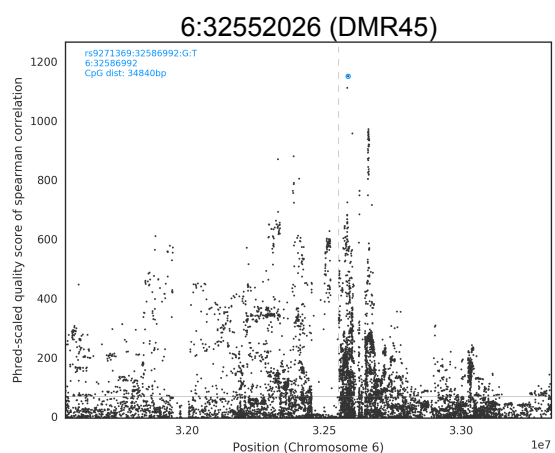

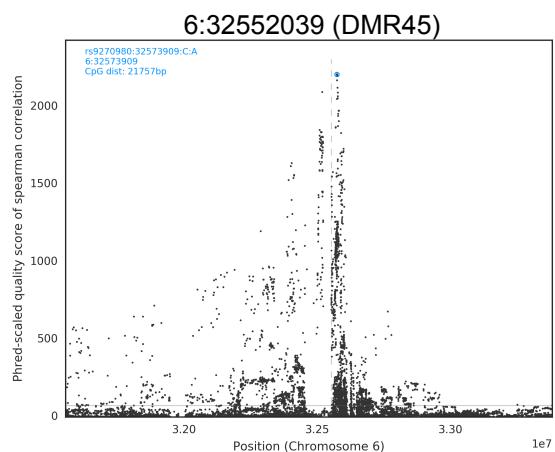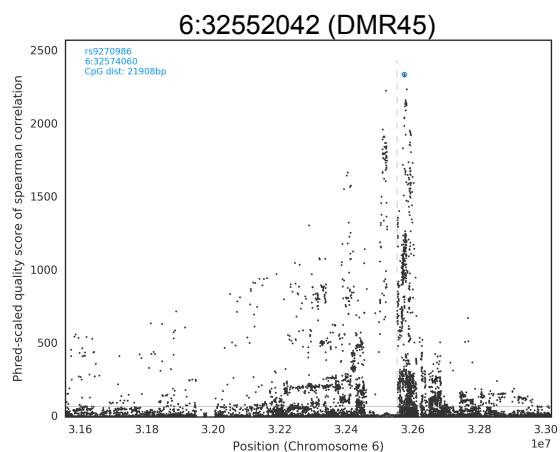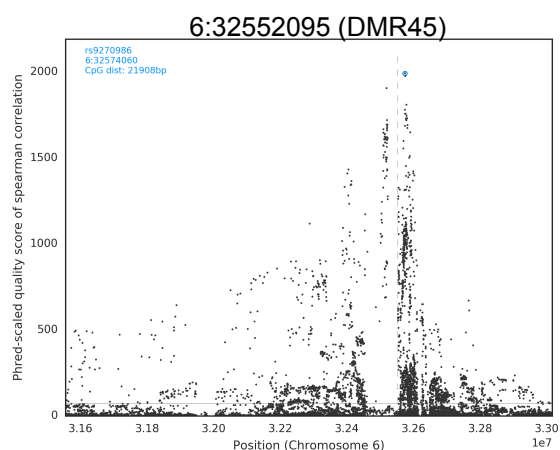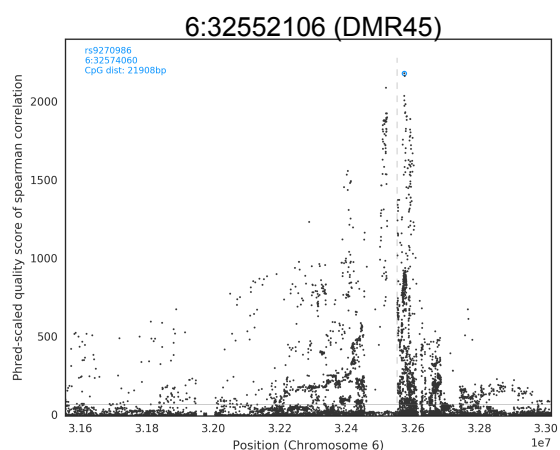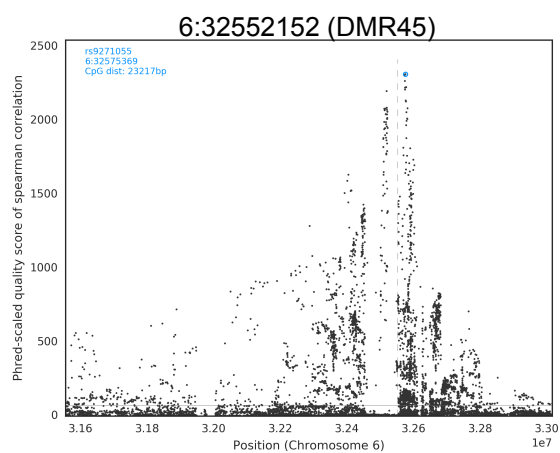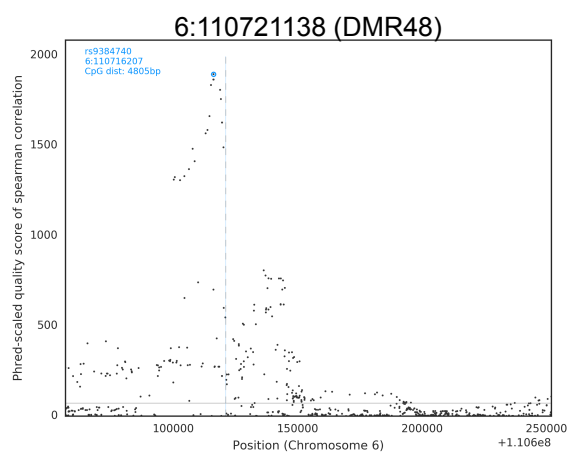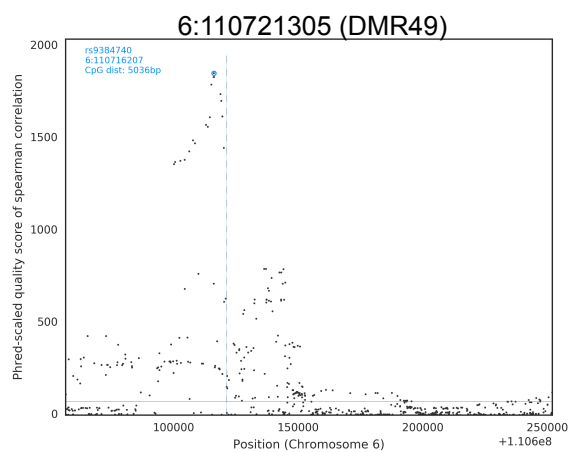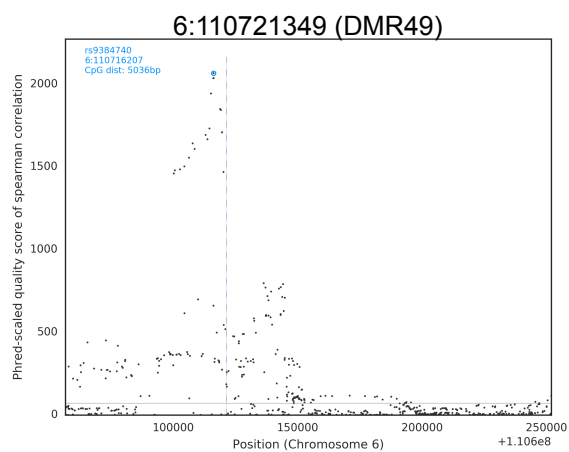

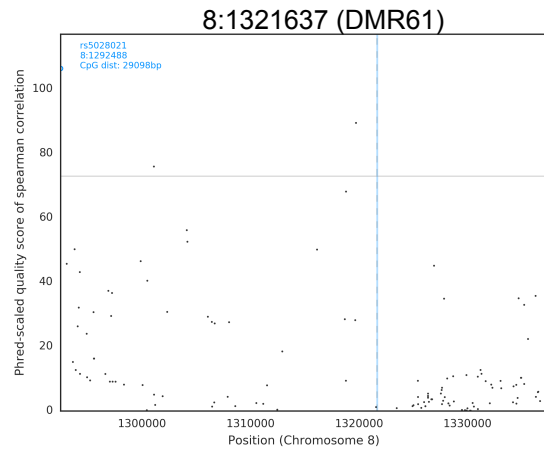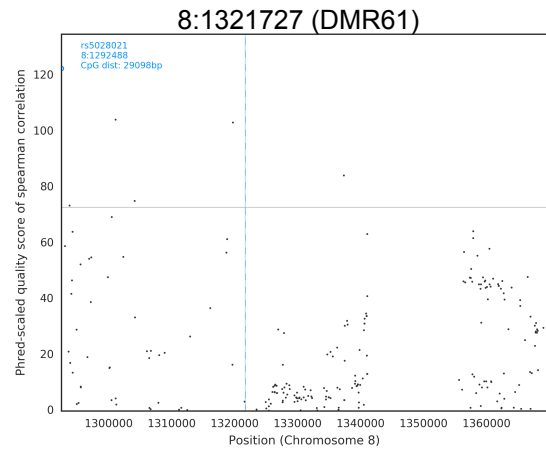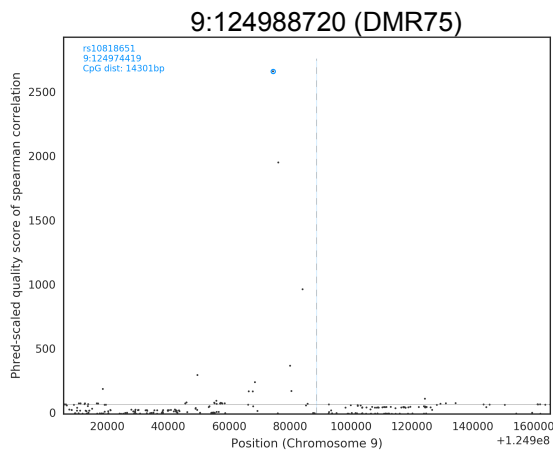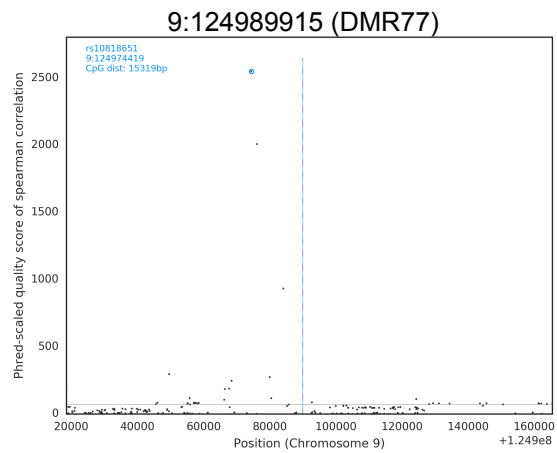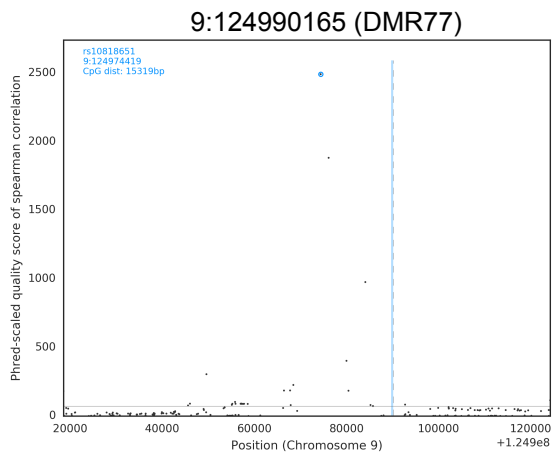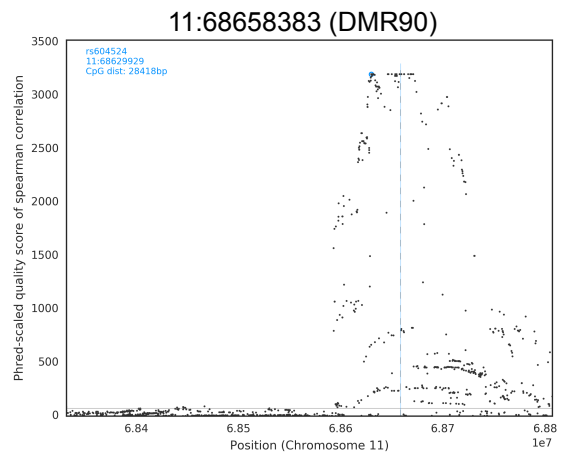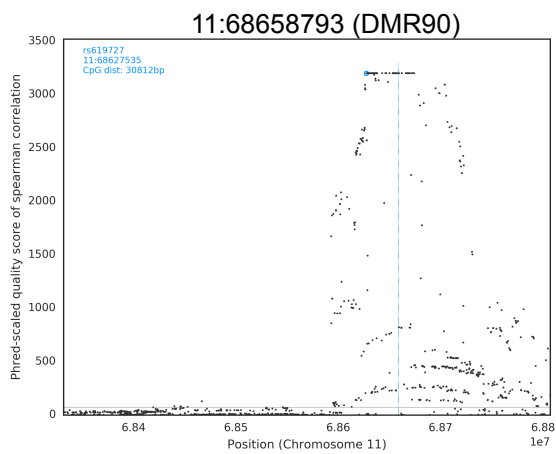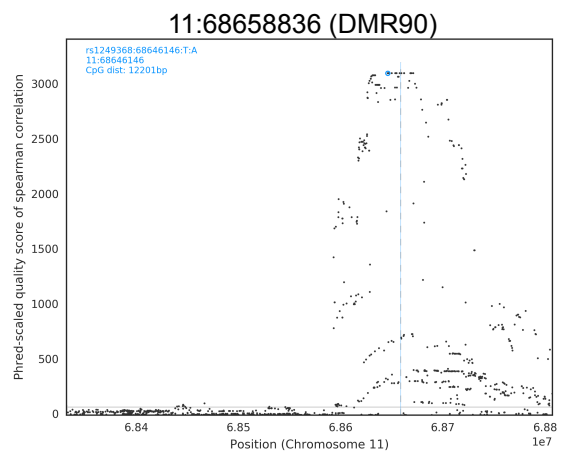

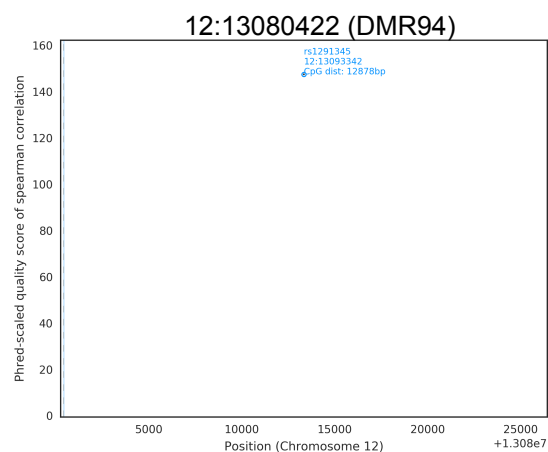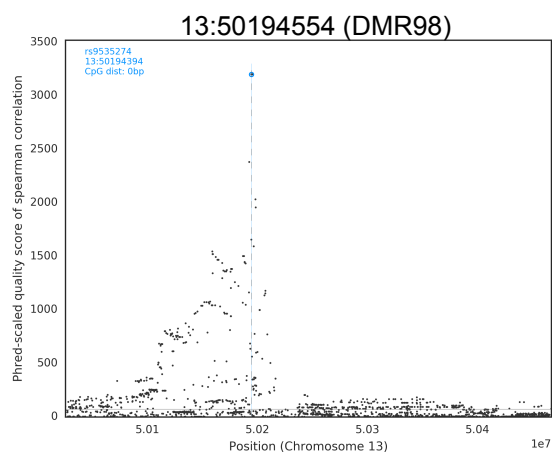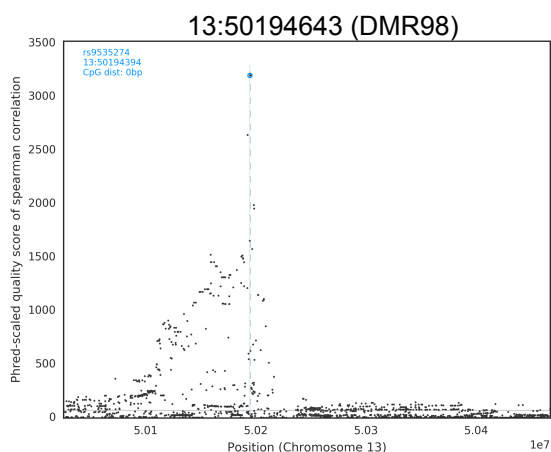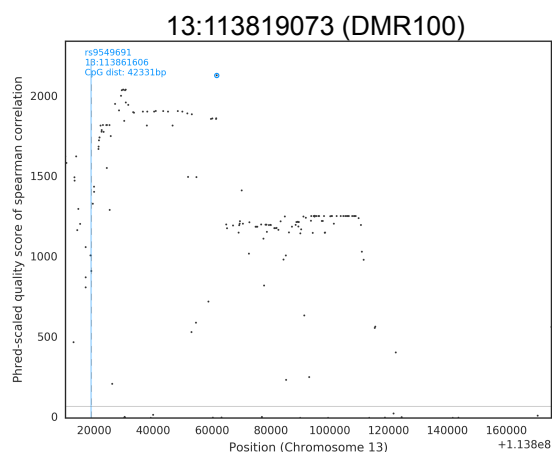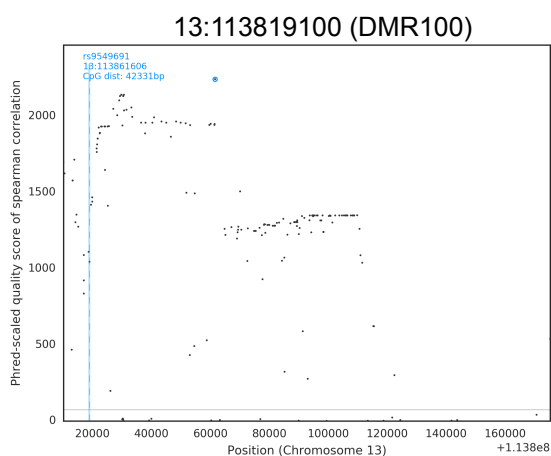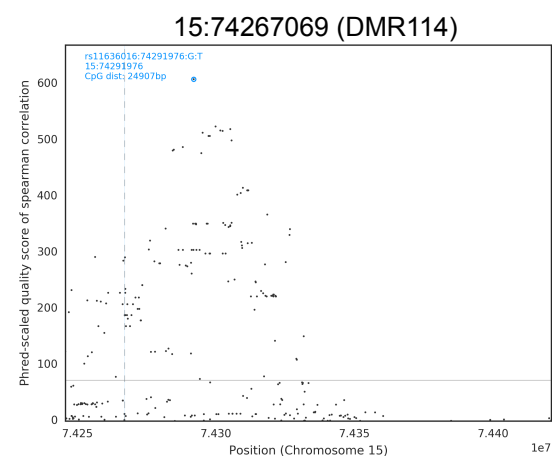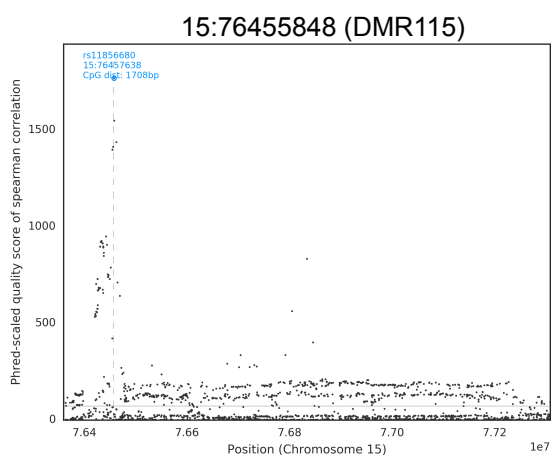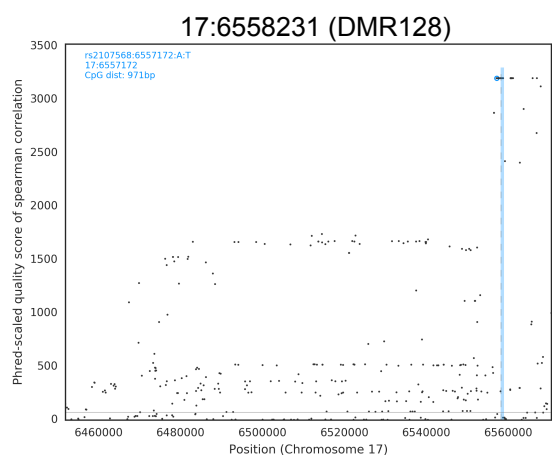

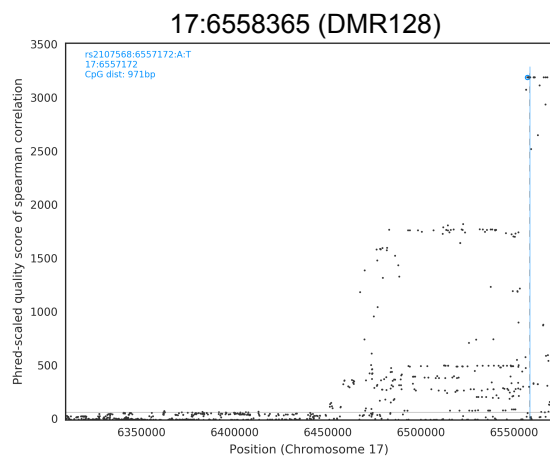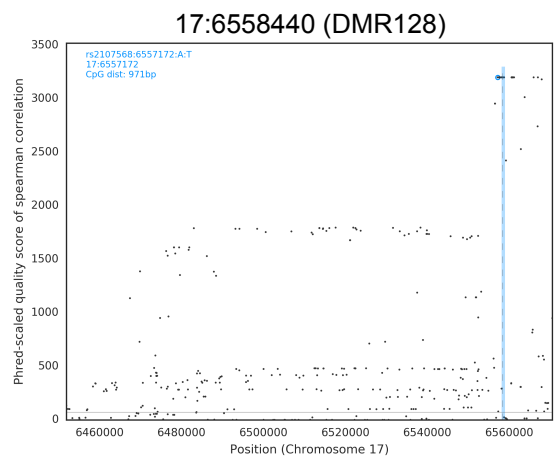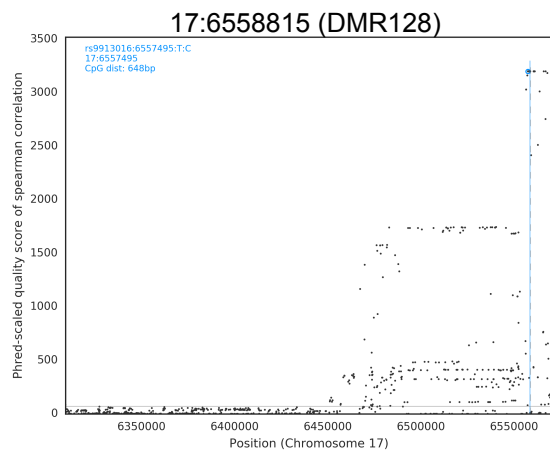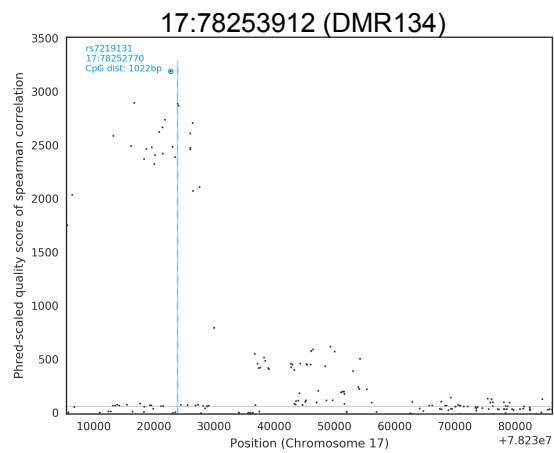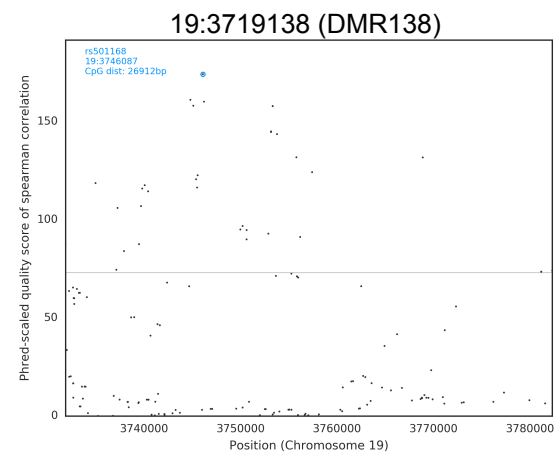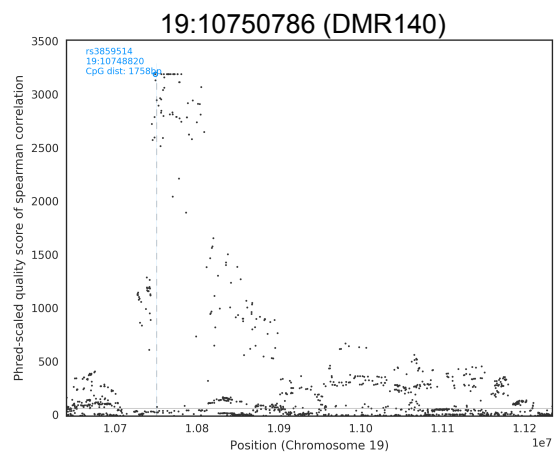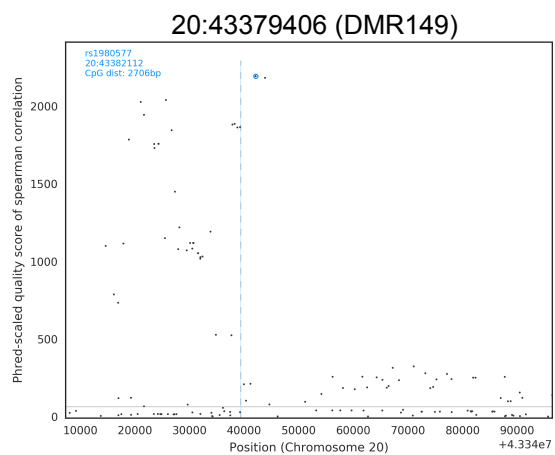

Supplement: Supplementary file 18 — Additional file 18. Zoom-ins with imputed SNPs. Blue lead-SNP. Dashed vertical line DMR position. Horizontal line GWAS significance threshold. [file 13072_2017_144_MOESM18_ESM.pdf]

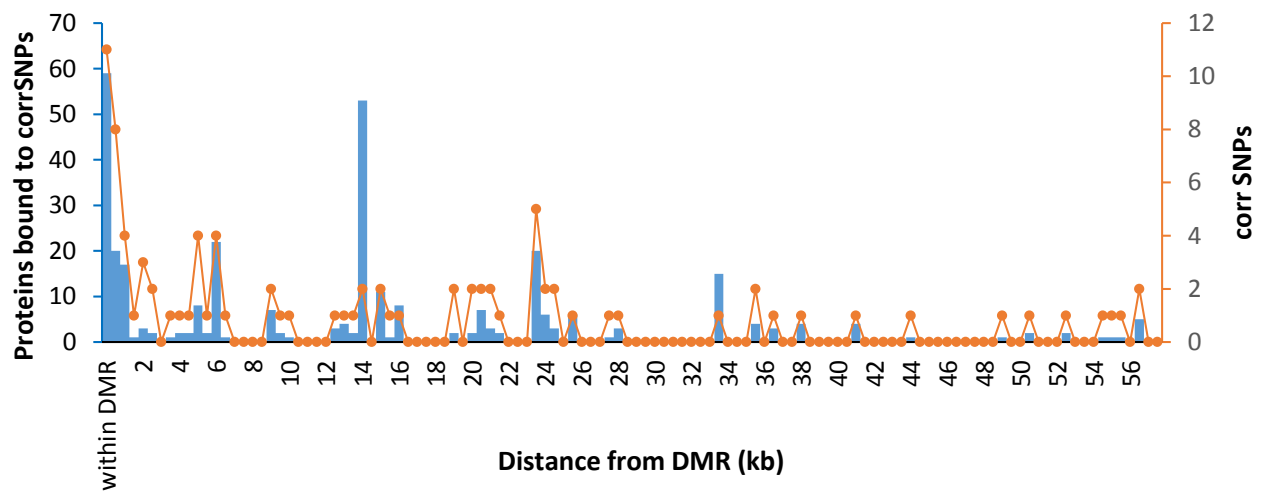

Supplement: Supplementary file 20 — Additional file 20. Distance of SNPs with known binding proteins to the corresponding DMR border. Number of known proteins binding to lead-SNPs or to SNPs in high LD with the lead-SNPs vs. their distance to the corresponding DMR border. Data from Encode ChIPseq obtained via the HaploReg database. [file 13072_2017_144_MOESM20_ESM.pdf]
