# Supplementary material for: Regions of common inter-individual DNA methylation differences in human monocytes: genetic basis and potential function
Source: Epigenetics Chromatin. 2017 Jul 26;10:37. doi: 10.1186/s13072-017-0144-2 (PMC5530492; doi:10.1186/s13072-017-0144-2)
Supplement: Supplementary file 2 — Additional file 2. Quality parameters of WGBS datasets. [file 13072_2017_144_MOESM2_ESM.docx]

**Additional file 2: Quality parameters of WGBS data sets.**

| Sample | Mapping Efficiency | Duplication Rate | Conversion Rate | Coverage (X) |
| --- | --- | --- | --- | --- |
| M55900_Ct_WGBS_E | 0.99 | 0.22 | 0.99 | 40.23 |
| Hm01_BIMo_Ct_WGBS_E | 0.98 | 0.15 | 0.99 | 35.26 |
| Hm02_BlMo_Ct_WGBS_E | 0.97 | 0.14 | 0.99 | 24.46 |
| Hm03_BlMo_Ct_WGBS_E | 0.98 | 0.07 | 0.99 | 34.45 |
| Hm05_BlMo_Ct_WGBS_E | 0.97 | 0.08 | 0.99 | 27.68 |
