# Supplementary material for: Regions of common inter-individual DNA methylation differences in human monocytes: genetic basis and potential function
Source: Epigenetics Chromatin. 2017 Jul 26;10:37. doi: 10.1186/s13072-017-0144-2 (PMC5530492; doi:10.1186/s13072-017-0144-2)
Supplement: Supplementary file 8 — Additional file 8. Under- and overrepresentation of chromatin states. [file 13072_2017_144_MOESM8_ESM.docx]

**Additional file 7: Under- and overrepresentation of chromatin states**

| **Donor** | **underrepresented** | **p-value** | **overrepresented** | **p-value** |
| --- | --- | --- | --- | --- |
| **Hm03** | 1_TssA | 7.3·10^-4^ | 16_ReprPC | 0.040 |
|  | 17_ReprPCWk | 0.017 | 4_TssFlnkD | 0.001 |
|  | 12_ZNF/Rpts | 0.026 |  |  |
|  | 5_Tx | 0.041 |  |  |
| **Hm05** | 17_ReprPCWk | 0.011 | 9_EnhA1 | 0.005 |
|  | 1_TssA | 5.3·10^-5^ | 15_EnhBiv | 0.041 |
|  | 5_Tx | 6.23·10^-4^ | 16_ReprPC | 0.002 |
|  | 13_Het | 0.035 | 2_TssFlnk | 0.038 |
|  |  |  | 8_EnhG2 | 0.007 |
