# Supplementary material for: Regions of common inter-individual DNA methylation differences in human monocytes: genetic basis and potential function
Source: Epigenetics Chromatin. 2017 Jul 26;10:37. doi: 10.1186/s13072-017-0144-2 (PMC5530492; doi:10.1186/s13072-017-0144-2)
Supplement: Supplementary file 21 — Additional file 21. TRANSFAC motif enrichment in 501 SNP regions (SNP ± 100 bp). Yes and No denote the relative number of sites for the selected matrix in the DMRs as compared to the background dataset. [file 13072_2017_144_MOESM21_ESM.docx]

**Additional file 20: TRANSFAC motif enrichment in 501 SNP regions (SNP ±100 bp).**

| **Factor name** | **Yes** | **No** | **Yes/No** |
| --- | --- | --- | --- |
| **CREB group** | 0.0155 | 0.0015 | 10.174 |
| **NF-1 factors** | 0.013 | 0.0015 | 8.478 |
| **Sp100** | 0.0259 | 0.0076 | 3.3912 |
| **CTCF** | 0.0311 | 0.0107 | 2.9067 |

Yes and No denote the relative number of sites for the selected matrix in the DMRs as compared to the background data set.
